# A cellular view of drought adaptation in sugarcane: multi-omics integration reveals a quadruple module network linking water regulation, oxidative defense, cell wall remodeling, and cell cycle regulation

**DOI:** 10.7717/peerj.21396

**Published:** 2026-06-17

**Authors:** Jiahui Lu, Keyang Dai, Zhigang Li, Haiwei Chu, Shijian Han, Liyi Chen, Meiyan Wu, Suli Li

**Affiliations:** 1 Guangxi Key Laboratory of Sugarcane Biology, College of Agriculture, Guangxi University, Nanning, Guangxi, China; 2 Key Laboratory of Crop Cultivation and Tillage, College of Agriculture, Guangxi University, Nanning, Guangxi, China

**Keywords:** Suspension cells, PEG-6000, Cell viability, Ultrastructure, Physiology, Transcriptomics

## Abstract

*Saccharum* spp., a globally important crop for sugar and bioenergy production, suffers substantial yield losses due to drought. Whole-plant studies are complicated by inherent tissue heterogeneity and organ-level interactions. To dissect this complexity, we established a homogeneous suspension cell line from the drought-tolerant cultivar ‘Roc22’. A gradient of polyethylene glycol 6000 (PEG-6000)-induced drought stress (0%, 5%, 10%, 20%) was applied, enabling integrated cytological, physiological, and transcriptomic analysis. Cytological analysis revealed a concentration-dependent decline in cell viability under drought stress, with survival rates dropping to 45.5% after 8 h of 20% PEG treatment. Cells exhibited browning, shrinkage, and ultrastructural damage (affecting the plasma membrane, mitochondria, and nucleus), with severe stress triggering programmed cell death (PCD). Physiological analysis showed that malondialdehyde (MDA) content peaked under 10% PEG treatment (17.16 μmol g^−1^ FW, representing a 68.55% increase over the control), soluble sugar content accumulated progressively with stress intensity (increasing by 45.64% at 20% PEG), while soluble protein content decreased significantly (a reduction of 82.9% at 20% PEG). Antioxidant enzyme activities responded differentially. Transcriptome profiling identified 9,923 differentially expressed genes (DEGs). Protein-protein interaction (PPI) network analysis pinpointed 21 core candidates, including aquaporins (*e.g.*, *TIP3-1*, *NIP1-1*), cell wall modifiers (*e.g., XTH23*, *CESA1*), peroxidases (*e.g.*, *PER25*), and antioxidant enzymes (*e.g.*, *APX1*, which was strikingly up-regulated 6.22-fold).This study provides a systematic dissection of th e multi-dimensional drought response network in sugarcane at the cellular level, integrating water regulation, oxidative defense, cell wall remodeling, and cell cycleregulation. Quantitative reverse transcription polymerase chain reaction (qRT-PCR) validation of nine candidate genes confirmed the reliability of the transcriptomic data. Our findings offer deep mechanistic insights and establish a novel “cell model + multi-omics” paradigm for crop stress biology research.

## Introduction

Sugarcane (*Saccharum* spp.), a vital crop for sugar, biofuel, and fiber production, is highly susceptible to drought, which can cause yield losses exceeding 60% (Basnayake et al., 2012). Cultivating drought-tolerant varieties is therefore crucial for sustainable production.

Drought stress triggers complex physiological and molecular responses in sugarcane. Physiologically, it reduces stomatal conductance, photosynthetic rate, and chlorophyll content, while inducing oxidative stress characterized by reactive oxygen species accumulation (Kumar et al., 2023; Ferreira et al., 2017). To counteract this, sugarcane activates antioxidant enzymes (superoxide dismutase (SOD), catalase (CAT), peroxidase (POD)) and accumulates proline and soluble sugars for osmotic adjustment (Bavai et al., 2025). At the molecular level, transcriptomic analyses have revealed thousands of differentially expressed genes involved in photosynthesis, hormone signaling, carbohydrate and amino acid metabolism (Wang et al., 2025; Kumar et al., 2023). Drought-inducible transcription factor families (bHLH, bZIP, ERF, NAC, MYB, WRKY) and key genes encoding Late embryogenesis abundant (LEA) proteins, dehydrins, aquaporins, and GST serve as core components of the drought response network (Wang et al., 2025; Kumar et al., 2023; Wang et al., 2023). Additionally, calcium-mediated signaling pathways involving CAMTA transcription factors have been implicated in drought adaptation (Silva et al., 2025).

Conventional studies using whole plants, organs, or tissues, while informative, are often confounded by genetic heterogeneity, tissue specificity, and environmental variability (Song et al., 2021). In contrast, suspension cell cultures provide a model system with exceptional uniformity, scalability, and controllability, enabling a consistent and coordinated response to stress under precisely defined conditions (Song et al., 2021). This unique advantage makes suspension cells an indispensable tool for mechanistic investigations across diverse fields, including cytology (Zhu, 2022), developmental biology (Deo et al., 2010), abiotic stress (Song et al., 2021), secondary metabolism (Nomura & Kato, 2025), ion transport (Zhang et al., 2005), signaling (Sánchez-Sandoval et al., 2021), and molecular bioreactors (Wang et al., 2024).

The utility of suspension cells in deciphering intricate abiotic stress mechanisms is well-demonstrated across diverse plant species. Pioneering studies have leveraged this system to uncover key cellular responses to hyperosmotic and oxidative stress in tobacco (Skrzypczak et al., 2025; Chen et al., 2024), drought stress in rice (Yan et al., 2024), salt stress in Haplophyllum virgatum and Jerusalem artichoke (Abedi et al., 2023; Song et al., 2021), as well as heat and cold stress in Arabidopsis and grape (Aristizábal et al., 2019; Wang et al., 2018). These collective efforts validate suspension cells as a powerful platform for elucidating stress adaptation strategies, encompassing signaling, gene regulation, metabolic changes, and organelle function.

As with many other crops, numerous reports have described the effects of drought stress on sugarcane at the plant (Khonghintaisong et al., 2024), organ (Gai et al., 2024), or tissue (Abdelsalam et al., 2021) level; however, studies at the cellular level remain scarce. To improve the genetic basis of drought tolerance in sugarcane, this study established a stable suspension cell line from a drought-tolerant sugarcane cultivar ‘ROC22’ and applied a multi-omics approach to investigate polyethylene glycol 6000 (PEG-6000)-induced drought responses. Our study integrates cytological, physiological, and transcriptomic analyses with the specific objectives of: (1) characterizing drought-induced morphological and ultrastructural changes at the cellular level; (2) quantify the dynamic changes of oxidative damage markers, osmoregulation, and antioxidant enzymes; and (3) identify drought-responsive genes and regulatory networks by RNA sequencing and functional enrichment. The results of this study systematically revealed the multi-omics response network of sugarcane to drought stress at the cellular level for the first time.

## Methods and Materials

### Plant material and suspension culture establishment

The drought-tolerant sugarcane variety Roc22 (*Saccharum officinarum*) was planted at the sugarcane experimental base of Guangxi University. Young leaves from elongation-stage plants showing uniform growth and morphology were selected as explants for callus induction and suspension cell culture establishment. Immature leaf sheaths were collected on a clear day. The outer layers were removed, and the remaining tissues were disinfected with 75% ethanol for 30 s, followed by three rinses with sterile water. Under sterile conditions, the remaining leaf sheaths were carefully trimmed until the young yellowish leaves were exposed. The young leaves were then cut into thin segments approximately 0.5–1 cm in height and inoculated onto Murashige and Skoog (MS) (Murashige & Skoog, 1962) solid medium supplemented with 0.5 mg L^−1^ 2,4-dichlorophenoxyacetic acid (2,4-D), 0.5 mg L^−1^ 6-benzyladenine (6-BA), 30 g L^−1^ sucrose, and 6 g L^−1^ agar (pH 5.8). Callus formation was induced by culturing in dark environment at 26 ± 1 ^∘^C, and subculture was carried out every three weeks. After two generations of subculture, the calli with yellow color and loose texture were transferred to MS liquid medium containing 2,4-D (1.0 mg L^−1^) and sucrose (30 g L^−1^) (pH 5.8), and cultured in a 120 rpm shaker under dark conditions at 26 ± 1 °C. The cell suspensions were subcultured every 7 days to maintain exponential growth. Embryogenic suspension cells in the mid-log phase (4–5 days after subculture) with high viability (>90%) were used for all treatments (Gamborg, Miller & Ojima, 1968).

### Drought stress treatment and experimental design

Drought stress was simulated by supplementing the liquid culture medium with polyethylene glycol 6000, a non-penetrating osmoticum widely used to induce water deficit in plant cell cultures (Verslues et al., 2006). Cells were treated with four different concentrations of PEG-6000: 0% (control, CK), 5%, 10%, and 20% (w/v).

A preliminary time-course experiment was conducted to determine the appropriate treatment duration. Cell viability was monitored every 8 h for 72 h using the fluorescein diacetate (FDA) staining method (see ‘Cell viability testing’). Based on the cell viability results (see ‘Drought stress induces phenotypic changes and reduces cell viability in sugarcane suspension cells’), an 8-h treatment period was selected for subsequent experiments, as it represented a point where significant stress response was observed while maintaining sufficient cell viability for molecular and physiological analyses.

For the main experiment, cells from the same batch were collected and resuspended in media containing the respective polyethylene glycol (PEG) concentrations. All treatments were performed in triplicate (three biological replicates per treatment). After 8 h of treatment, cells were harvested by vacuum filtration, flash-frozen in liquid nitrogen, and stored at −80 ^∘^C for further analysis.

### Cell viability testing

Cell viability was assessed using the fluorescein diacetate (FDA; MACKLIN, Shanghai, China) staining method as described by Widholm (1972) with modifications. Briefly, 100 μL of cell suspension was incubated with two μL of FDA working solution (five mg mL^−1^ in phosphate buffer saline, PBS) in the dark for 5 min. Viable cells, exhibiting green fluorescence due to FDA hydrolysis by intracellular esterases, were immediately observed and counted under a fluorescence inverted microscope (Leica, Wetzlar, Germany). Cell viability was calculated as follows:

*Cell viability* (%) = (*Number of fluorescent cells*/*Total number of cells*) × 100%.

### Morphological and ultrastructural observation

For light microscopy, cells from each treatment were directly observed under an inverted microscope (Leica Camera, Weizlar, Germany) to assess morphological changes and density.

For transmission electron microscopy (TEM), the protocol was adapted from Otegui (2020). Cell pellets were fixed in 2.5% (v/v) glutaraldehyde (ASPEN) in 0.1 M phosphate buffer (pH 7.4) overnight at 4 °C, followed by post-fixation in 1% (w/v) osmium tetroxide (OsO_4_; ASPEN) for 2 h. After a graded ethanol dehydration series, samples were embedded in Spurr’s resin (812 embedding medium, SPI, USA). Ultrathin sections (60–80 nm) were cut using an ultramicrotome (Leica UC7; Leica) with a diamond knife (Ultra 45°; Diatome, Nidau, Switzerland), stained with uranyl acetate and lead citrate (ASPEN), and examined under a transmission electron microscope (Tecnai G2 20 TWIN; FEI, Hillsborough, OR, USA) at an accelerating voltage of 120 kV to observe and capture the ultrastructure of cells.

### Physiological responses

To evaluate physiological responses, malondialdehyde (MDA) content, soluble sugar content, soluble protein content, and the activities of antioxidant enzymes (SOD, CAT, POD) in sugarcane suspension cells were measured. MDA content was determined using the thiobarbituric acid (TBA; Sinopharm Chemical Reagent Co., Ltd.) reaction according to Draper & Hadley (1990). Soluble sugar content was measured by the sulfuric acid–phenol method (DuBois et al., 1956) (phenol; Sinopharm Chemical Reagent Co., Ltd.). Soluble protein content was quantified using the Bradford method (Bradford, 1976) (Coomassie Brilliant Blue; Nanjing Jiancheng Bioengineering Institute, Nanjing, China), with bovine serum albumin (BSA; QASEG, China) as the standard. Superoxide dismutase (SOD) activity was assayed by measuring the inhibition of nitro blue tetrazolium (NBT; MACKLIN) photoreduction (Giannopolitis & Ries, 1977). Catalase (CAT) activity was determined using the H_2_O_2_ disintegration method (Cakmak & Horst, 1991). Peroxidase (POD) activity was measured *via* the guaiacol (MACKLIN) oxidation assay (Srivastava & Dwivedi, 1998). Three biological replicates were performed for each treatment. One-way ANOVA was used to test the differences among the treated samples and the control. The differences were considered significant when the *p*-values were less than 0.05.

### RNA sequencing and transcriptome analysis

Based on the results of cell viability determination and physiological indicators, sugarcane suspension cell samples treated with 0% (as control) and 5% PEG concentrations for 8 h were selected for transcriptome sequencing. To reduce the interference of false—positive signals, three biological replicates were set for each treatment group, namely the drought—treated group (DT1, DT2 and DT3) and the control group (CK1, CK2 and CK3). Total RNA (three µg per sample) was extracted from the six samples using a commercial RNA extraction kit (QIAGEN, Hilden, Germany), and RNA integrity was verified (RIN > 8.0) using an Agilent 2100 Bioanalyzer. Library construction and paired-end (PE150) sequencing were performed on an Illumina Novaseq 6000 platform by Novogene (China). Raw reads were filtered using Trimmomatic to remove adapters and low-quality sequences (Bolger, Lohse & Usadel, 2014). Clean reads were aligned to the sugarcane reference genome using HISAT2, and gene expression levels were quantified as Fragments Per Kilobase Million (FPKM) using StringTie (Pertea et al., 2015). Differentially expressed genes (DEGs) were identified with DESeq2 using the criteria |log_2_(fold change)|≥ 1 and adjusted *p*-value (padj) < 0.05 (Love, Huber & Anders, 2014). Gene Ontology (GO) and Kyoto Encyclopedia of Genes and Genomes (KEGG) enrichment analyses were performed using the clusterProfiler R package (Yu et al., 2012). A protein–protein interaction (PPI) network was predicted using STRING (v11.5) (Szklarczyk et al., 2021) and visualized with Cytoscape (v3.9.1) (Shannon et al., 2003). The statistical power of this experimental design, calculated in RNASeqPower is 0.89. This was achieved using three biological replicates per treatment group with no technical replicates.

### Target gene quantitative reverse transcription polymerase chain reaction validation

To validate the RNA-seq data, nine candidate genes involved in water transport (*PIP1-5*, *TIP3-1*), cell wall modification (*XTH8*, *XTH23*, *CSLD5*), and oxidative defense (*PER25*, *PER56*, *APX1*, *APX2*) were selected for quantitative reverse transcription polymerase chain reaction (qRT-PCR) analysis. Total RNA was extracted as described in ‘RNA Sequencing and Transcriptome Analysis’. First-strand cDNA was synthesized from one µg of total RNA using a reverse transcription kit (Thermo Fisher, USA). Gene-specific primers were designed using Primer 5.0 (Table S1). The sugarcane *GAPDH* and *UBQ* genes were used as internal references (Xu et al., 2026; Huang et al., 2026). qRT-PCR was performed on a StepOnePLUS Real-Time PCR System (Applied Biosystems, Waltham, MA, USA) using ChamQ Universal SYBR qPCR Master Mix (Vazyme, Nanjing, China). The 10 μL reaction mixture contained one μL cDNA, 0.4 μL each of forward and reverse primers (10 μM), five μL SYBR Master Mix, and 3.2 μL ddH_2_O. The thermal cycling conditions were: 95 °C for 10 min, followed by 40 cycles of 95 °C for 15 s and 60 °C for 30 s (fluorescence acquisition). Melting curve analysis was performed from 60 °C to 95 °C. Relative expression levels were calculated using the 2^−^ΔΔCT method.

### Statistical analysis

All experiments followed a completely randomized design with at least three independent biological replicates. Data from physiological assays are presented as mean ± standard deviation (SD). Significant differences between treatments were determined by one-way analysis of variance (ANOVA) followed by Duncan’s multiple range test (*p* < 0.05) using SPSS Statistics software (v26.0; IBM).

### Data availability

The transcriptome sequences and annotation information of *Saccharum* spp. suspension cells under drought stress determined in this study have been uploaded to the National Center for Biotechnology Information (PRJNA1403770) for access by relevant researchers.

### Result

### Drought stress induces phenotypic changes and reduces cell viability in sugarcane suspension cells

**Figure 1:**
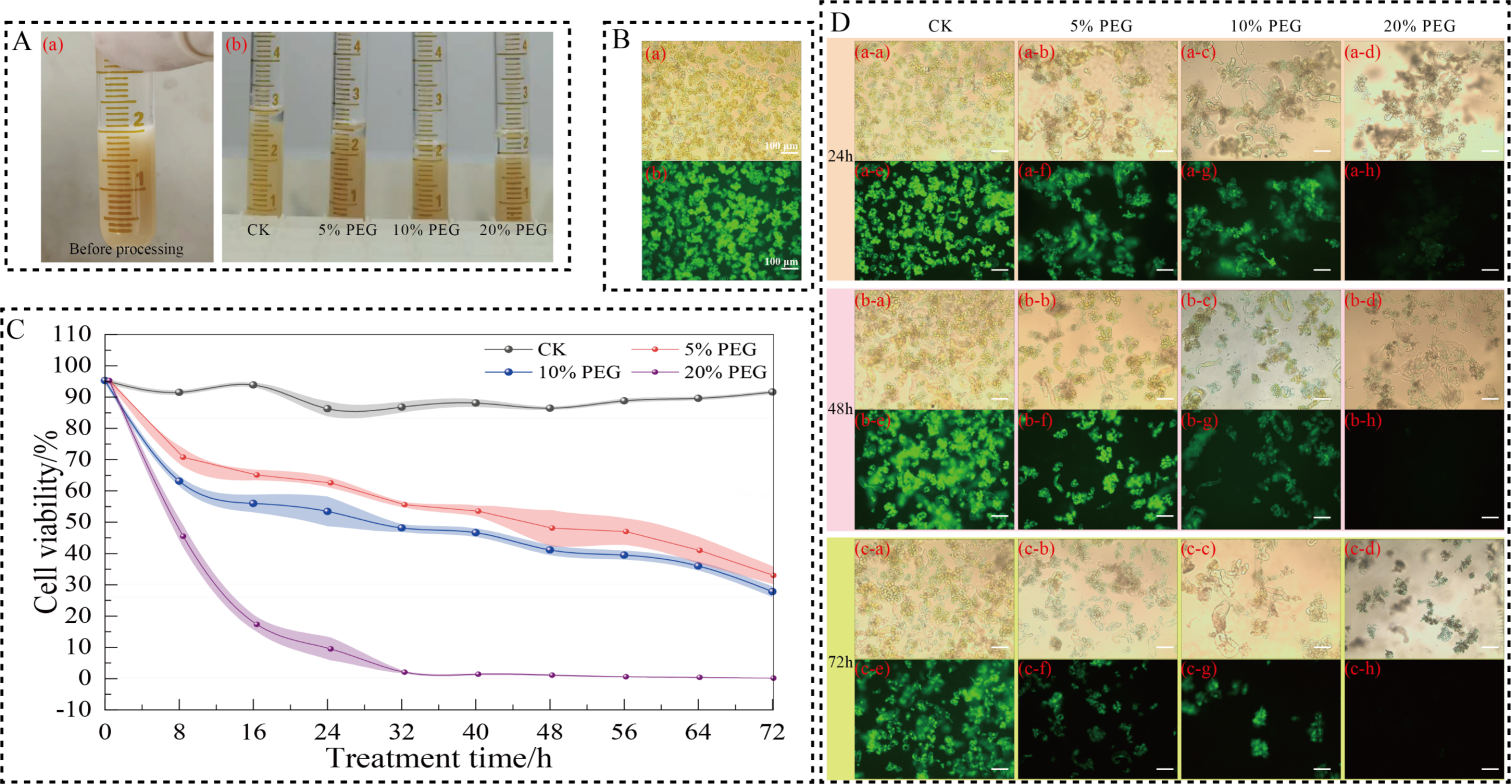
Comparison of growth phenotype and cell viability of sugarcane suspension cells treated with different concentrations of polyethylene glycol. (A) Macroscopic effects of PEG treatments at different concentrations on the growth phenotype of sugarcane suspension cells. (B) Initial material status for sugarcane suspension cell viability detection (bar = 100 µm). (C) Cell viability *versus* time curves after PEG treatment at different concentrations. (D) Fluorescence microscope images of cell viability over time after different concentrations of PEG treatment (bar = 100 µm).

To assess the impact of drought stress on sugarcane suspension cells, the growth phenotype and viability under different concentrations of PEG-6000 were monitored. Macroscopic observation revealed that drought stress caused visible browning of the cells, and the settled cell volume decreased in a PEG concentration-dependent manner (Fig. 1A). Notably, cell volume was reduced by 0.1 mL after treatment with 20% PEG, indicating severe growth inhibition and accelerated cell death.

Quantitative analysis of cell viability using FDA staining confirmed these observations. Sugarcane suspension cells with more than 90% viability were selected as materials (Fig. 1B). The viability of control cells remained consistently high (>90%) throughout the 72-h culture period (Fig. 1C). In contrast, all PEG-treated groups exhibited a significant time-dependent decrease in viability. After 8 h of treatment, cell viability sharply declined to 70.75%, 63.15%, and 45.5% in the 5%, 10%, and 20% PEG groups, respectively (Figs. 1C, 1D). The most severe reduction was observed in the 20% PEG group, where viability plummeted to 1.4% at 40 h, indicating near-complete cell death. Fluorescence microscopy further corroborated these findings, showing a progressive reduction in fluorescent cell count, weakened fluorescence intensity, and an increase in cellular debris with increasing PEG concentration and treatment duration (Fig. 1D). The data indicated that cells retained sufficient viability at 8 h post-treatment to allow for subsequent molecular and physiological analyses.

### Drought stress alters cellular morphology and ultrastructure

To elucidate the cytological impact of drought, we examined cellular morphology and ultrastructure after 8 h of PEG stress. We found that drought stress induced severe, concentration-dependent damage to cellular integrity and organelle structure.

**Figure 2:**
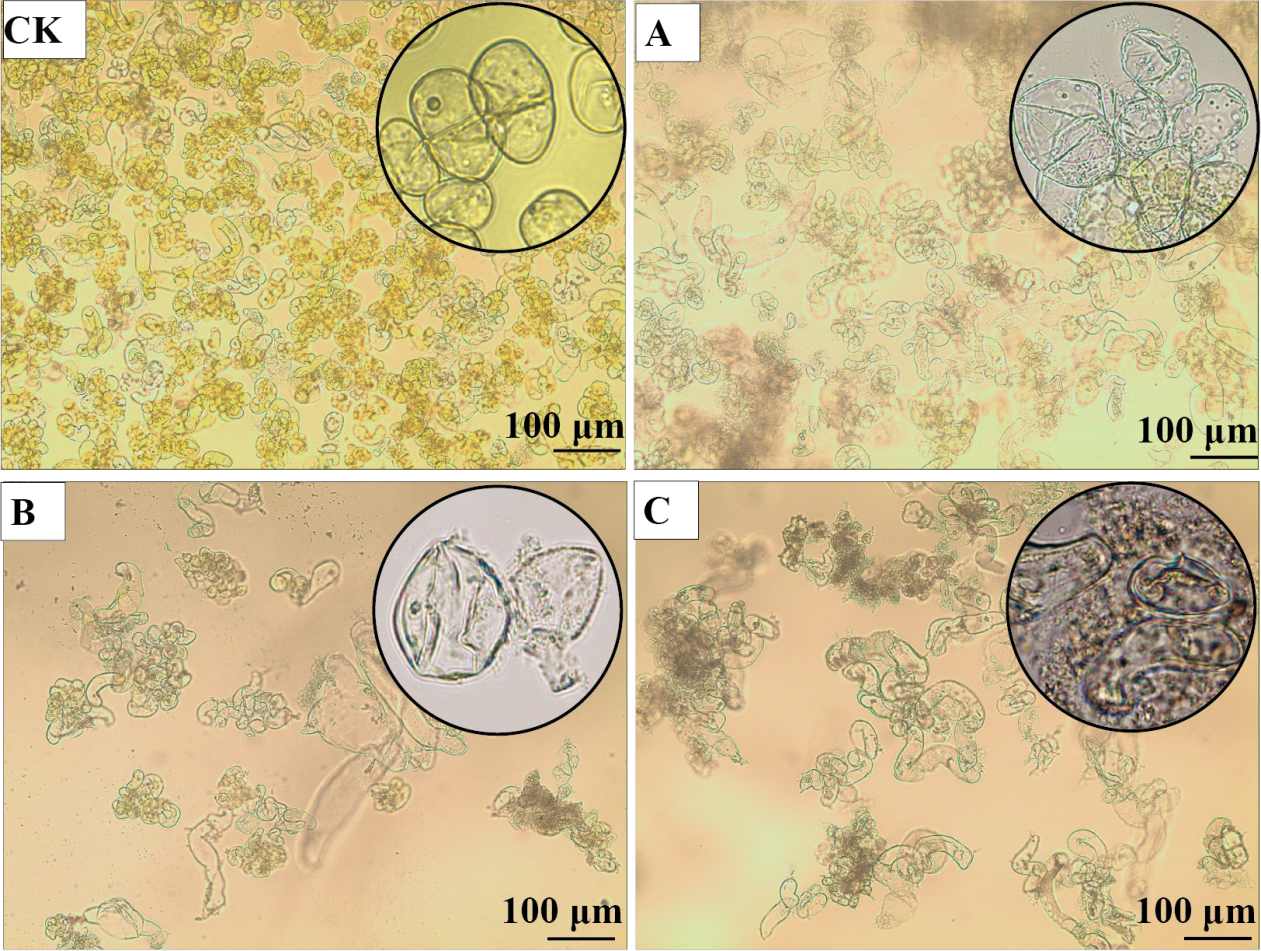
Effects of drought stress with different concentrations of PEG for 8 h on the morphology and density of sugarcane suspension cells (bar = 100 µm). CK, Control group (0% PEG). (A, B, and C) Treatment groups with 5%, 10%, and 20% PEG, respectively. Circles in each panel highlight representative cell morphologies, illustrating progressive changes in cell shape and integrity with increasing drought stress.

Light microscopy revealed a progressive deterioration in cell morphology. Control cells were densely packed, spherical, and exhibited regular morphology (Fig. 2CK). In contrast, PEG treatment led to reduced cell density, increased browning and shrinkage, and loss of structural regularity. These effects were mild under 5% PEG (Fig. 2A), moderate under 10% PEG with blurred cell boundaries (Fig. 2B), and most severe under 20% PEG, which caused severe deformation and abundant cellular debris (Fig. 2C).

**Figure 3:**
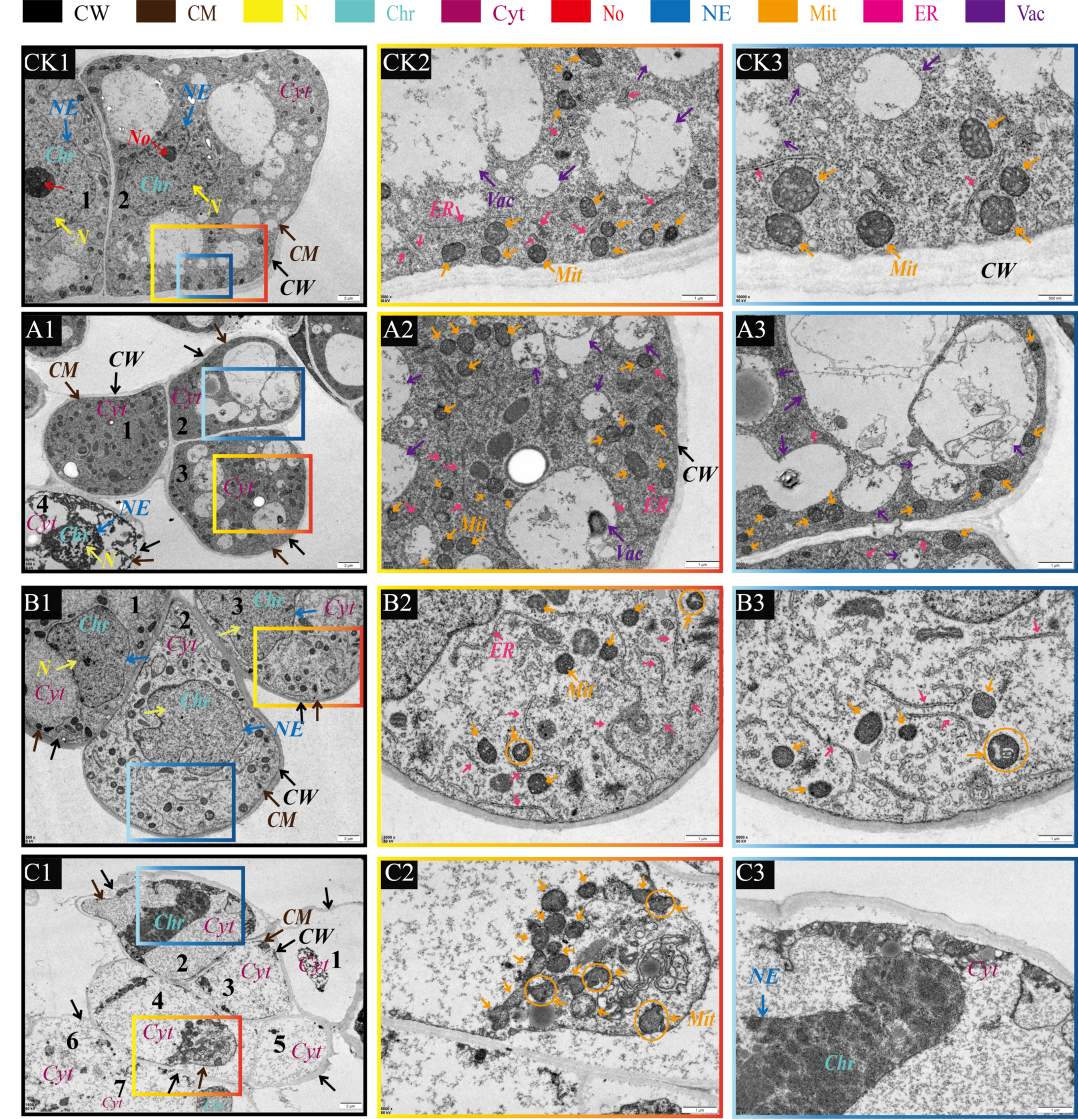
Effects of 8 h drought stress with different concentrations of PEG on Ultrastructure of sugarcane suspension cells. Color legend (top): CW (Cell Wall, black), CM (Cell Membrane, brown), N (Nucleus, yellow), Chr (Chromatin, light blue), Cyt (Cytoplasm, magenta), No (Nucleolus, red), NE (Nuclear Envelope, blue), Mit (Mitochondria, orange), ER (Endoplasmic Reticulum, pink), Vac (Vacuole, purple). Colored arrows match legend colors to indicate structures. Colored boxes in CK1, A1, B1, C1 mark regions that are magnified in corresponding same-row subfigures (*e.g.*, yellow box in CK1 corresponds to magnified view in CK2; blue box in CK1 corresponds to magnified view in CK3; same for A, B, C groups). Bar: 2 µm for CK1, A1, B1, C1; 1 µm for CK2, CK3, A2, A3, B2, B3, C2, C3.

Transmission electron microscopy (TEM) provided detailed evidence of widespread organelle dysfunction. Control cells displayed intact cellular structures, including a continuous cell wall and plasma membrane, well-defined nuclei with clear envelopes and nucleoli, abundant euchromatin, mitochondria with distinct cristae, and elongated endoplasmic reticulum (ER) without dilation (Figs. 3CK1–3CK3). Under 5% PEG stress, while most cells remained intact and healthy, severely affected individuals showed plasma membrane rupture, plasmolysis, extremely low cytoplasmic electron density, and abnormal nuclei with numerous lacunae and condensed chromatin (Figs. 3A1–3A3). Additionally, partial dissolution of the mitochondrial outer membrane was observed, with clear cristae but reduced numbers compared to the control; the tonoplast remained mostly intact with slight dissolution. The 10% PEG treatment induced more uniform damage: multiple cells in the field of view exhibited irregular nuclei, slightly reduced cytoplasmic density, mild edema, and no vacuoles (Figs. 3B1–3B3). The outer membranes of mitochondria showed more severe dissolution; some mitochondria (marked by orange circles) displayed decreased matrix electron density, the appearance of vacuolar structures, and obvious cristae expansion. Additionally, the bilateral membranes of the ER were severely dissolved. The 20% PEG treatment caused catastrophic failure: seven cells were observed in the field of view, showing severe plasma membrane dissolution, significant cytoplasmic loss, and organelle degradation—except for a few cells, no intact nuclei or chromatin were detected (Figs. 3C1–3C3). Mitochondria exhibited obvious swelling (presenting a spherical shape) with a substantial reduction in cristae; some (marked by orange circles) had ruptured outer membranes, with matrix components leaking into the cytoplasm. The nuclear membrane was disrupted and chromatin aggregation was prominent, indicative of programmed cell death (PCD).

### Drought stress triggers oxidative damage and modulates osmotic regulation

**Figure 4:**
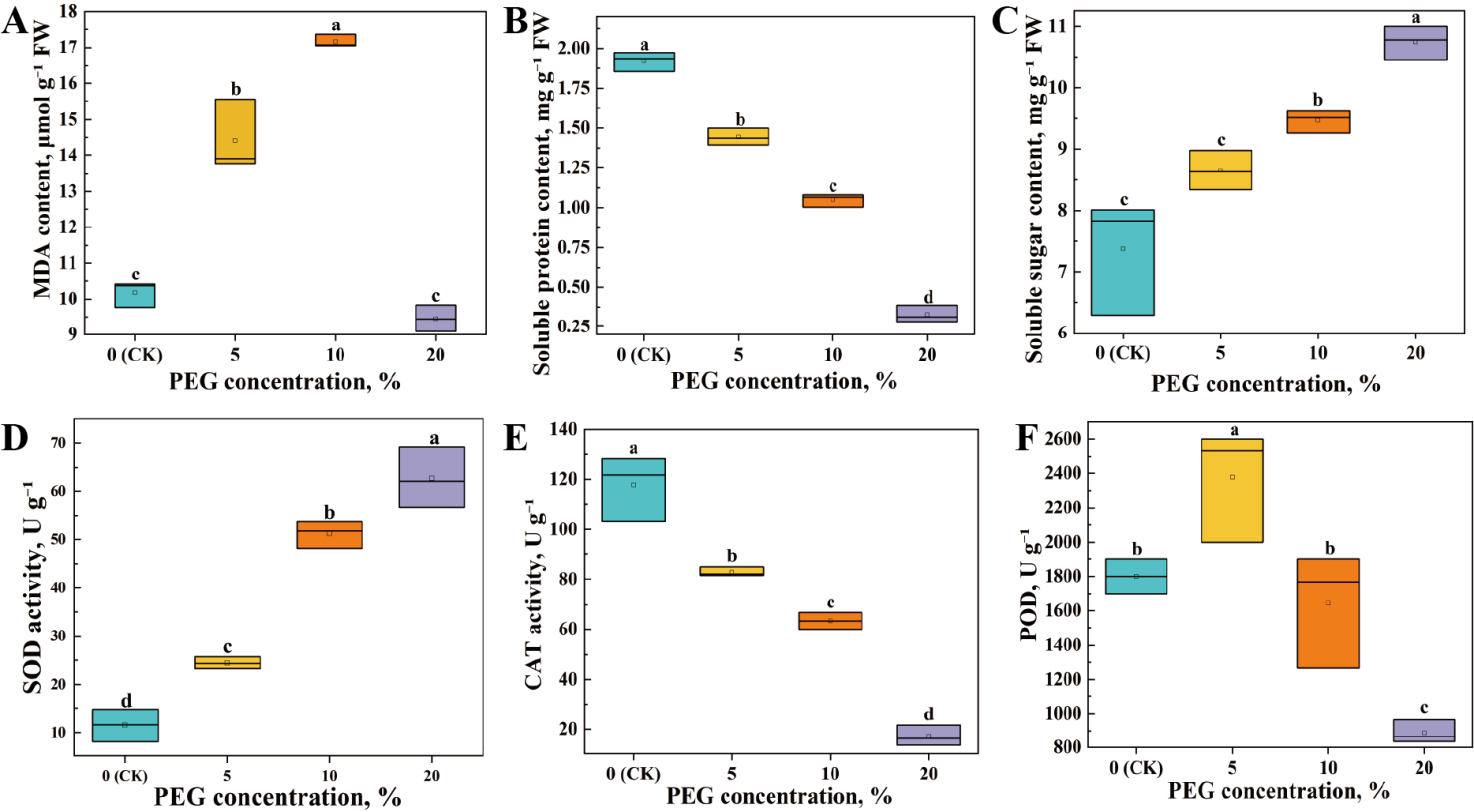
Changes in physiological parameters of sugarcane suspension cells treated with different PEG concentrations for 8 h. (A) Malondialdehyde (MDA) content. (B) Soluble sugar content. (C) Soluble protein content.(D)Superoxide dismutase (SOD) activity. (E) Catalase (CAT) activity. (F) Peroxidase (POD) activity. Box plots show the median (central line), interquartile range (box), and mean value (small square). Different lowercase letters above the boxes denote significant differences among treatments (*p* < 0.05). Error bars represent the standard deviation (SD).

To quantify the physiological response to drought, we measured key stress indicators after 8 h of treatment. Malondialdehyde (MDA) content, a marker of lipid peroxidation and oxidative damage, initially increased with PEG concentration, peaking at 17.16 µmol g^−1^ FW under 10% PEG treatment—a 68.55% increase over the control (*p* < 0.05). Interestingly, MDA content declined under 20% PEG stress (Fig. 4A), likely due to extensive cell death and the collapse of peroxidation substrates.

The content of soluble sugars, key osmolytes, increased markedly with rising PEG concentration, reaching a maximum value of 10.74 mg g^−1^ FW under 20% PEG treatment—a 45.64% increase over the control (Fig. 4B). Conversely, the content of soluble proteins decreased significantly with increasing stress intensity, dropping to a minimum of 0.33 mg g^−1^ FW (82.9% lower than the control) at 20% PEG (Fig. 4C).

### Antioxidant enzyme activities exhibit differential responses to drought

The activities of major antioxidant enzymes showed distinct patterns in response to drought stress (Figs. 4D–4F). Superoxide dismutase (SOD) activity increased consistently with PEG concentration, it nearly doubled from 11.53 U g^−1^ FW (control) to 24.43 U g^−1^ FW at 5% PEG, and reached a maximum of 62.61 U g^−1^ FW at 20% PEG.In contrast, catalase (CAT) activity declined continuously, dropping from 10.57 U g^−1^ FW in the control to 1.47 U g^−1^ FW under 20% PEG treatment, with a reduction of approximately 86%.Peroxidase (POD) activity displayed a biphasic response: it increased to a maximum of 2,377.78 U g^−1^ FW at 5% PEG (significantly above the control) and then decreased to 888.89 U g^−1^ FW at 20% PEG (markedly lower than the 5% PEG peak).

### Transcriptome sequencing analysis

To investigate the molecular mechanism of sugarcane suspension cells in response to drought stress, this study selected sugarcane suspension cell samples treated with 0% and 5% PEG concentrations for 8 h for transcriptome sequencing. Suspension cell samples from drought-treated groups (DT1, DT2, and DT3) and control groups (CK1, CK2, and CK3) were subjected to transcriptome sequencing and a total of 37.64 Gb Clean Data was obtained. The percentage of Q20 bases in all samples was more than 96.35%, and the percentage of Q30 bases was more than 91.08%. In terms of base composition, GC ranged from 53.89% to 54.48%. The mapping rate between samples and reference genes ranged from 76.45% to 78.21%, with an average of 39.36% of reads mapped to unique positions in the genome (Table S2).

### Screening of differentially expressed genes

**Figure 5:**
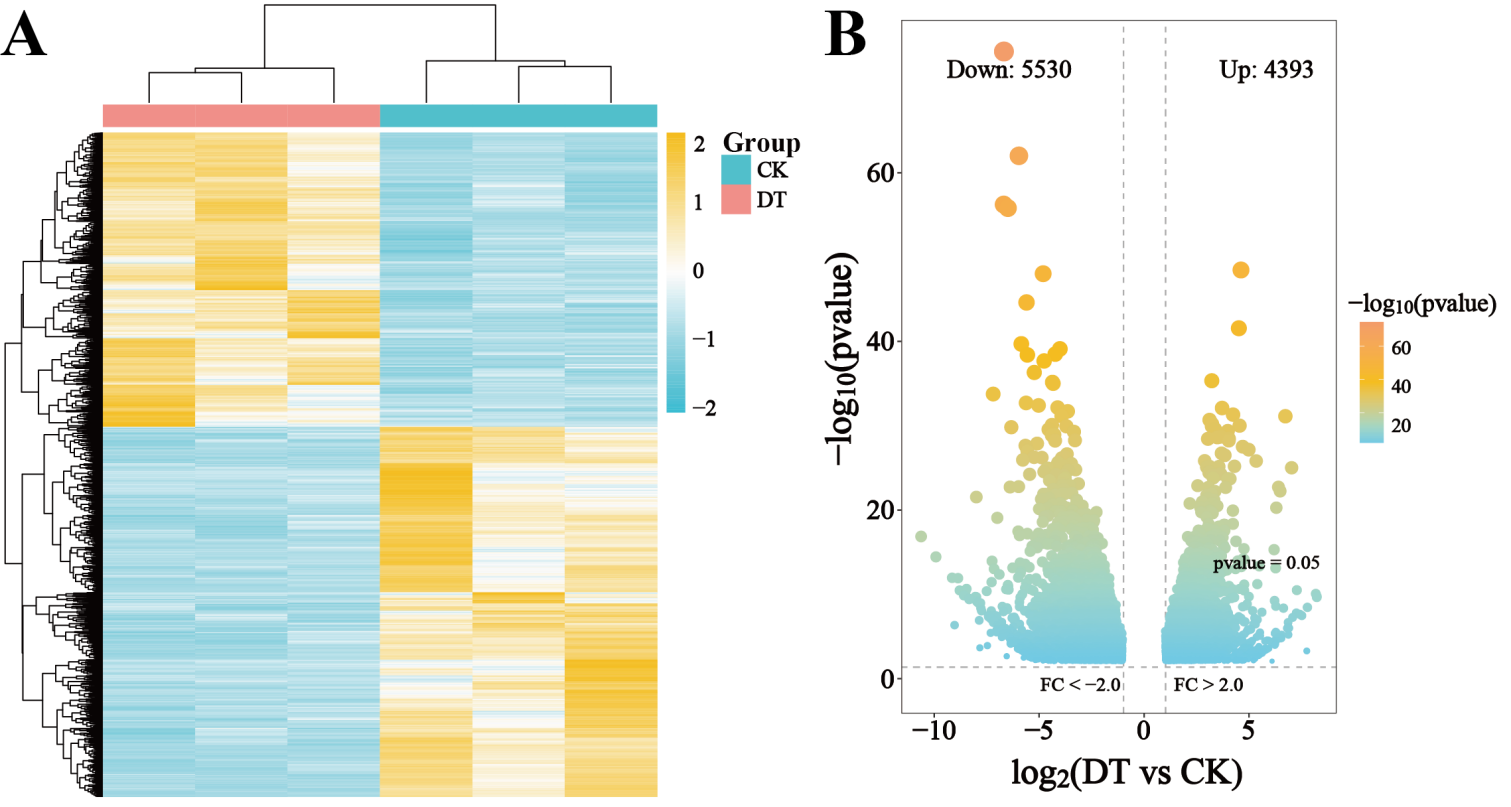
Differential expression analysis of genes in sugarcane suspension cells under drought stress. (A) Heatmap of DEG cluster analysis for DT and CK transcriptomes. The black dendrogram shows hierarchical clustering of samples and genes with similar expression patterns. The color scale represents normalized gene expression levels (yellow/orange = high expression, blue = low expression), demonstrating clear separation between CK and DT groups. (B) Volcano plot of differentially expressed genes. Orange dots indicate up-regulated genes (log_2_FC > 1, *p* < 0.05), blue dots indicate down-regulated genes (log_2_FC < −1, *p* < 0.05), and gray dots indicate non-significant genes.

To explore drought stress-responsivegene expression patterns in sugarcane suspension cells, differential analysis was performed on sequenced genes. Hierarchical clustering of FPKM values showed distinct separation of gene expression between drought-treated (DT) and control (CK) groups (Fig. 5A). Statistical analysis identified 9,923 differentially expressed genes, with 4,393 up-regulated and 5,530 down-regulated (Fig. 5B).

### GO classification of DEGs

GO enrichment analysis was performed on 9,923 DEGs, with 9881 successfully annotated to biological process (BP), cellular component (CC), and molecular function (MF). This analysis yielded 1,326 enriched GO terms in total, including 701 for BP, 182 for CC, and 443 for MF. We focused on the GO terms with the highest number of annotations for up-regulated and down-regulated DEGs, respectively, and further screened the significantly enriched ones for subsequent analysis (Fig. S1).

In BP, up-regulated DEGs were significantly enriched in “cellular amino acid biosynthetic process” (34 genes, GO:0008652), “alpha-amino acid biosynthetic process” (22 genes, GO:0016051), and “carbohydrate biosynthetic process” (21 genes, GO:0016051); down-regulated DEGs were highly enriched in “response to oxidative stress” (86 genes, GO:0006979), “polysaccharide metabolic process” (76 genes, GO:0005976), and “carbohydrate biosynthetic process” (75 genes, GO:0016051).

For CC, up-regulated DEGs were associated with “extracellular region” (23 genes, GO:0005576), “extracellular matrix” (18 genes, GO:0031012), and “extracellular region part” (18 genes, GO:0005886); down-regulated DEGs were linked to “endoplasmic reticulum” (44 genes, GO:0005783) and “extracellular region” (19 genes, GO:0005576).

In MF, “protein heterodimerization activity” (154 genes, GO:0046982) and “copper ion binding” (46 genes, GO:0005507) were prominent among up-regulated DEGs, whereas “glycosyltransferase activity” (75 genes, GO:0046527) and “cellulose synthase activity” (55 genes, GO:0016051) were significantly enriched among down-regulated DEGs.

### KEGG pathway analysis of DEGs

KEGG pathway analysis further revealed that DEGs were significantly enriched in 108 pathways, which were categorized into five functional classes: metabolism (83), genetic information processing (13), environmental information processing (2), cellular processes (4), and biological systems (1) (Fig. S2). The most highly represented pathways included “ABC transporters” (107 DEGs, ID: 02010), “Ribosome biogenesis in eukaryotes” (78 DEGs, ID: 03008), “Motor proteins” (68 DEGs, ID: 04814), “Aminoacyl-tRNA biosynthesis” (49 DEGs, ID: 00970), and “Galactose metabolism” (49 DEGs, ID: 00052).

Metabolic pathway enrichment analysis was performed for up-regulated and down-regulated DEGs based on *P*-values. For the up-regulated DEGs in sugarcane suspension cells under drought stress, the most significantly enriched pathway was ABC transporters, suggesting its potential key regulatory role. Several other metabolic pathways were also markedly enriched, including alpha-linolenic acid metabolism, valine, leucine and isoleucine biosynthesis, and flavonoid biosynthesis. Among genetic information processing pathways, ATP-dependent chromatin remodeling showed high significance. Additional pathways with significant enrichment included ubiquinone and other terpenoid-quinone biosynthesis, sulfur metabolism, and folate biosynthesis. These results indicate that up-regulated DEGs are primarily involved in biological processes such as substance transport, metabolic regulation, and epigenetic modification (Fig. S3A). For the down-regulated DEGs under drought stress, significant enrichment was observed in pathways including motor proteins, phagosome, and various types of N-glycan biosynthesis. Moreover, metabolic and biosynthetic pathways such as pentose and glucuronate interconversions, ribosome biogenesis in eukaryotes, N-glycan biosynthesis, and steroid biosynthesis were also significantly down-regulated (Fig. S3B).

### Protein–protein interaction analysis of DEGs

**Figure 6:**
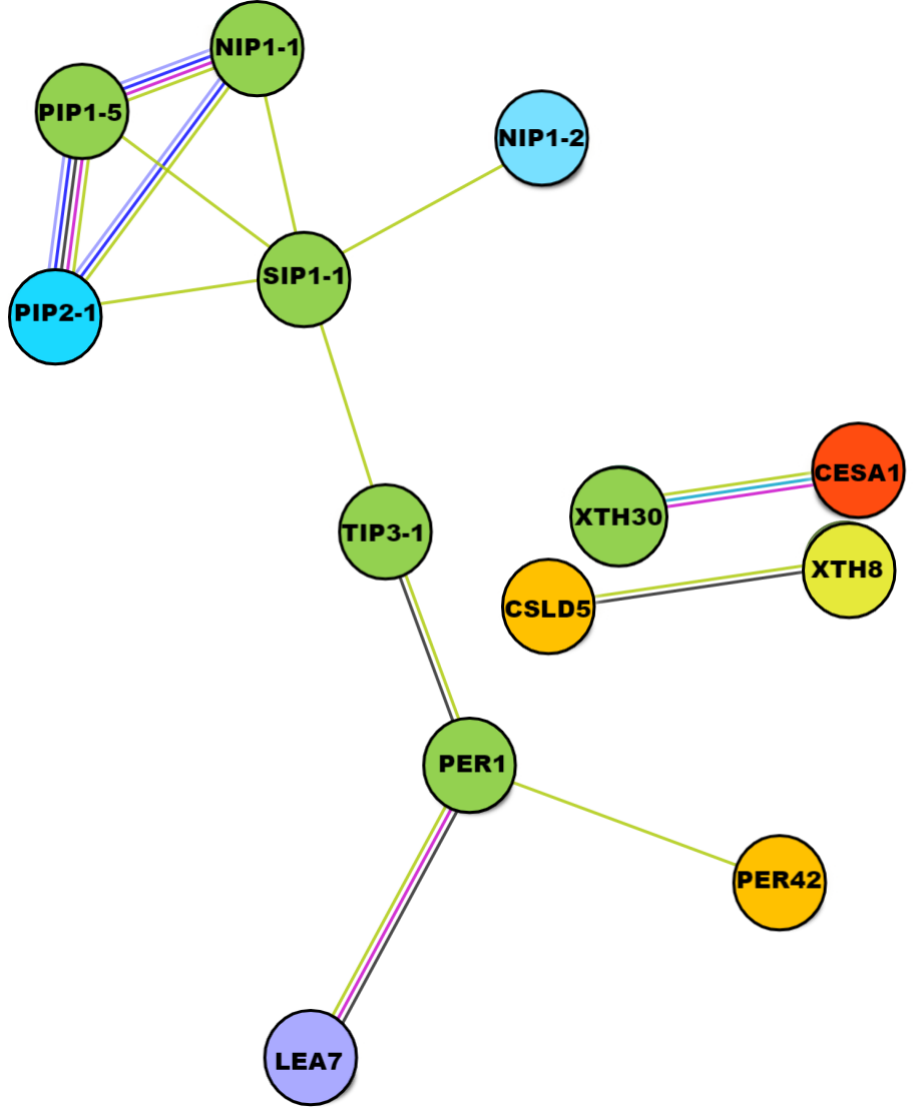
Protein-protein interaction analysis of DEPs.

To understand the interactional networks of drought stress response proteins in sugarcane suspension cells, the DEGs of particular interest were submitted to the STRING database (http://www.string-db.org) with confidence scores higher than 0.40. The protein-protein interaction (PPI) network containing 13 proteins was divided into three independent groups by STRING analysis (Fig. 6). In the core region of PPI network, nine proteins aggregate to form the largest group. Among them, the aquaporin family (*NIP1-1*, *PIP1-5*, *PIP2-1*, *SIP1-1*, *Tip3-1, NIP1-2*), as a key channel for cell membrane water transport, closely interacts with the Peroxidase involved in reactive oxygen species metabolism (*PER1*, *PER42*), and play important roles in reactive oxygen species metabolism, water balance, and the cooperative regulation of oxidative homeostasis in cells under drought stress. Late embryonic development abundant protein (*LEA7*) assists Peroxidase to maintain oxidative metabolism homeostasis by binding water molecules and stabilizing biological macromolecules. In addition, cellulosic synthase (*CESA1*), Xyloglucan endotransglycosylase/hydrolase (*XTH30*, *XTH8*), and cellulosic synthase-like protein (*CSLD5*) are involved in cell wall cellulose synthesis and component remodeling, changes of cellular mechanical stress in response to drought stress.

### Drought stress-related differentially expressed candidate genes in sugarcane suspension cells

To deeply explore the key genes affecting sugarcane suspension cells under drought stress, based on relevant literature, this study integrated differential expression analysis, functional enrichment, and protein–protein interaction (PPI) network for investigation, and screened out 21 candidate genes (Table S3). These genes are categorized into four functional classes: the aquaporin protein family, genes related to cellulose synthesis and modification, peroxidase genes, and genes related to antioxidant and stress response.

The aquaporin (AQPs) family genes (five genes), including *NIP1-1* (ROC_So_ Chr03B0012710), *PIP1-5* (Ctg_00299990), *PIP2-1* (YZ_Ss_Chr05A0010210), *TIP3-1* (YZ_Ss_Chr01A0007450), and *NIP1-2* (YZ_Ss_Chr02A0004430), encode aquaporin proteins, regulate trans - membrane water transport, and maintain cellular water balance under drought. The expression level of some genes (*e.g.*, *PIP2-1*) was significantly down - regulated in the drought group (DT/CK expression ratio: 45.00/304.76, fold change: −2.76); the expression of some genes (*e.g.*, *NIP1-1*) was down - regulated (9.77/79.97, fold change: −3.04).

Genes related to cellulose synthesis and modification include *CESA1* (YZ_Rec_ Chr02A0001630), *XTH8* (YZ_Ss_Chr08A0015770), *XTH23* (ROC_Rec_Chr04B0027150), *XTH30* (ROC_Rec_Chr01A0010140), and *CSLD5* (YZ_So_Chr04B0014250). They are involved in cell wall cellulose synthesis and xyloglucan modification, dynamically adjusting the cell wall structure to adapt to drought stress.

Genes of the peroxidase (PER) family include *PER1* (YZ_Rec_Chr05A0001170), *PER2* (YZ_Rec_Chr05A0049310), *PER3* (ROC_Rec_Chr04B0025980), *PER24* (YZ_Rec_Chr03A0010470), *PER25* (YZ_Rec_Chr02A0002050), *PER42* (ROC_Rec_ Chr04B0000660), *PER50* (Ctg_00130940), *PER56* (YZ_Rec_Chr01A0004770), and *PER70* (YZ_So_Chr01C0034050). They are involved in reactive oxygen species (ROS) metabolism and scavenge excess ROS induced by drought. The expression of most genes (*e.g.*, *PER1*: 27.60/231.94, fold change: −3.07; *PER56*: 0.33/47.43, fold change: −7.00) was down-regulated; the expression of a few genes (*e.g.*, *PER25*: 36.42/8.455, fold change: 2.10) was up-regulated.

There are two antioxidant - related genes: *APX1* (YZ_Rec_Chr01B0046970) and *APX2* (ROC_So_Chr05A0012130), which encode ascorbate peroxidase and enhance intracellular ROS scavenging. The expression level of *APX1* in the drought group (68.50) was much higher than that in the control group (0.91), with a fold change of 6.22; the expression of *APX2* was up - regulated (312.06/120.90, fold change: 1.37).

### Real-time fluorescence quantitative analysis

**Figure 7:**
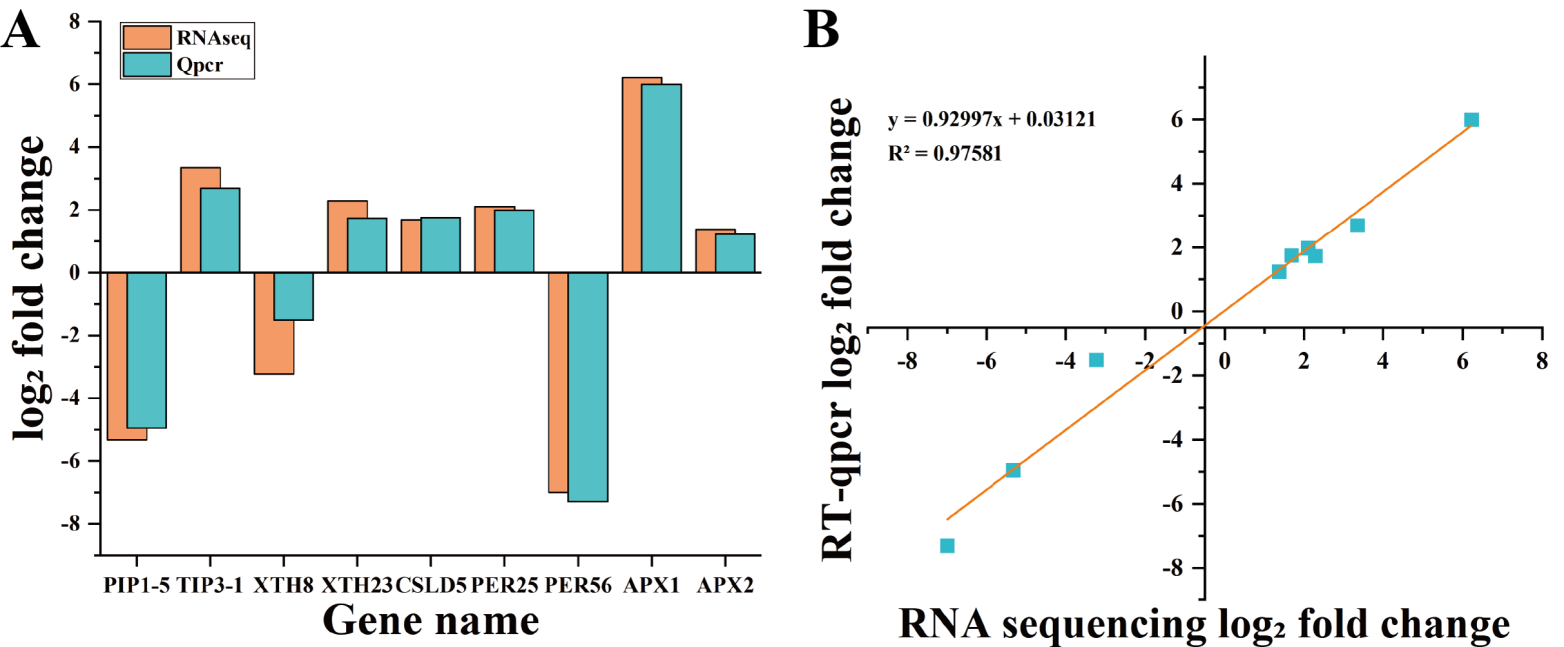
Comparison of RNA-seq and RT-qPCR expression profiles of selected differentially expressed genes (DEGs) in sugarcane suspension cells under drought stress. (A) The abscissa indicates the selected DEGs related to drought stress in sugarcane, and the ordinate indicates the log_2_ fold change of gene expression measured by RNA-seq (orange bars) and RT-qPCR (blue bars). The column height represents the differential expression multiple, and the smaller the height difference between orange and blue bars, the more consistent the expression trend between the two methods. (B) Correlation analysis of log_2_ fold change values from RNA-seq (*x*-axis) and RT-qPCR (*y*-axis). Each blue square represents one DEG. The linear regression equation and *R*^2^ value (*R*^2^ = 0.97581) indicate a high positive correlation between the two techniques, verifying the reliability of the transcriptome sequencing data.

To further validate the reliability of transcriptome sequencing data, qRT-PCR validation was performed on 9 candidate differentially expressed genes (DEGs), including *PIP1-5*, *TIP3-1*, *XTH8*, *XTH23*, *CSLD5*, *PER25*, *PER56*, *APX1*, and *APX2*. As shown in Fig. 7, the expression trends of all selected genes detected by qRT-PCR were basically consistent with the results obtained by transcriptome sequencing. Correlation analysis showed a high positive correlation (*R*^2^ > 0.98) between qRT-PCR data and transcriptome data, which confirmed the reliability and accuracy of transcriptome sequencing results in this study.

## Discussion

### Progressive ultrastructural damage and the initiation of programmed cell death

Our cytological observations reveal a clear dose-dependent effect of drought stress on cellular integrity. The gradual deterioration of cellular structures—from plasma membrane rupture and mitochondrial swelling to nuclear envelope disintegration and chromatin condensation—with increasing PEG concentrations illustrates the progressive nature of drought-induced cellular damage. Notably, the ultrastructural changes observed under severe stress (20% PEG)—including mitochondrial outer membrane rupture, cristae fragmentation, and nuclear condensation—closely resemble the hallmark features of PCD previously reported in other plant species (Doronina & Lazareva, 2021; Daneva et al., 2016). This suggests that drought stress may activate conserved PCD pathways in sugarcane cells when damage exceeds a critical threshold. The preservation of structural integrity under mild stress (5% PEG) and its complete breakdown under severe stress highlight the critical balance between adaptive responses and irreversible damage, providing a cytological basis for understanding drought tolerance limits.

### Physiological adaptation: oxidative balance and osmotic adjustment

Physiologically, our results demonstrate that sugarcane cells employ a multifaceted strategy to cope with drought stress. The accumulation of soluble sugars serves as a key osmotic adjustment mechanism, helping to maintain cellular water potential and stabilize macromolecules under dehydration conditions (Fu et al., 2016) .This accumulation is likely driven by the drought-induced activation of genes involved in carbohydrate metabolism and polysaccharide breakdown, rather than being merely a passive consequence of metabolic disruption.Conversely, the significant decline in soluble protein content suggests either enhanced protein degradation or inhibited synthesis, possibly reflecting a metabolic shift that prioritizes stress-responsive proteins over housekeeping functions (Hochholdinger et al., 2005).This pattern of increased soluble sugars alongside decreased soluble proteins (Figs. 4B, 4C) is consistent with the findings in peat moss (Liu et al., 2026), suggesting that this is a common physiological response to drought stress. In contrast, both soluble sugar and soluble protein contents were observed to increase significantly in sugarcane tiller seedlings under drought stress (Tang et al., 2023). This discrepancy may be attributed to differences in experimental materials (suspension cells *vs.* whole plants), stress intensity, or cultivar-specific responses.

Superoxide dismutase (SOD) serves as the first line of defense, catalyzing the dismutation of superoxide radicals (O_2_^−^) into hydrogen peroxide (H_2_O_2_) and oxygen (Raza et al., 2025). Catalase (CAT) is responsible for converting H_2_O_2_ into water and oxygen, playing a crucial role in maintaining cellular redox homeostasis (Kumari, Gangwar & Prasad, 2024). Peroxidase (POD) also scavenges H_2_O_2_ but with a higher affinity and participates in additional processes such as cell wall lignification and auxin metabolism (Yao et al., 2017). In the present study, SOD activity increased continuously with increasing PEG concentration, indicating sustained generation of superoxide radicals and continuous activation of this first-line defense. CAT activity gradually decreased as stress intensified, which may be attributed to enzyme inactivation by excessive H_2_O_2_ or suppression of its synthesis under severe stress. POD activity showed a trend of first increasing then decreasing, suggesting its active recruitment during early adaptation followed by system failure under extreme stress. These patterns are consistent with previous findings that antioxidant enzyme responses vary across species and experimental systems. A “first increase then decrease” trend in SOD, POD, and CAT activities was observed in PEG-stressed passion fruit seedlings (Qi et al., 2023). In contrast, significantly elevated CAT, SOD, and POD activities were reported in sugarcane cultivar CP48-103 under drought stress (Bavai et al., 2025). These differences indicate that the response of the plant antioxidant enzyme system to drought stress is not a uniform model but rather a complex process regulated by multiple factors, including genetic characteristics, stress intensity, and cellular physiological status. The observed lipid peroxidation (MDA accumulation) pattern, peaking at 10% PEG before declining at 20% PEG, further supports the notion that oxidative damage escalates until cellular antioxidant capacity is overwhelmed, eventually leading to the collapse of peroxidation substrates due to extensive cell death.

### Transcriptional reprogramming and core drought - responsive genes

Our transcriptome analysis identified 9,923 differentially expressed genes under drought stress, which reveals extensive transcriptional reprogramming. Notably, these DEGs are enriched in processes such as “cellular amino acid biosynthetic process” (upregulated) and “response to oxidative stress” (downregulated). The upregulation of amino acid biosynthesis pathways (Fig. S3A) alongside decreased soluble protein content (Fig. 4C) suggests a strategic reallocation of cellular resources under drought stress: amino acids are preferentially channeled towards the synthesis of stress-protective proteins or accumulated as osmoprotectants (*e.g.*, proline), rather than being used for general protein synthesis (Hildebrandt, 2018). The enrichment of DEGs in processes such as “cellular amino acid biosynthetic process” (up - regulated) and “response to oxidative stress” (down - regulated) suggests a strategic prioritization of metabolic pathways during drought adaptation. Based on relevant literature, we identified 21 candidate drought-responsive genes by integrating differential expression analysis, functional enrichment, and protein-protein interaction (PPI) network analysis. The coordinated expression of aquaporins (*e.g.*, *TIP3-1*, *NIP1-1*), cell wall modifiers (*e.g.*, *XTH23, CESA1*), peroxidases (*e.g.*, *PER25, PER1*), and antioxidant enzymes (*e.g.*, *APX1*) reveals a sophisticated network that simultaneously regulates water transport, cellular structure, and oxidative homeostasis. Particularly noteworthy is the strong up - regulation of *APX1* (6.22-fold), which encodes a key enzyme in the ascorbate-glutathione cycle for H_2_O_2_ detoxification (Li & Xiao, 2025), highlighting its potential as a master regulator of drought-induced oxidative stress in sugarcane.

### Integrated multi-omics analysis reveals the cellular drought response network in sugarcane

**Figure 8:**
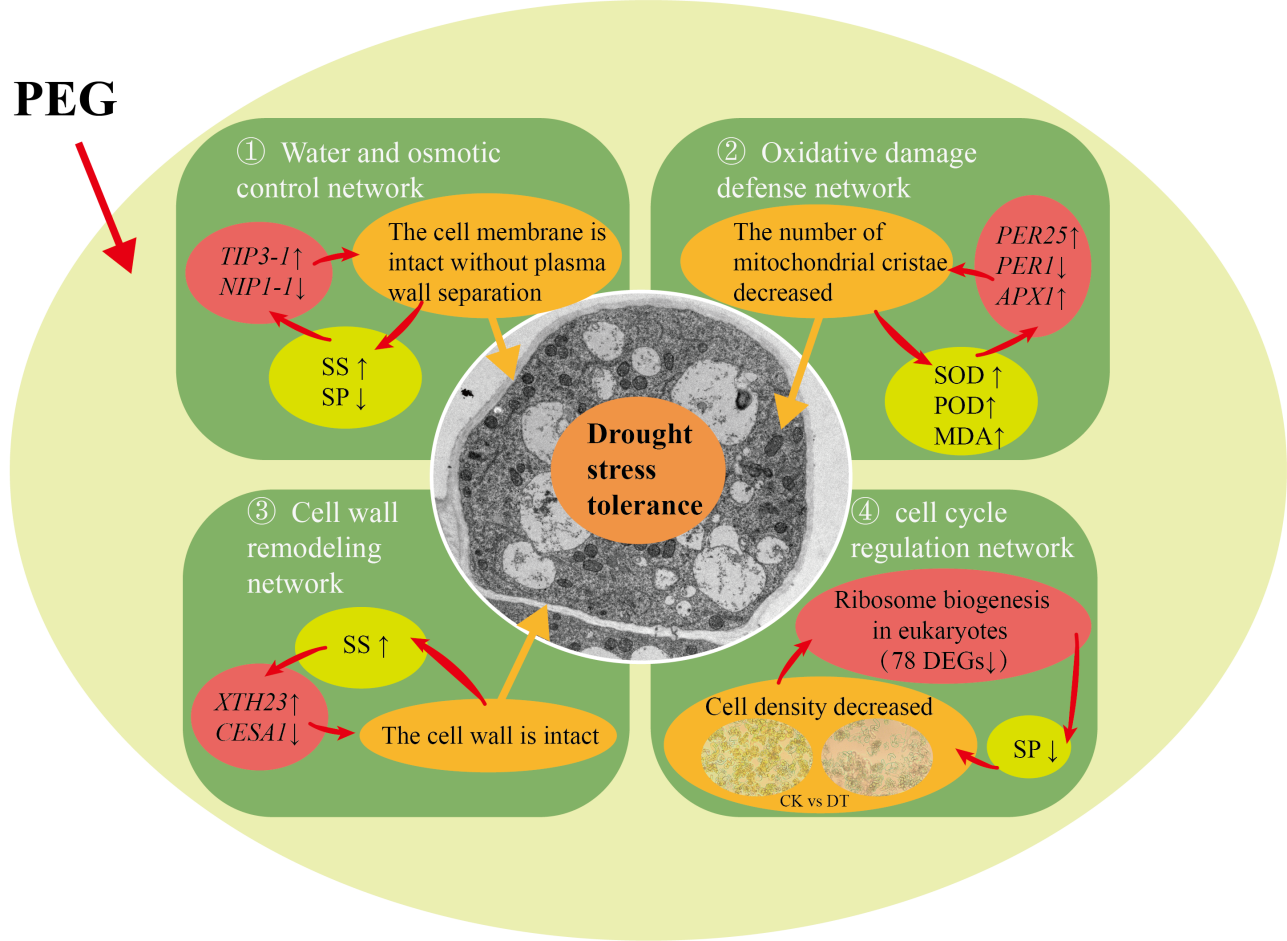
Multi-omics integrated response mechanism of sugarcane suspension cells under 5% PEG - simulated drought stress.

From morphological changes, ultrastructural damage, and physiological responses to complex transcriptional reprogramming, the multiple responses of sugarcane suspension cells act synergistically to jointly form an early stress resistance response system, which primarily involves four core networks: water and osmotic regulation network, oxidative damage defense network, cell wall remodeling network, and cell cycle regulation network (Fig. 8).

In the water and osmotic control network,sugarcane suspension cells treated with 5% PEG maintained almost intact morphology without plasmolysis. This phenomenon was highly consistent with the change of soluble sugar content—its content increased by 17.26% compared with the control (CK), as a key osmotic regulator, soluble sugar helps maintain intracellular water balance by reducing cellular water potential. Meanwhile, the aquaporin (AQPs) family presented distinct differential expression characteristics: *TIP3-1* (YZ_Ss_Chr01A0007450) expression was upregulated, and *NIP1-1* (ROC_So_Chr03B0012710) expression was downregulated. These findings suggest that aquaporins are able to maintain cellular basic turgor by regulating intracellular water distribution (Ndayambaza et al., 2024). Further functional enrichment analysis showed that AQP-related genes were significantly enriched in the“Extracellular region” functional items, and KEGG pathway analysis linked it to the ATP-binding cassette transporter family pathway, which involves 107 differentially expressed genes (DEGs), this result further supports the conclusion that water and osmolytes maintain the osmotic balance of cells through a cooperative transmembrane transport mechanism. In addition, the soluble protein content decreased by 24.83% compared with the control, which may be related to the inhibition of the eukaryotic ribosome biogenesis pathway.

In the oxidative damage defense network, partial sugarcane suspension cells exhibited mild damage to their submicroscopic structures after 5% PEG treatment, specifically manifested as a reduction in the number of mitochondrial cristae and partial dissolution of the endoplasmic reticulum membrane, while the overall structure remained free from severe disintegration. At the physiological level, this treatment induced mild oxidative stress in the cells, with specific manifestations as follows: the activity of superoxide dismutase (SOD) increased by 111.88% compared with the control, the activity of peroxidase (POD) reached its peak, the content of malondialdehyde (MDA) began to rise, and the process of membrane lipid peroxidation was initiated. At the molecular level, the aforementioned oxidative stress and submicroscopic structural changes might be closely related to the differential expression of the peroxidase (PER) family—*PER25* (YZ_rec_chr02a0002050) was upregulated, whereas most PER family genes such as PER1 were downregulated; meanwhile, *APX1* (YZ_rec_chr01b0046970) was significantly upregulated, which could assist in reactive oxygen species (ROS) scavenging by enhancing ascorbate metabolism (Li & Xiao, 2025). Further GO enrichment analysis showed that 86 DEGs were enriched in the “response to oxidative stress” functional term and exhibited a downregulated trend. This result indicates that sugarcane suspension cells can cope with oxidative damage induced by drought stress by regulating the expression of antioxidant-related genes.

In the cell wall remodeling network, the cell walls of sugarcane suspension cells remained basically intact after treatment with 5% PEG. At the physiological level, the accumulation of soluble sugars in the carbon metabolism process was associated with the “carbohydrate biosynthetic process” in the GO pathway—21 differentially expressed genes (DEGs) were upregulated in this pathway, which could provide carbon sources for cell wall synthesis and osmotic regulation. Meanwhile, the expression of genes involved in dynamic cell wall regulation showed specific changes: *XTH23* (ROC_rec_chr04b0027150) was upregulated (fold change: 2.28) to promote xyloglucan modification (Xu et al., 2020), while *CESA1* (YZ_rec_chr02a0001630) was slightly downregulated, enabling adaptation to osmotic stress by reconstructing the mechanical properties of the cell wall (Li et al., 2023).

In the cell cycle regulation network, after 5% PEG treatment, the cell growth volume decreased and the cell viability declined by approximately 29.25% compared with the initial viability. Optical microscopy observations revealed a reduction in cell density, indicating inhibited cell growth and weakened proliferation capacity. KEGG pathway analysis showed that the downregulated differentially expressed genes (DEGs) were enriched in the “Ribosome biogenesis in eukaryotes” pathway (78 DEGs in total), which is directly associated with protein synthesis. The inhibition of this pathway may reduce the intracellular protein synthesis efficiency, and this speculation is highly consistent with the experimental result observed in this study that the soluble protein content decreased by 24.83%. These results suggest that under drought stress, sugarcane cells can adaptively regulate the cell cycle by affecting the synthesis of structural proteins and regulatory proteins required for the cell cycle progression.

## Conclusion

This study provides a comprehensive multi-omics analysis of the drought stress response in sugarcane suspension cells, offering new insights at the cellular level. The main findings are as follows: (1) Drought stress led to a gradual decline in cell viability, caused damage to ultrastructure from the cell membrane to organelles, and under severe stress, further triggered markers of PCD; (2) physiological adaptation was achieved through dynamic regulation of osmolyte accumulation (soluble sugars, soluble proteins), oxidative damage markers (MDA), and differential antioxidant enzyme responses (SOD, CAT, POD); (3) transcriptomic reprogramming revealed 21 core candidate genes involved in water transport (*e.g.*, TIP3-1, NIP1-1), cell wall remodeling (*e.g.*, XTH23, CESA1), and oxidative defense (*e.g.*, APX1, PER family); (4) the proposed network of “water regulation–oxidative defense–cell wall remodeling–cell cycle regulation” offers an integrative model for understanding drought tolerance in sugarcane. The advantages of the suspension cell system were demonstrated: uniform, controllable, and reproducible responses were obtained, circumventing the complexity inherent in whole-plant studies.However, this study has limitations. First, *in vitro* suspension cells combined with PEG-simulated drought cannot fully replicate the complexity of field soil drought or plant–soil–microbe interactions. Second, omics and physiological analyses based on a single 8-h time point may miss later adaptation or recovery mechanisms. Third, there is a lack of direct functional validation of genes, such as overexpression or knockout of candidate genes. Therefore, future research could utilize genetic engineering techniques (*e.g.*, CRISPR-Cas9 gene editing, overexpression vectors) to validate the functions of core genes such as APX1 and XTH23, and conduct multi-time-point, multi-omics dynamic analyses to enhance the temporal understanding of the regulatory network, thereby providing a theoretical basis for future studies and promoting the integration of cellular-level mechanisms with whole-plant research.

## Supplemental Information

10.7717/peerj.21396/supp-1 Supplemental Information 1 GO functional classification analysis of differentially expressed genes (A) GO classification of up-regulated differentially expressed genes. (B) GO classification of down-regulated differentially expressed genes.

10.7717/peerj.21396/supp-2 Supplemental Information 2 KEGG enrichment pathway of differentially expressed genes

10.7717/peerj.21396/supp-3 Supplemental Information 3 KEGG pathway enrichment analysis of differentially expressed genes (A) KEGG pathway enrichment results for up - regulated DEGs. (B) KEGG pathway enrichment results for down - regulated DEGs.

10.7717/peerj.21396/supp-4 Supplemental Information 4 Real-time quantitative fluorescence gene primer sequences

10.7717/peerj.21396/supp-5 Supplemental Information 5 Statistical table of sample sequencing data evaluation

10.7717/peerj.21396/supp-6 Supplemental Information 6 Drought-related differentially expressed 21 candidate genes in sugarcane suspension cells

10.7717/peerj.21396/supp-7 Supplemental Information 7 Raw Data

10.7717/peerj.21396/supp-8 Supplemental Information 8 Computer Code and Data Analysis

10.7717/peerj.21396/supp-9 Supplemental Information 9 Ultrastructure Images

10.7717/peerj.21396/supp-10 Supplemental Information 10 Cell Vitality Fluorescence Images

10.7717/peerj.21396/supp-11 Supplemental Information 11 Cell Morphology Images

## Additional Information and Declarations

### Data Availability

The following information was supplied regarding data availability: The raw data is available in the Supplemental Files. The sequences are available at NCBI: PRJNA1403770
