# Supplementary figures and images for: A cellular view of drought adaptation in sugarcane: multi-omics integration reveals a quadruple module network linking water regulation, oxidative defense, cell wall remodeling, and cell cycle regulation
Source: PeerJ. 2026 Jun 17;14:e21396. doi: 10.7717/peerj.21396 (PMC13282945; doi:10.7717/peerj.21396)

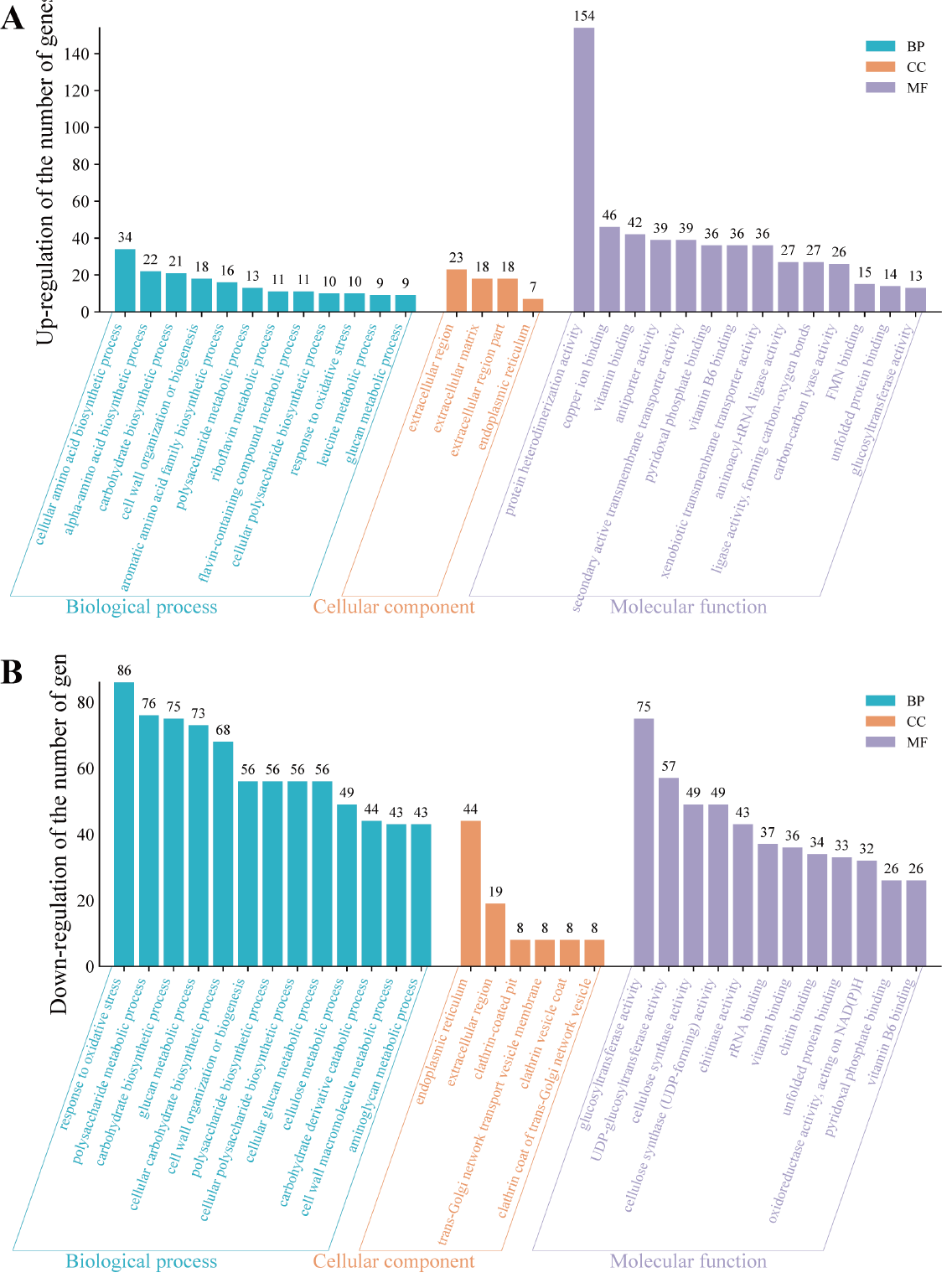

Supplement: Supplemental Information 1 — (A) GO classification of up-regulated differentially expressed genes. (B) GO classification of down-regulated differentially expressed genes. [file peerj-14-21396-s001.png]

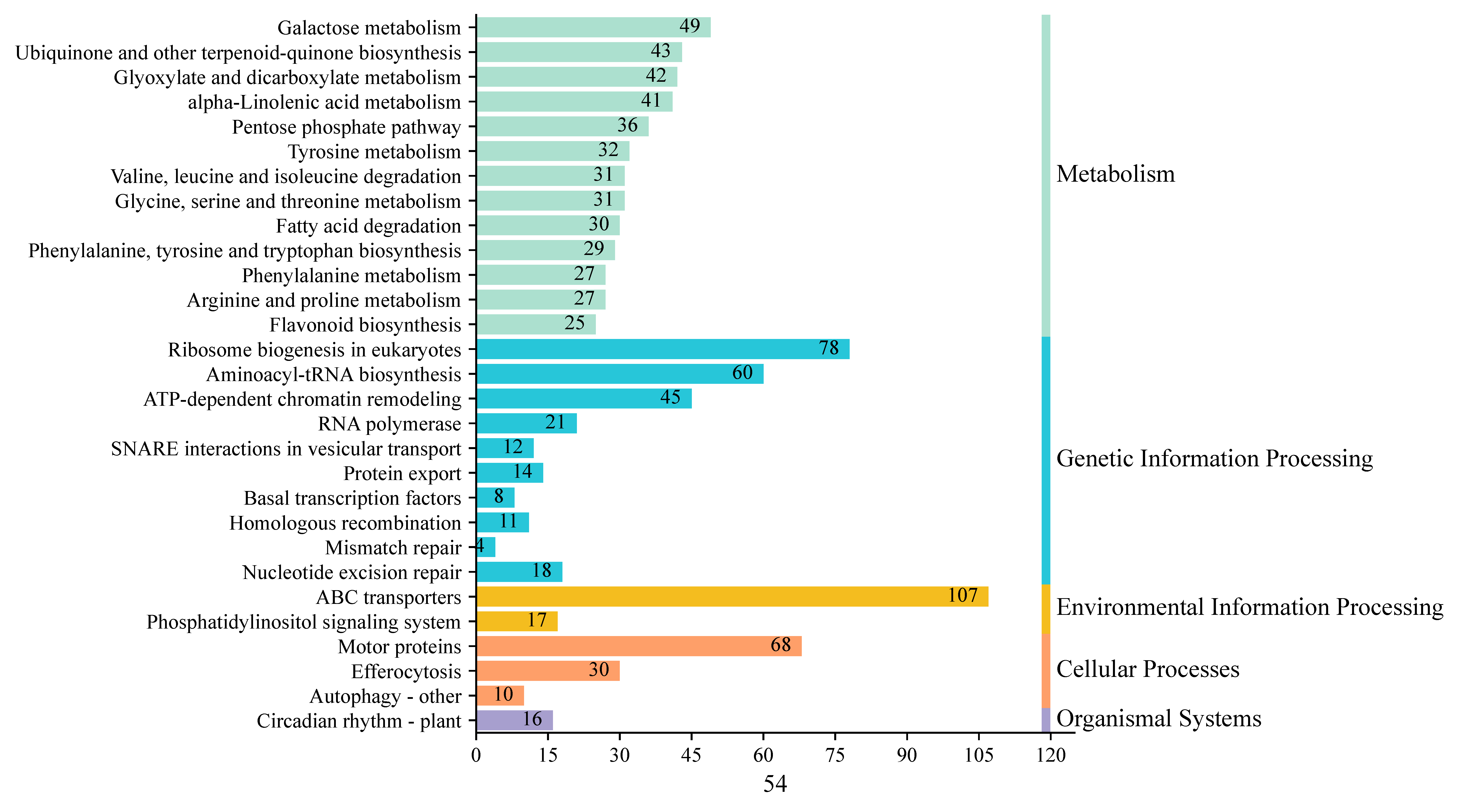

Supplement: Supplemental Information 2 [file peerj-14-21396-s002.png]

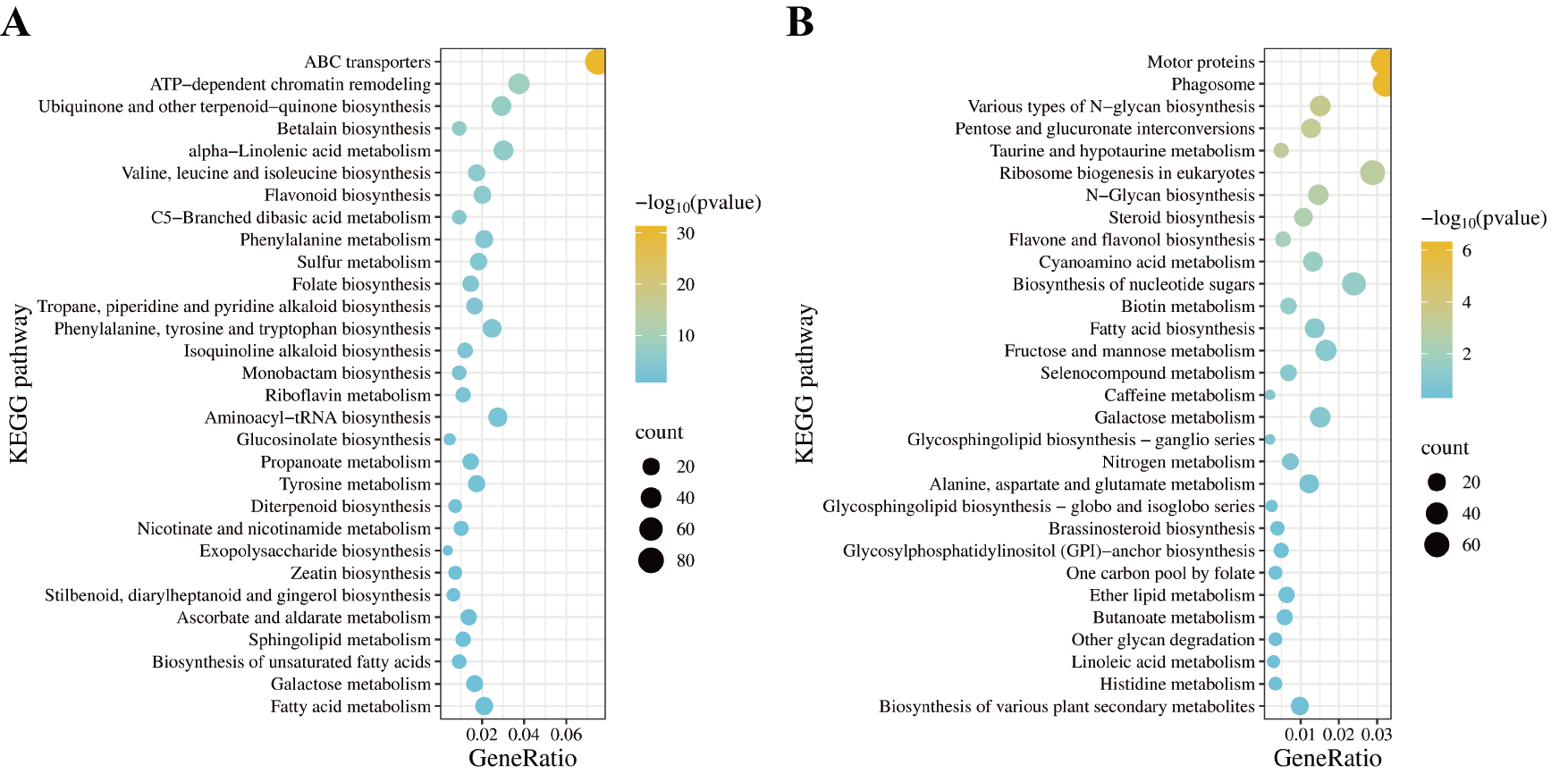

Supplement: Supplemental Information 3 — (A) KEGG pathway enrichment results for up - regulated DEGs. (B) KEGG pathway enrichment results for down - regulated DEGs. [file peerj-14-21396-s003.png]

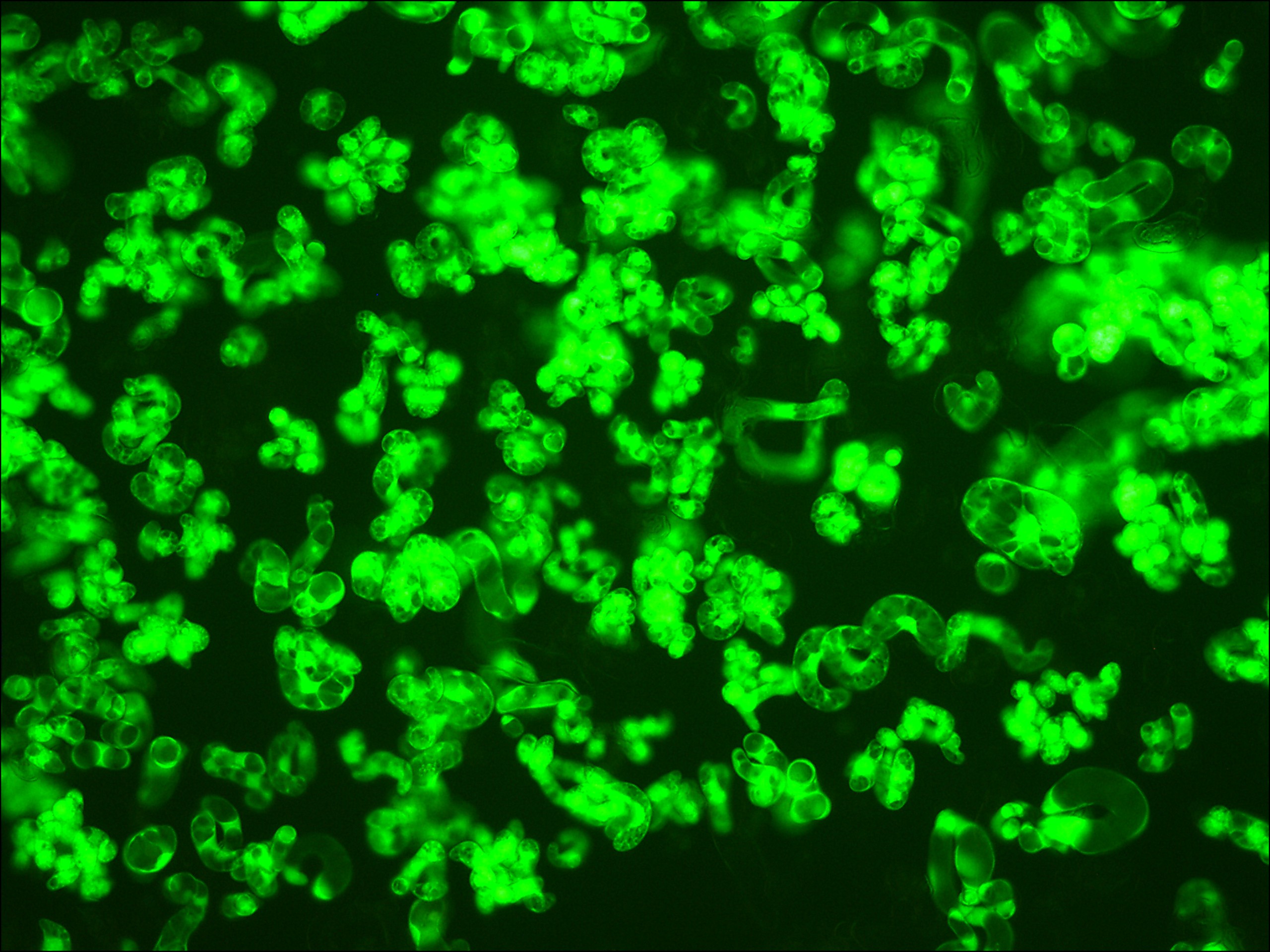

Supplement: Supplemental Information 10 [file peerj-14-21396-s010.zip › Supplementary File for Cell Vitality Fluorescence Images/0-24 Dark.jpg]

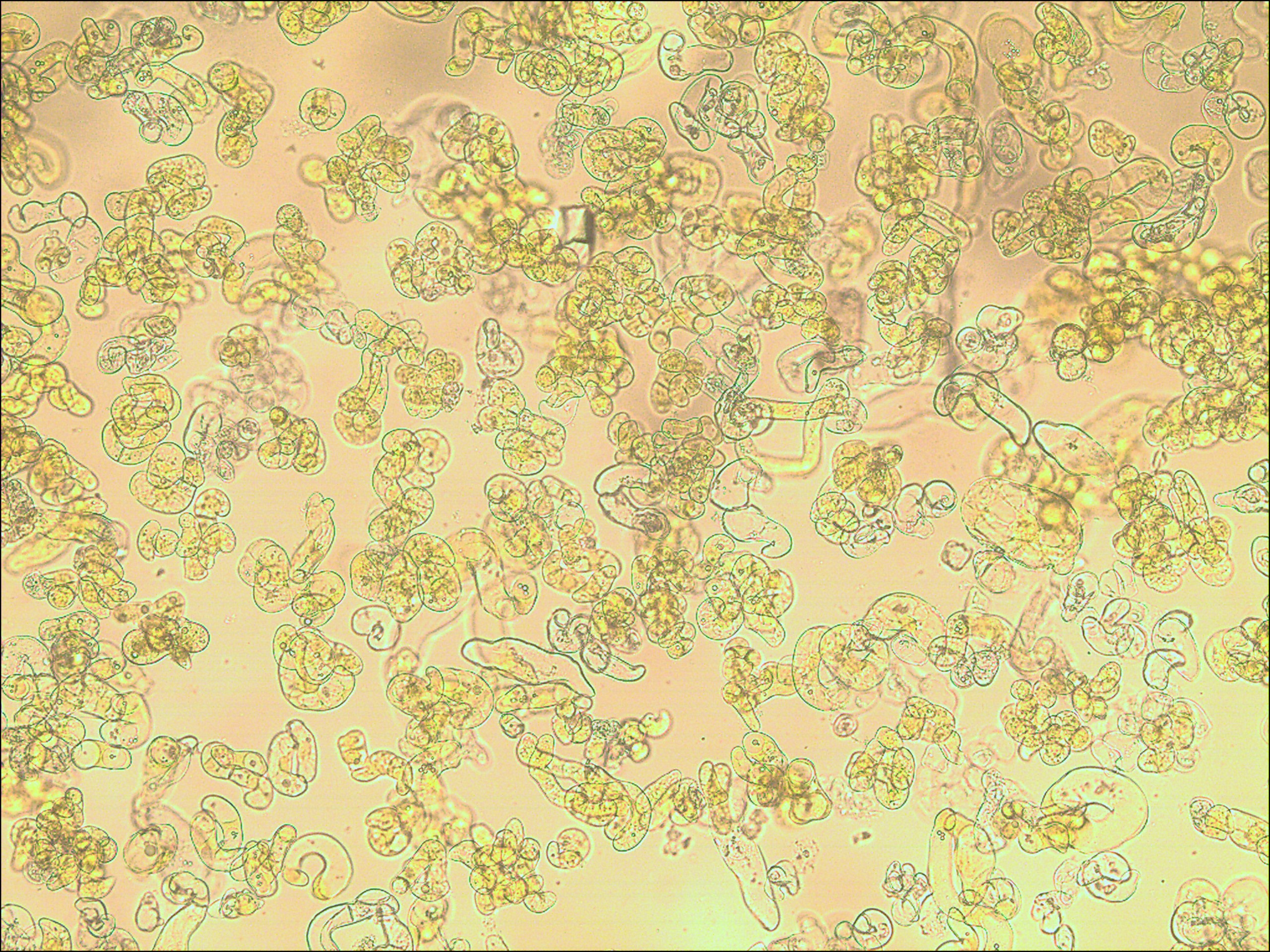

Supplement: Supplemental Information 10 [file peerj-14-21396-s010.zip › Supplementary File for Cell Vitality Fluorescence Images/0-24h Light.jpg]

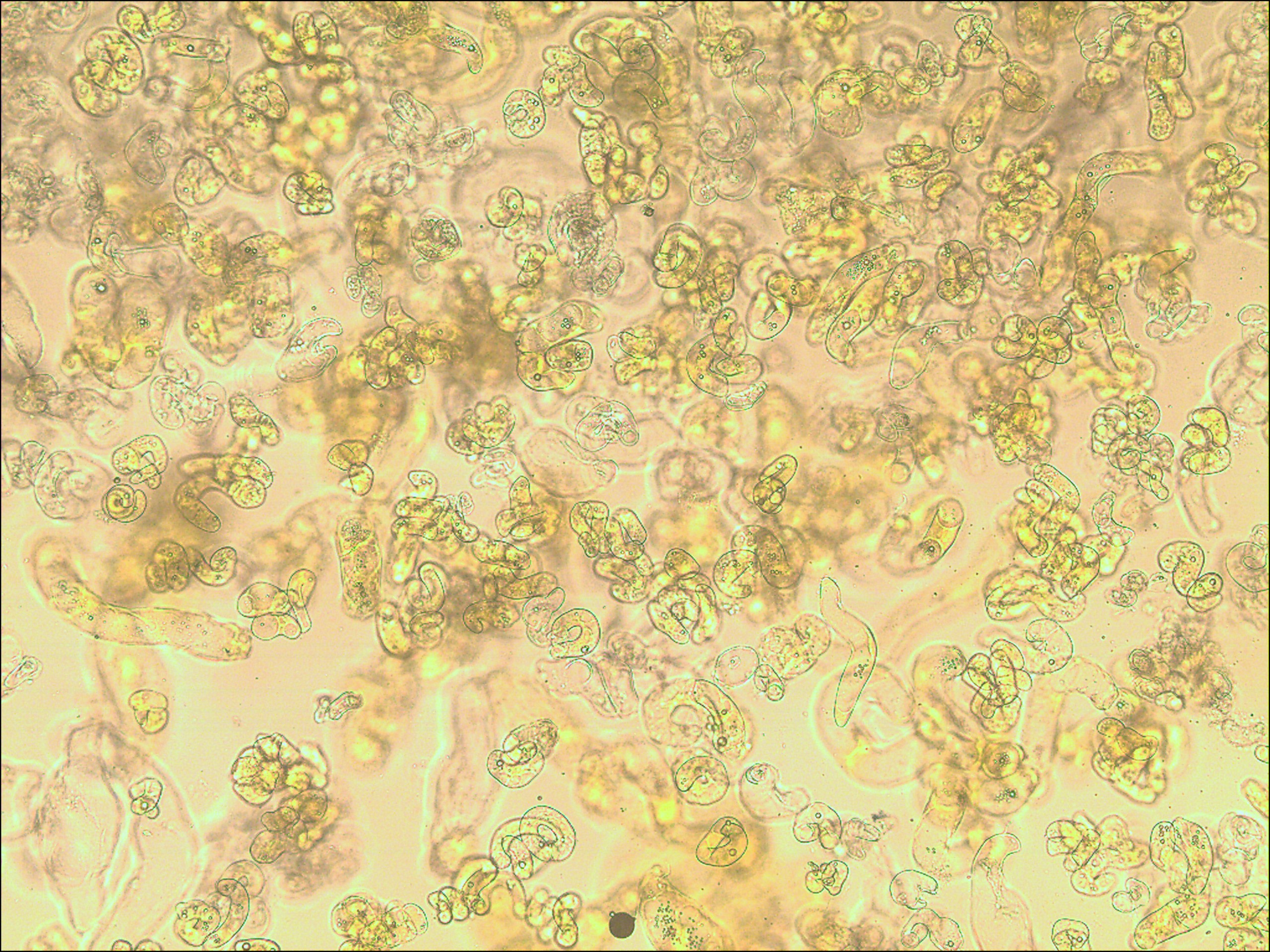

Supplement: Supplemental Information 10 [file peerj-14-21396-s010.zip › Supplementary File for Cell Vitality Fluorescence Images/0-48 Light.jpg]

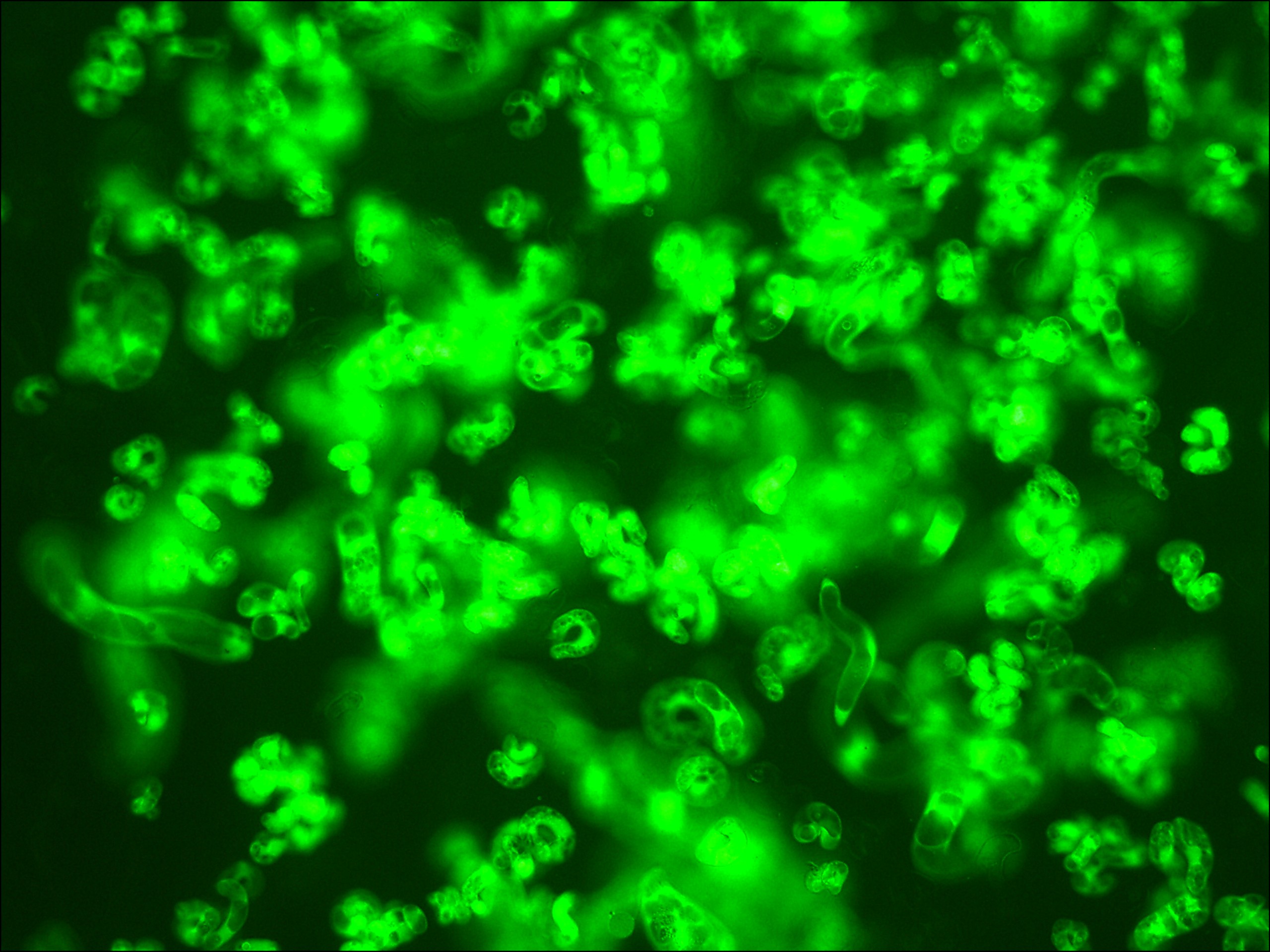

Supplement: Supplemental Information 10 [file peerj-14-21396-s010.zip › Supplementary File for Cell Vitality Fluorescence Images/0-48h Dark.jpg]

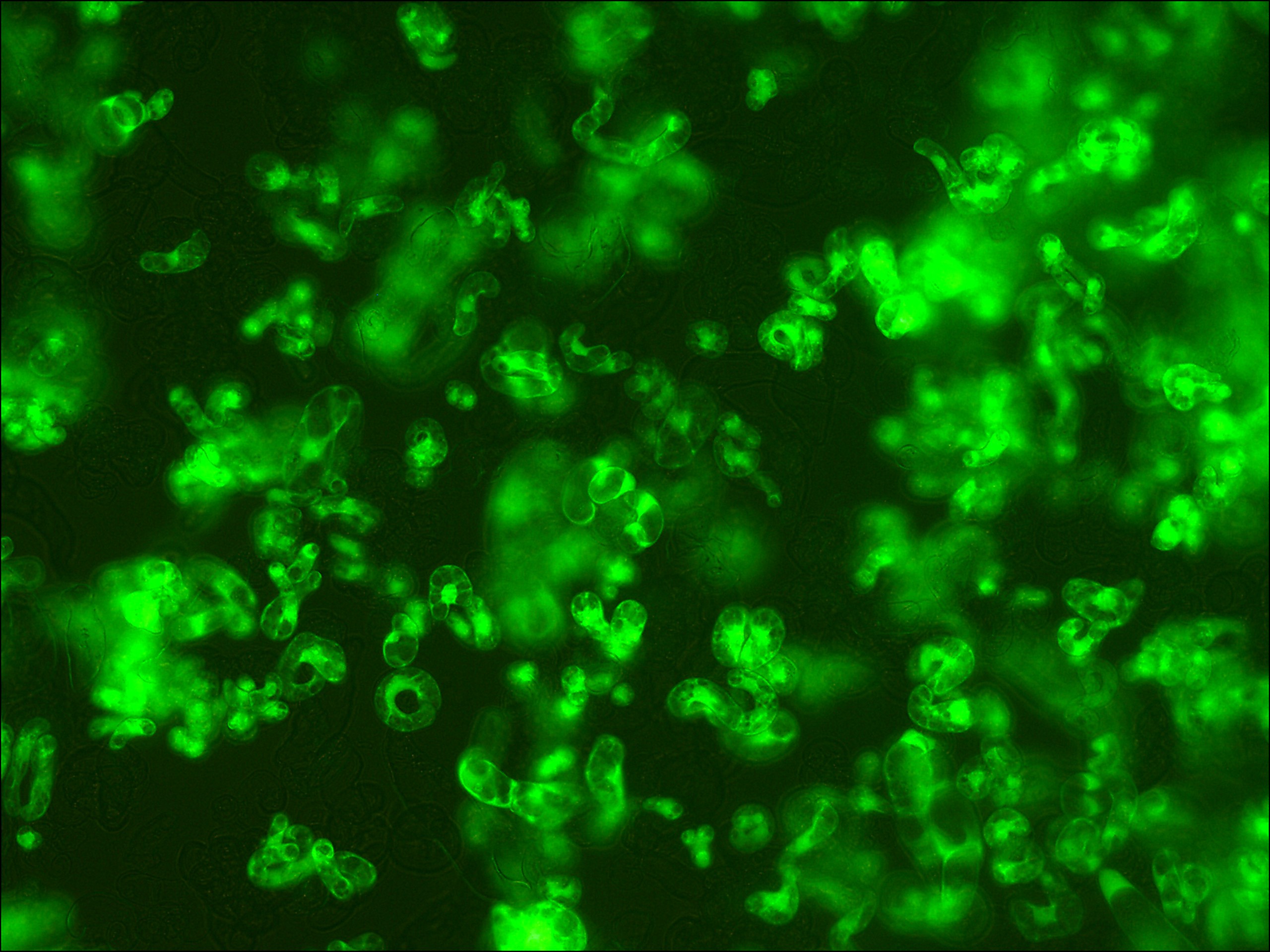

Supplement: Supplemental Information 10 [file peerj-14-21396-s010.zip › Supplementary File for Cell Vitality Fluorescence Images/0-72h Dark .jpg]

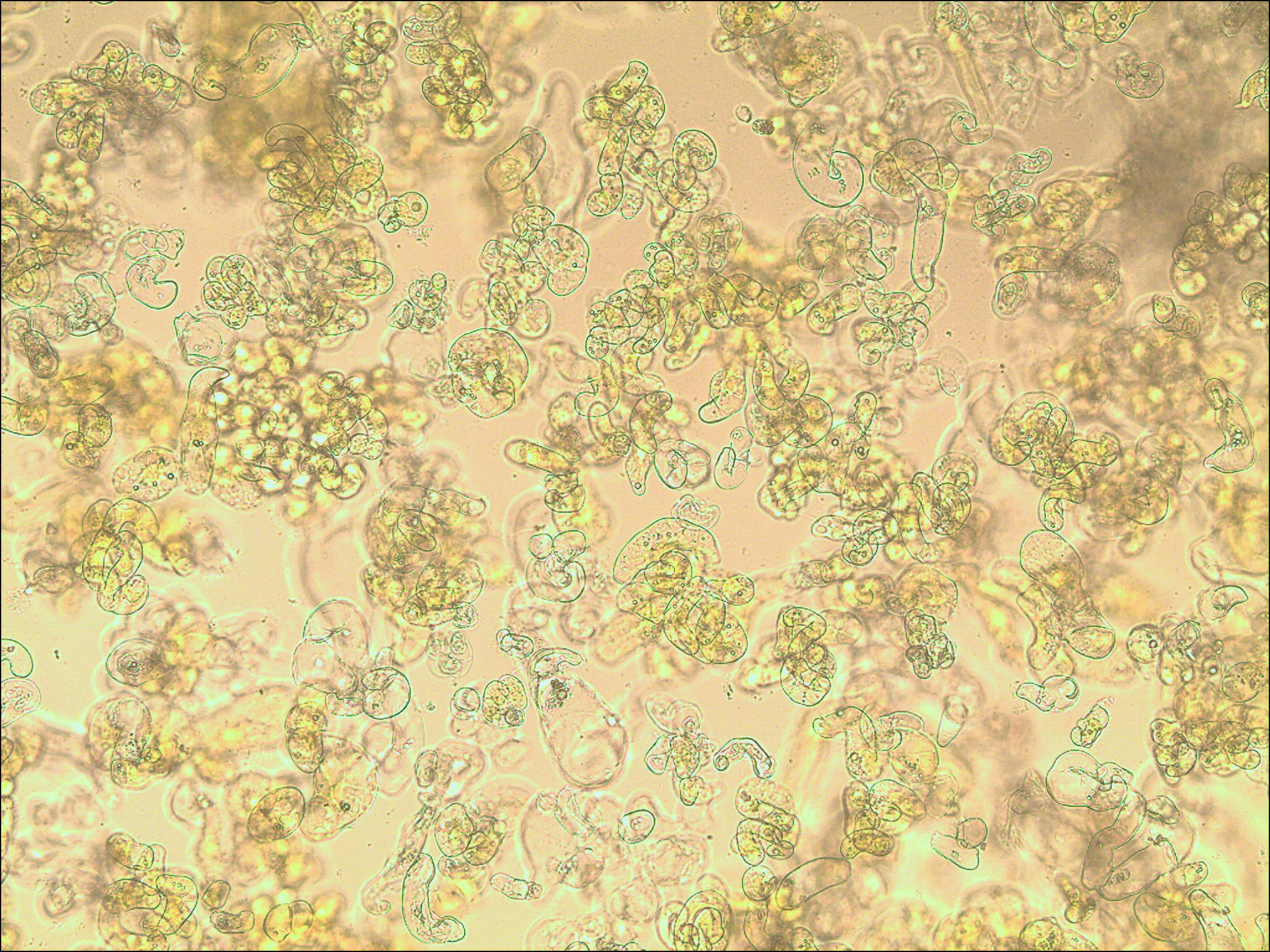

Supplement: Supplemental Information 10 [file peerj-14-21396-s010.zip › Supplementary File for Cell Vitality Fluorescence Images/0-72h Light.jpg]

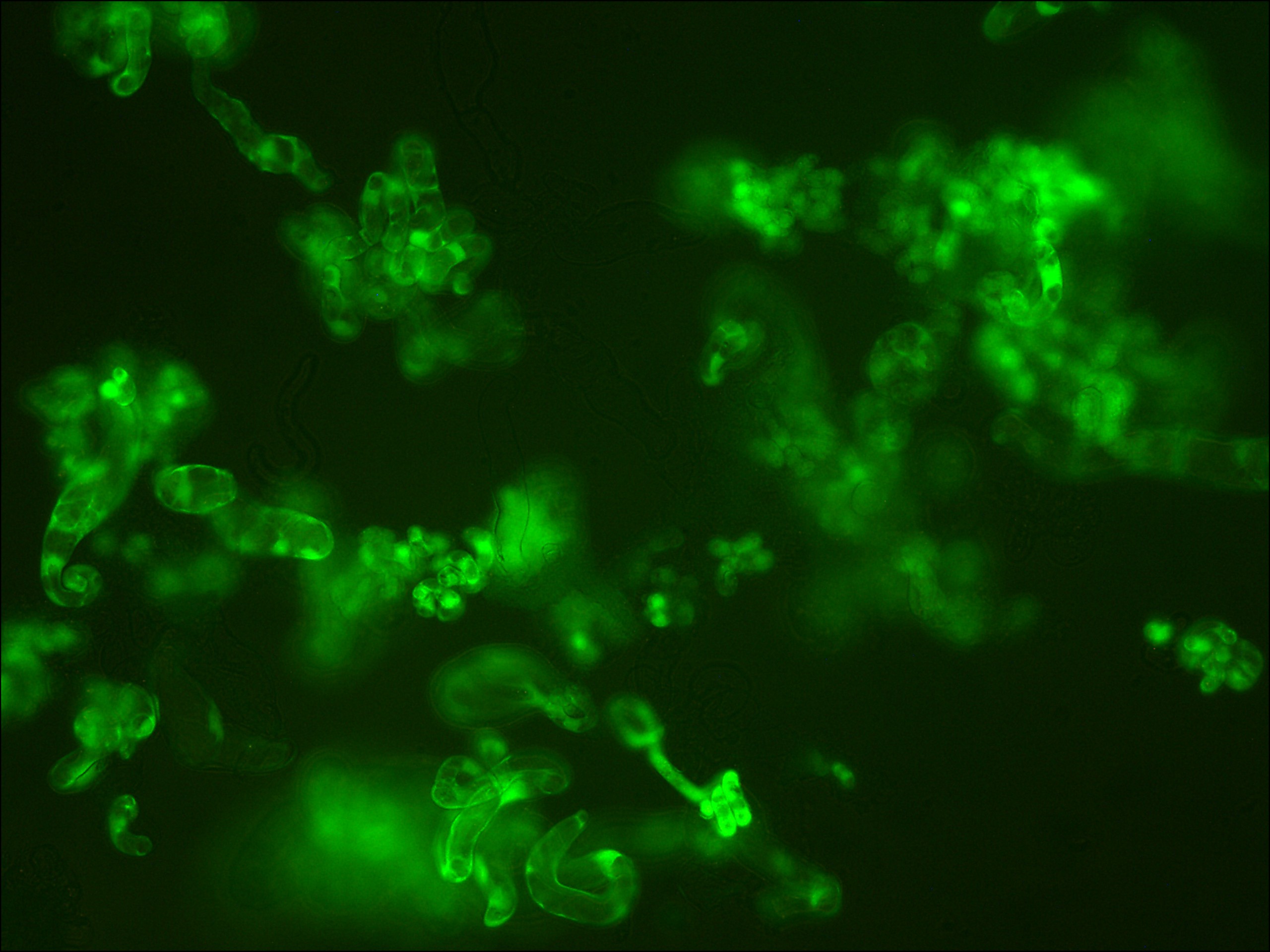

Supplement: Supplemental Information 10 [file peerj-14-21396-s010.zip › Supplementary File for Cell Vitality Fluorescence Images/10-24h Dark.jpg]

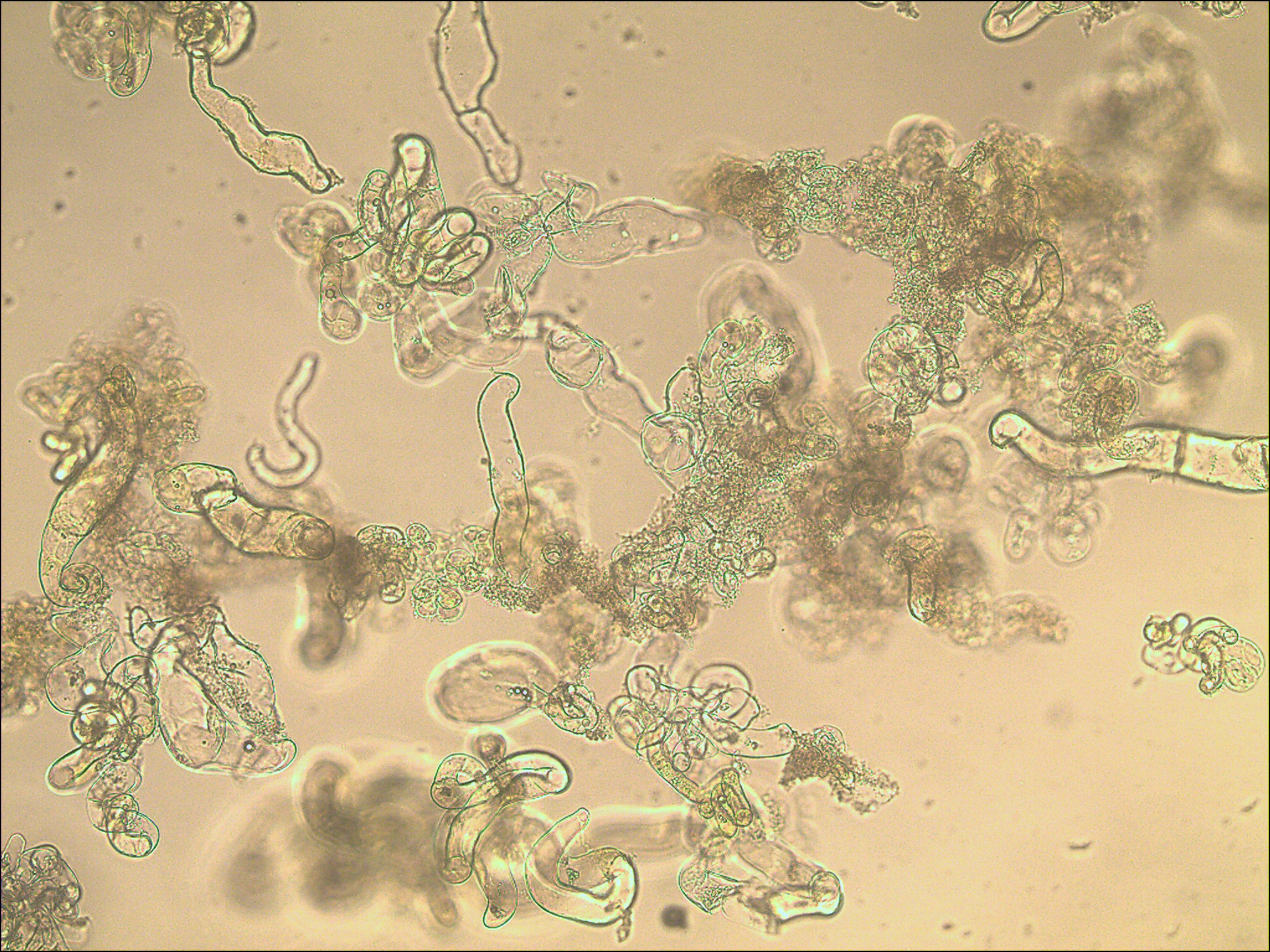

Supplement: Supplemental Information 10 [file peerj-14-21396-s010.zip › Supplementary File for Cell Vitality Fluorescence Images/10-24h Light.jpg]

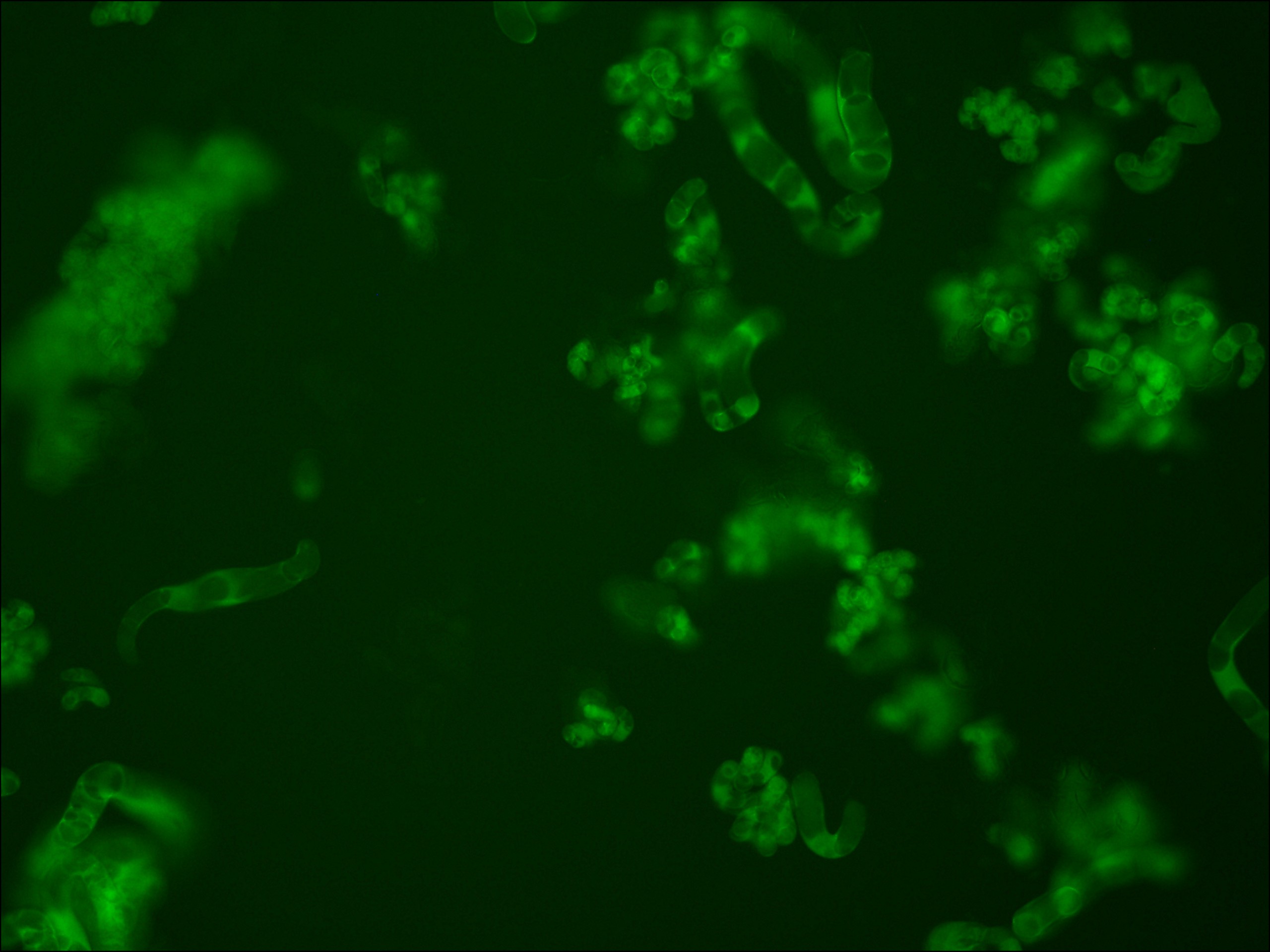

Supplement: Supplemental Information 10 [file peerj-14-21396-s010.zip › Supplementary File for Cell Vitality Fluorescence Images/10-48h Dark.jpg]

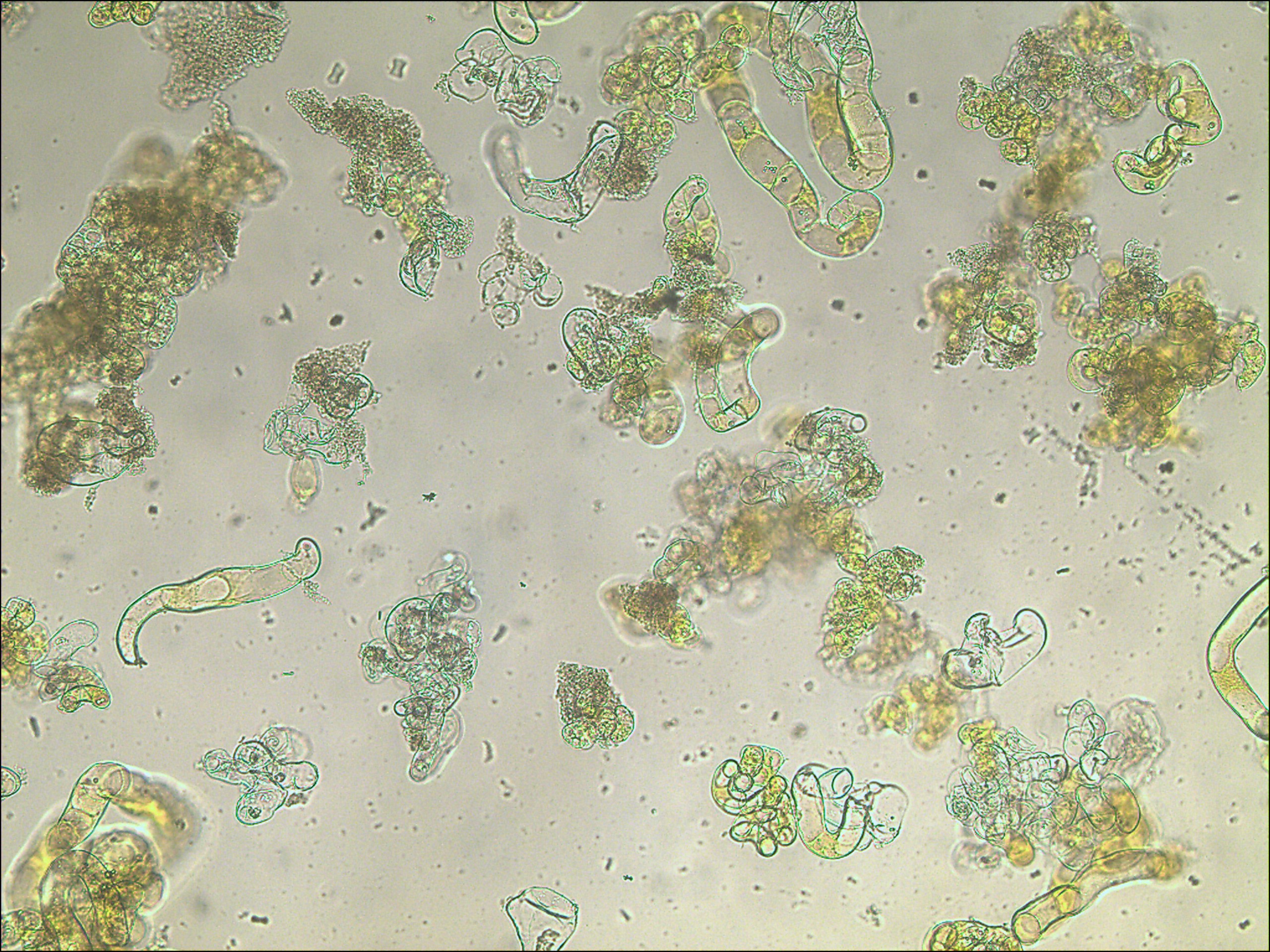

Supplement: Supplemental Information 10 [file peerj-14-21396-s010.zip › Supplementary File for Cell Vitality Fluorescence Images/10-48h Light.jpg]

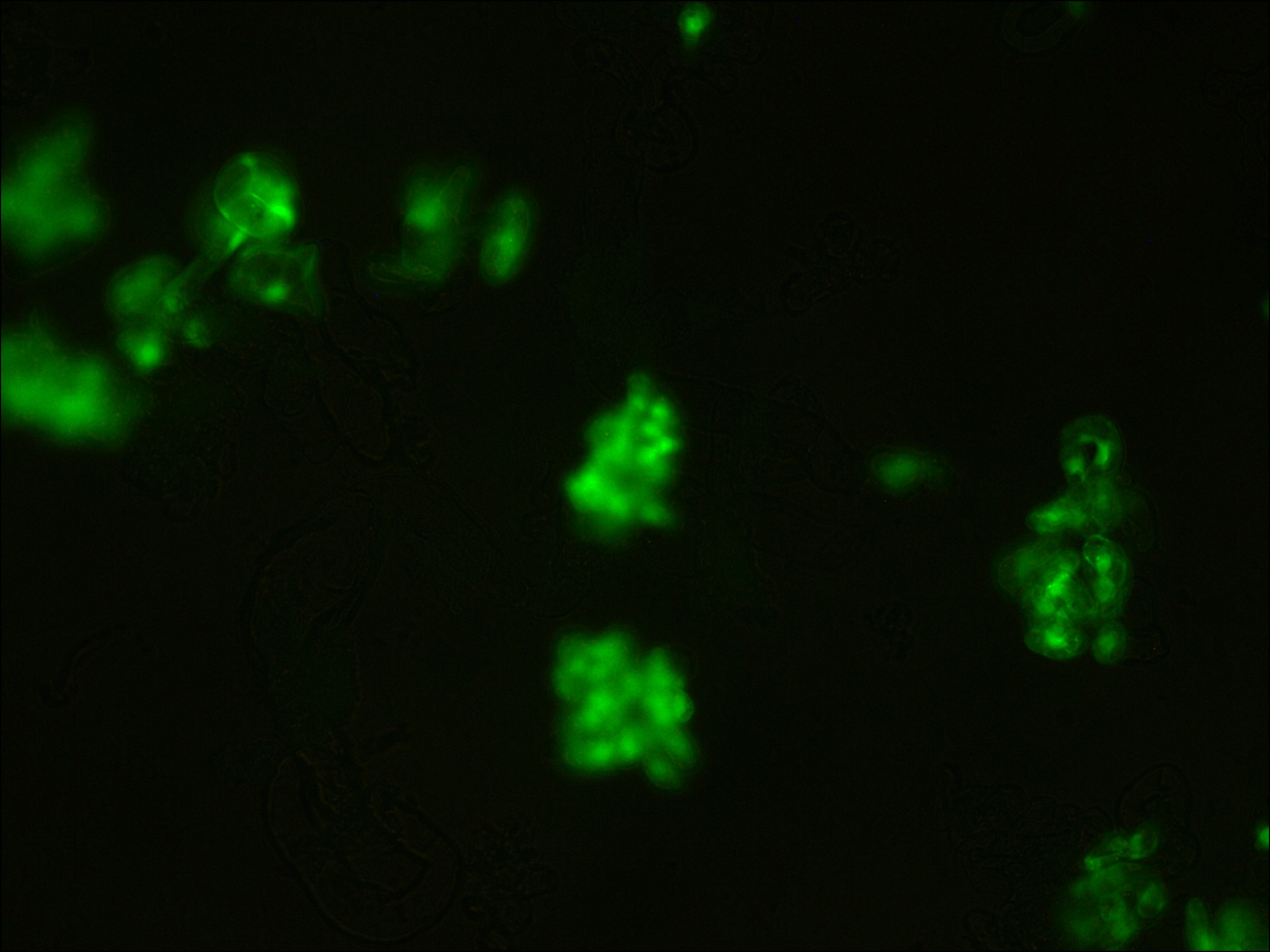

Supplement: Supplemental Information 10 [file peerj-14-21396-s010.zip › Supplementary File for Cell Vitality Fluorescence Images/10-72h Dark.jpg]

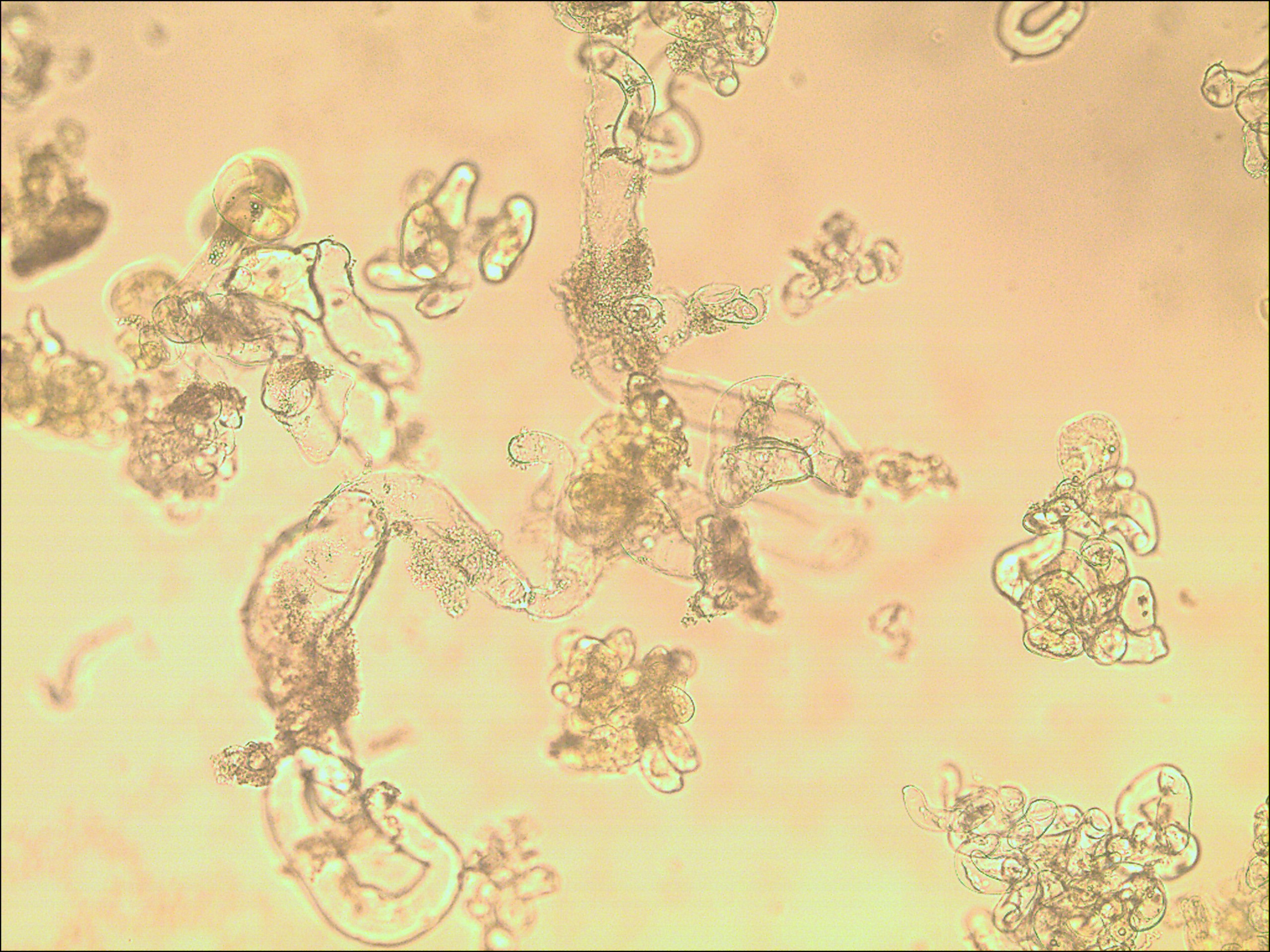

Supplement: Supplemental Information 10 [file peerj-14-21396-s010.zip › Supplementary File for Cell Vitality Fluorescence Images/10-72h Light.jpg]

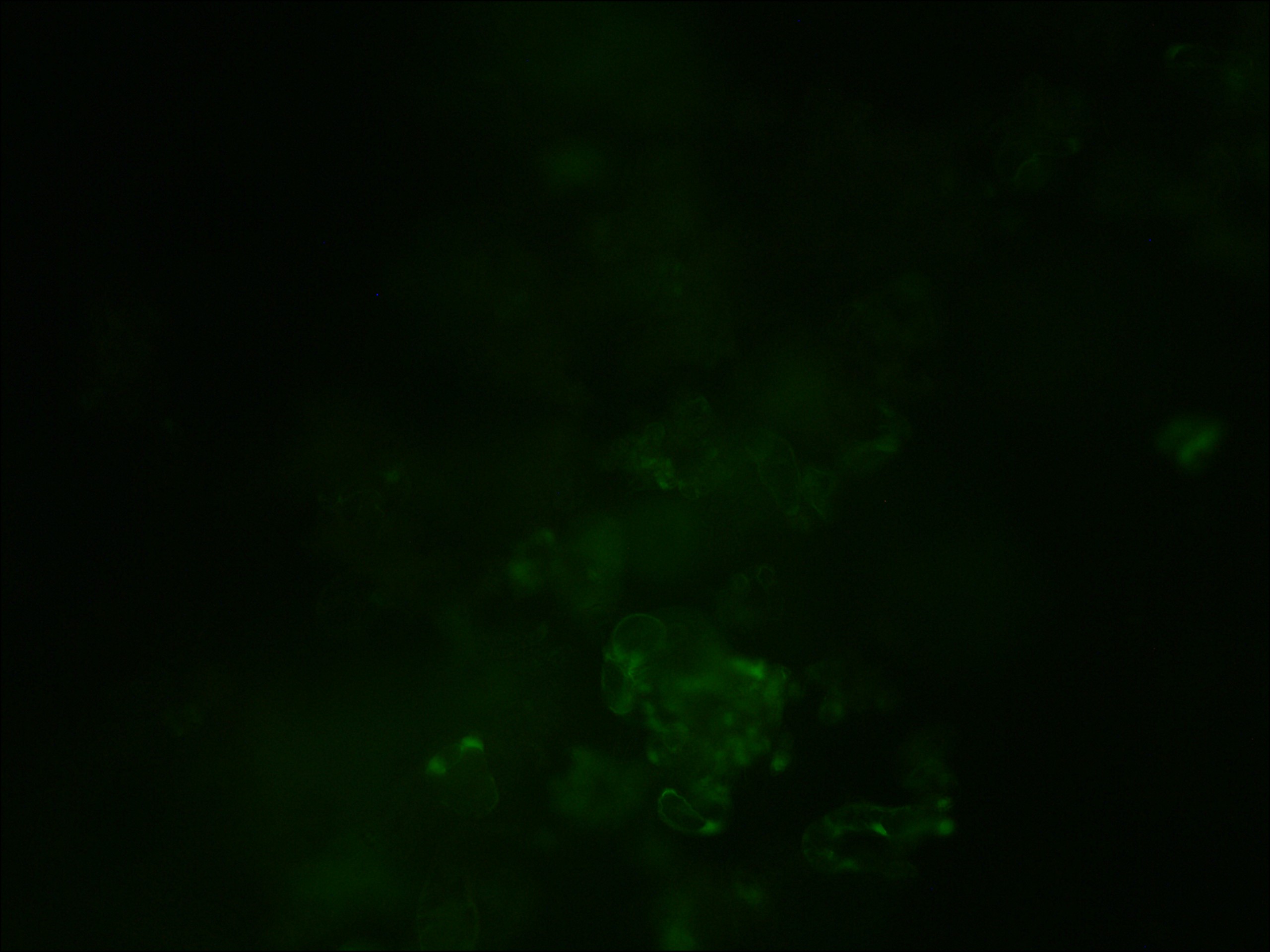

Supplement: Supplemental Information 10 [file peerj-14-21396-s010.zip › Supplementary File for Cell Vitality Fluorescence Images/20-24h Dark.jpg]

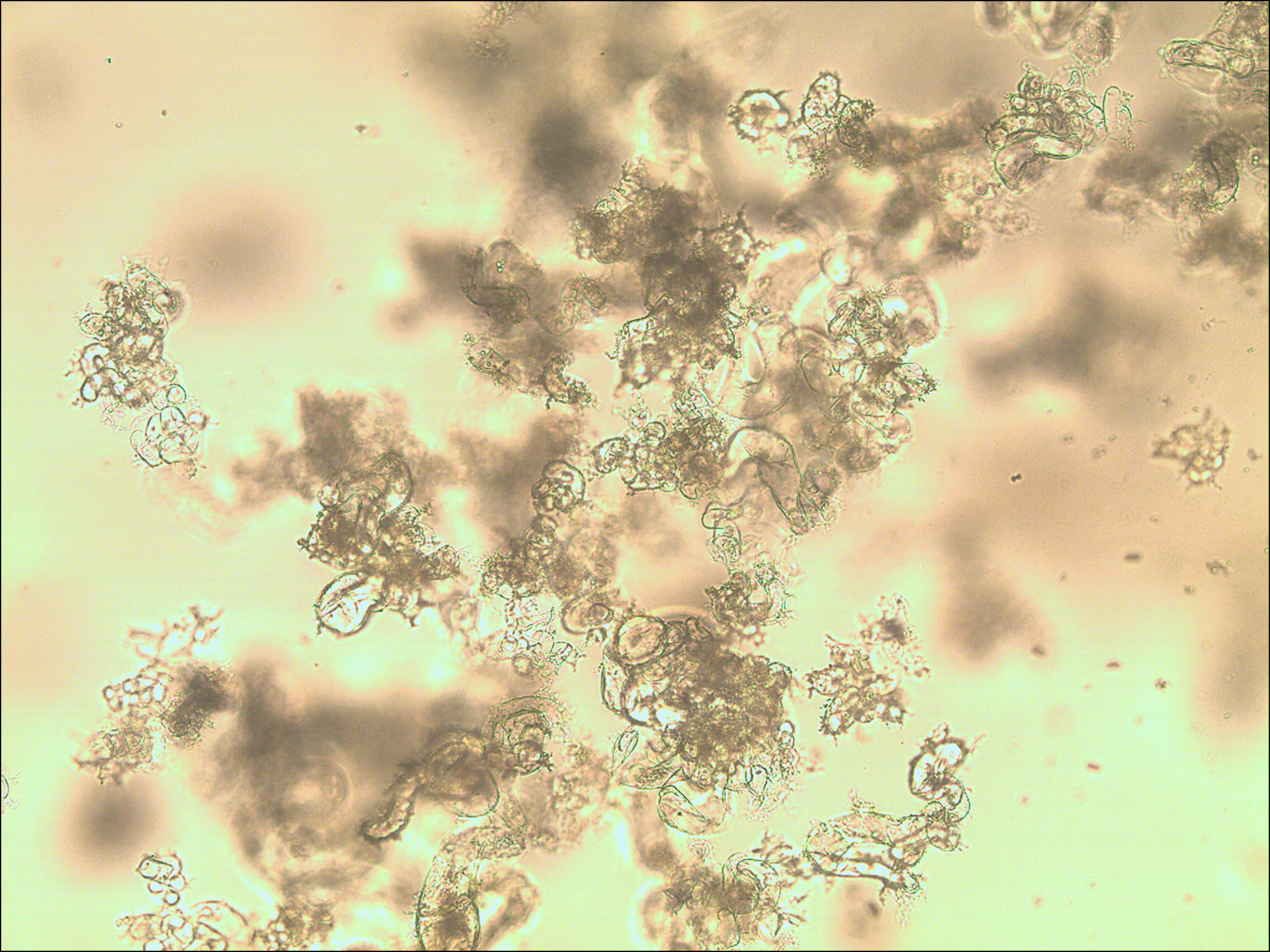

Supplement: Supplemental Information 10 [file peerj-14-21396-s010.zip › Supplementary File for Cell Vitality Fluorescence Images/20-24h Light.jpg]

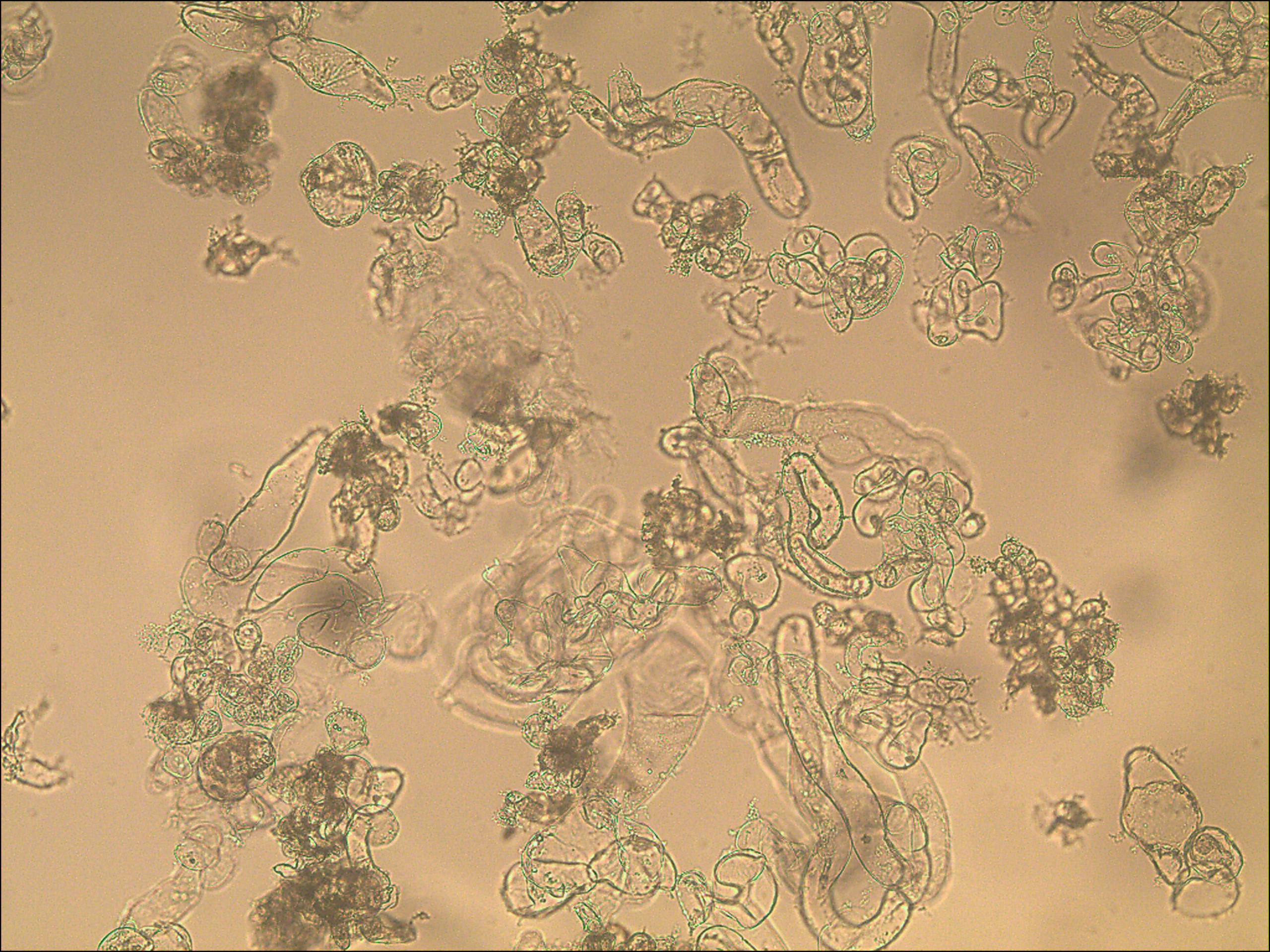

Supplement: Supplemental Information 10 [file peerj-14-21396-s010.zip › Supplementary File for Cell Vitality Fluorescence Images/20-48h Light.jpg]

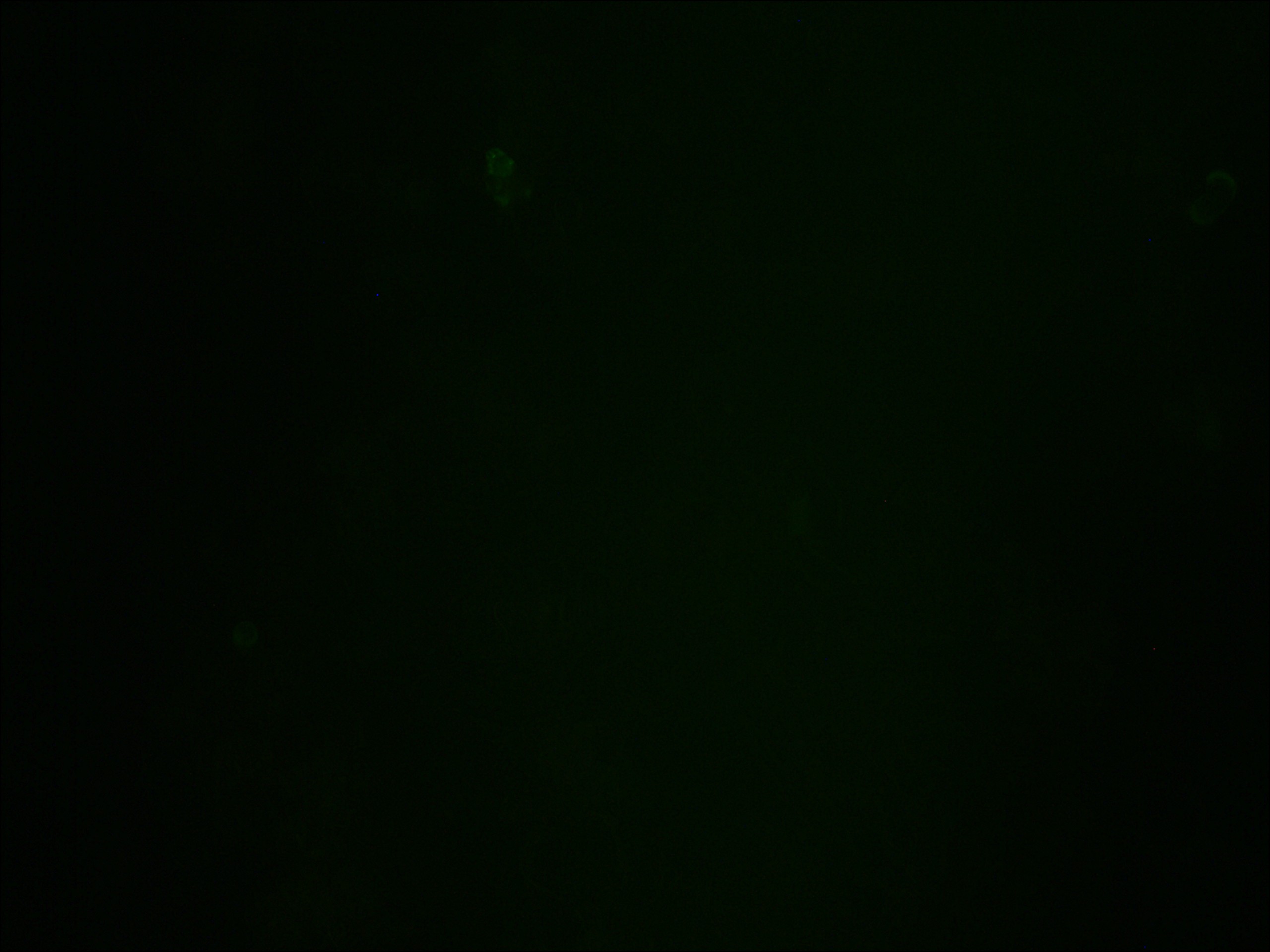

Supplement: Supplemental Information 10 [file peerj-14-21396-s010.zip › Supplementary File for Cell Vitality Fluorescence Images/20-48h Dark.jpg]

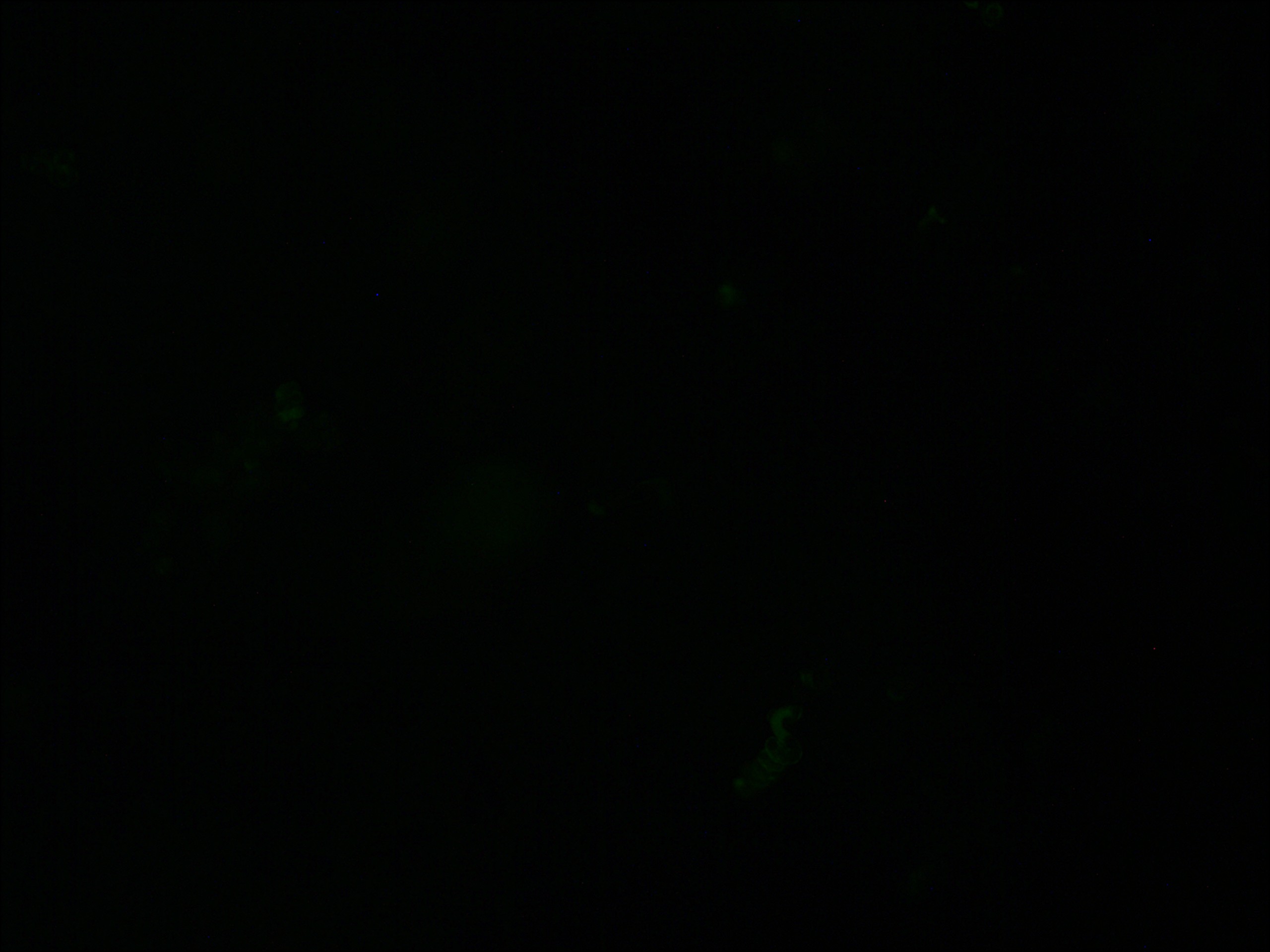

Supplement: Supplemental Information 10 [file peerj-14-21396-s010.zip › Supplementary File for Cell Vitality Fluorescence Images/20-72h Dark.jpg]

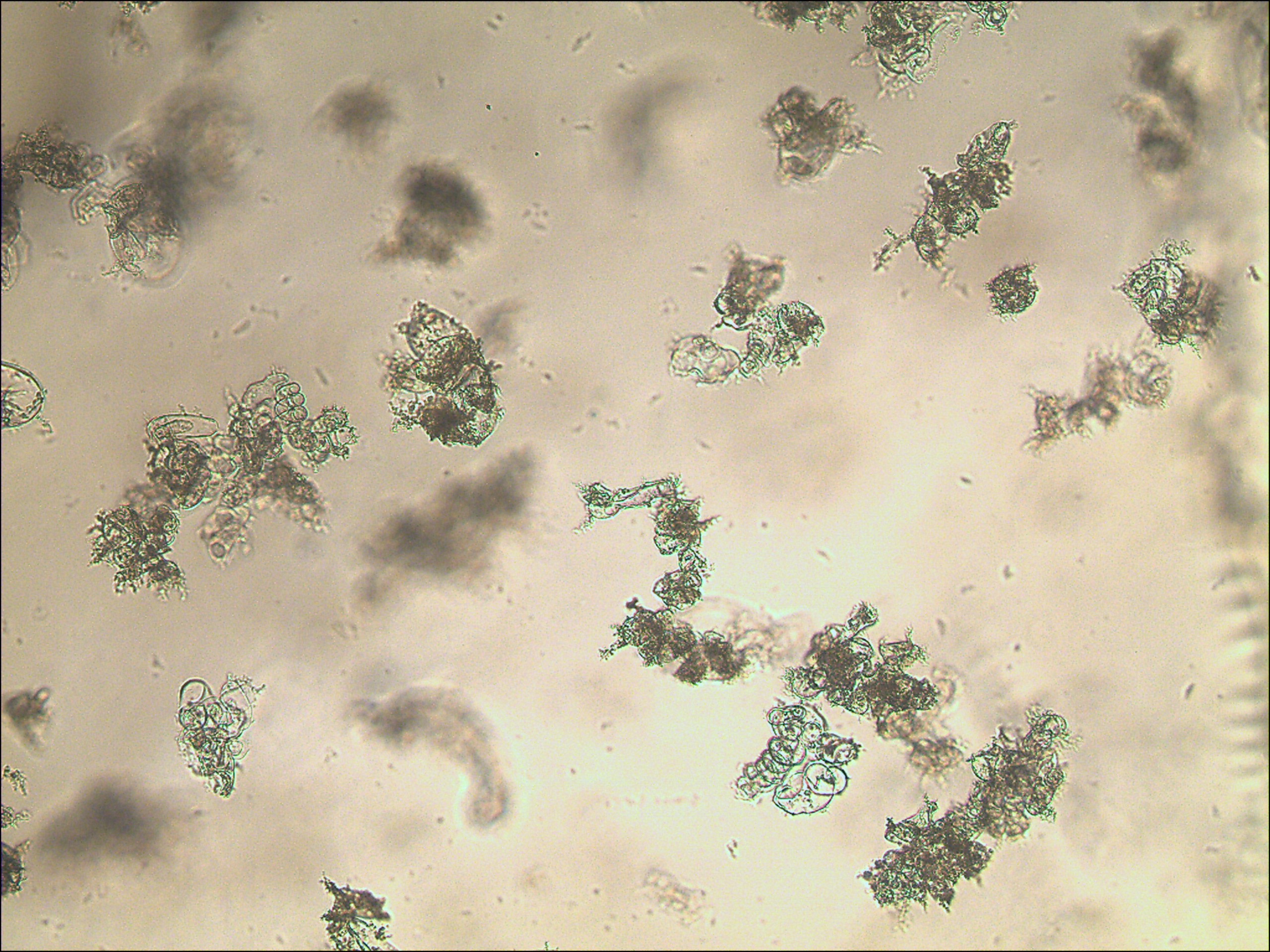

Supplement: Supplemental Information 10 [file peerj-14-21396-s010.zip › Supplementary File for Cell Vitality Fluorescence Images/20-72h Light.jpg]

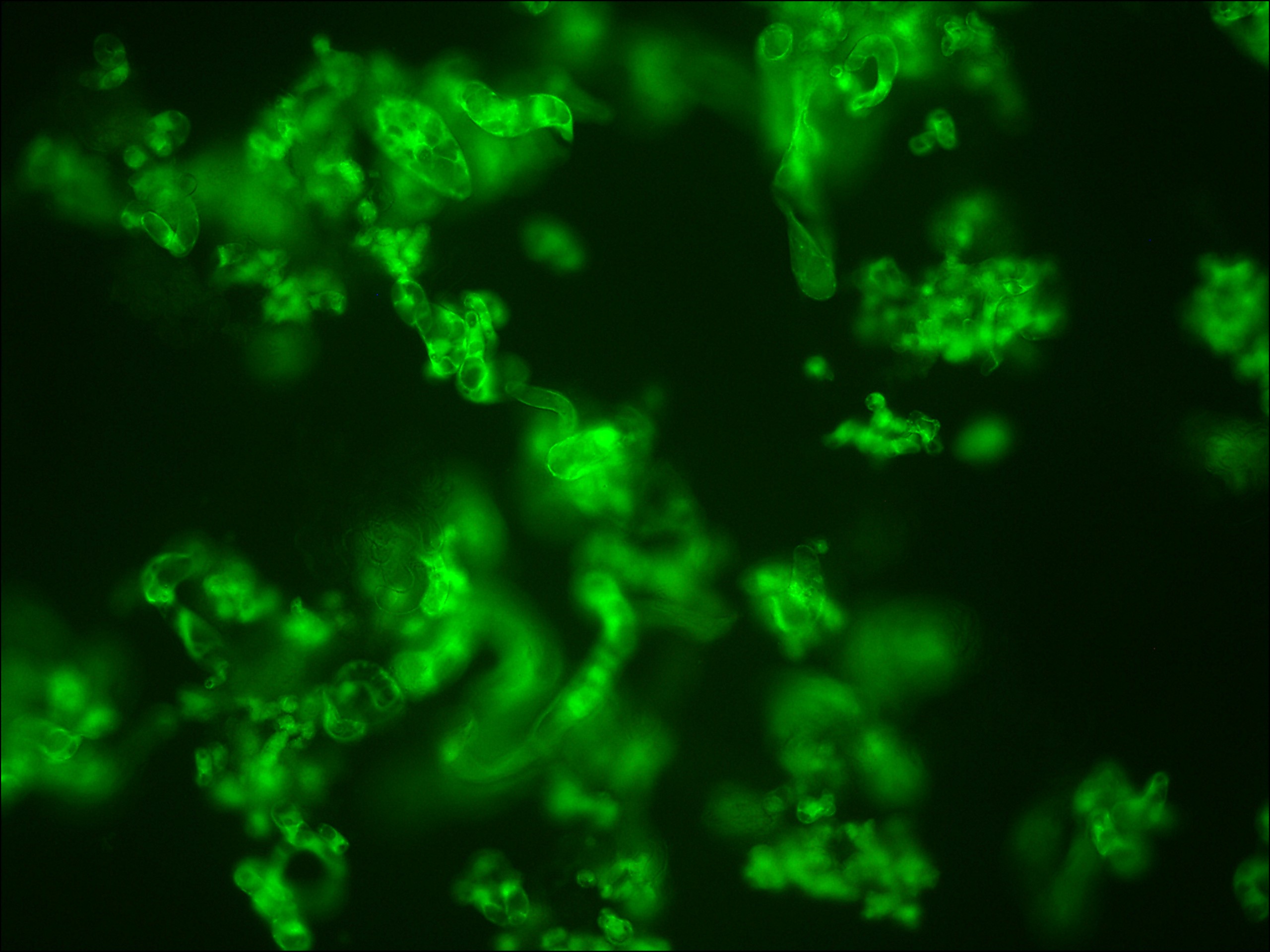

Supplement: Supplemental Information 10 [file peerj-14-21396-s010.zip › Supplementary File for Cell Vitality Fluorescence Images/5-24h Dark.jpg]

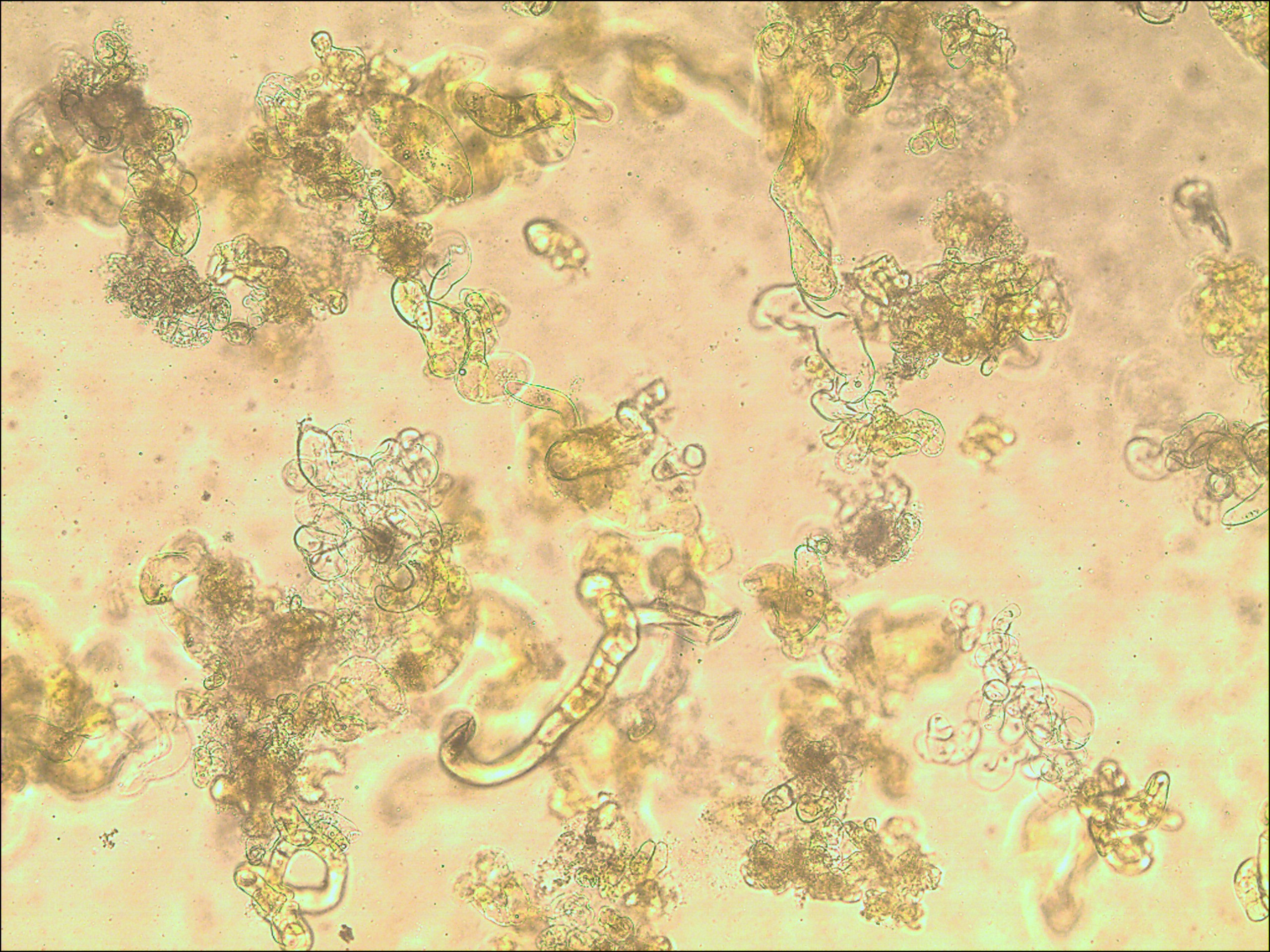

Supplement: Supplemental Information 10 [file peerj-14-21396-s010.zip › Supplementary File for Cell Vitality Fluorescence Images/5-24h Light.jpg]

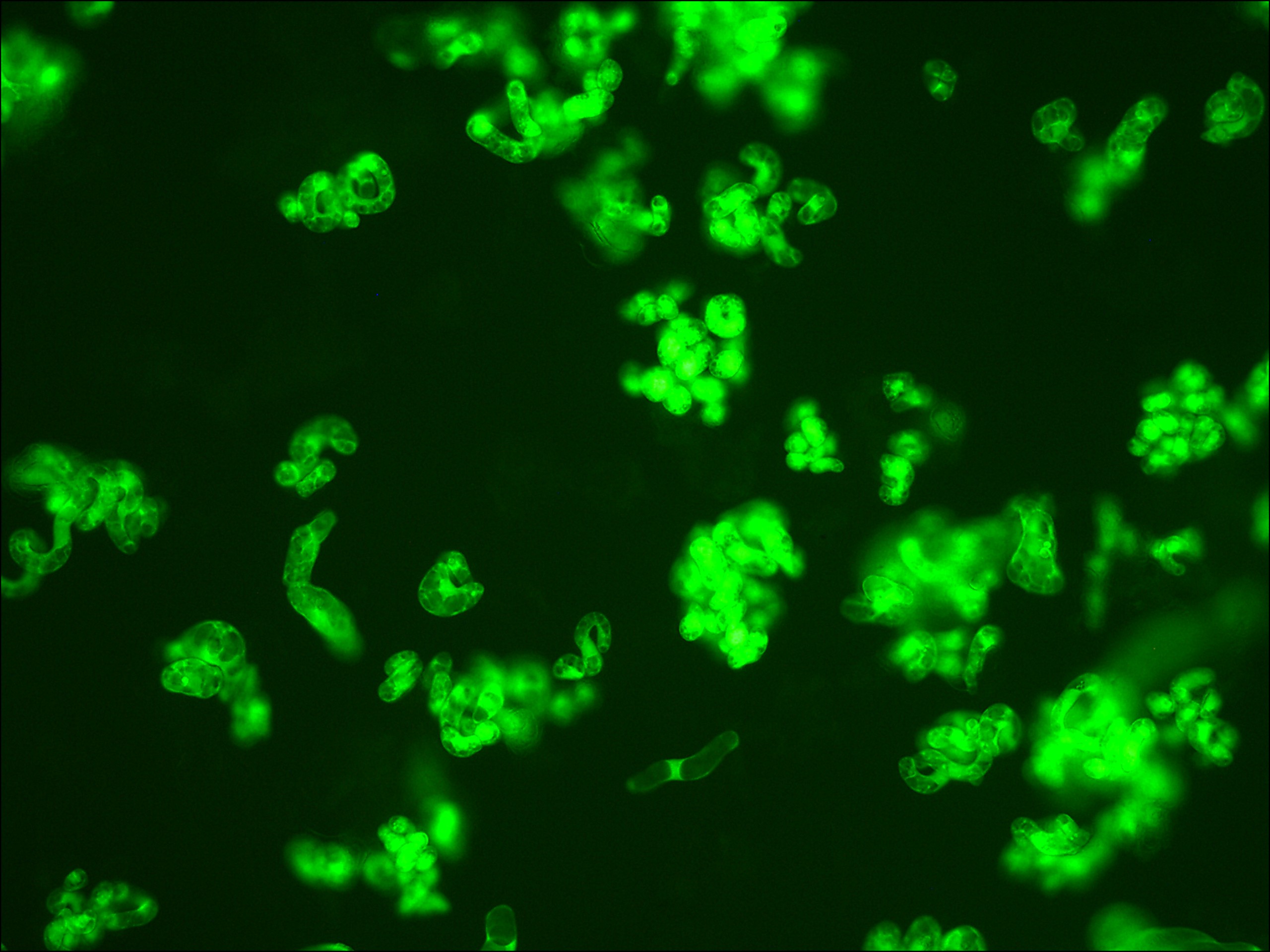

Supplement: Supplemental Information 10 [file peerj-14-21396-s010.zip › Supplementary File for Cell Vitality Fluorescence Images/5-48h Dark.jpg]

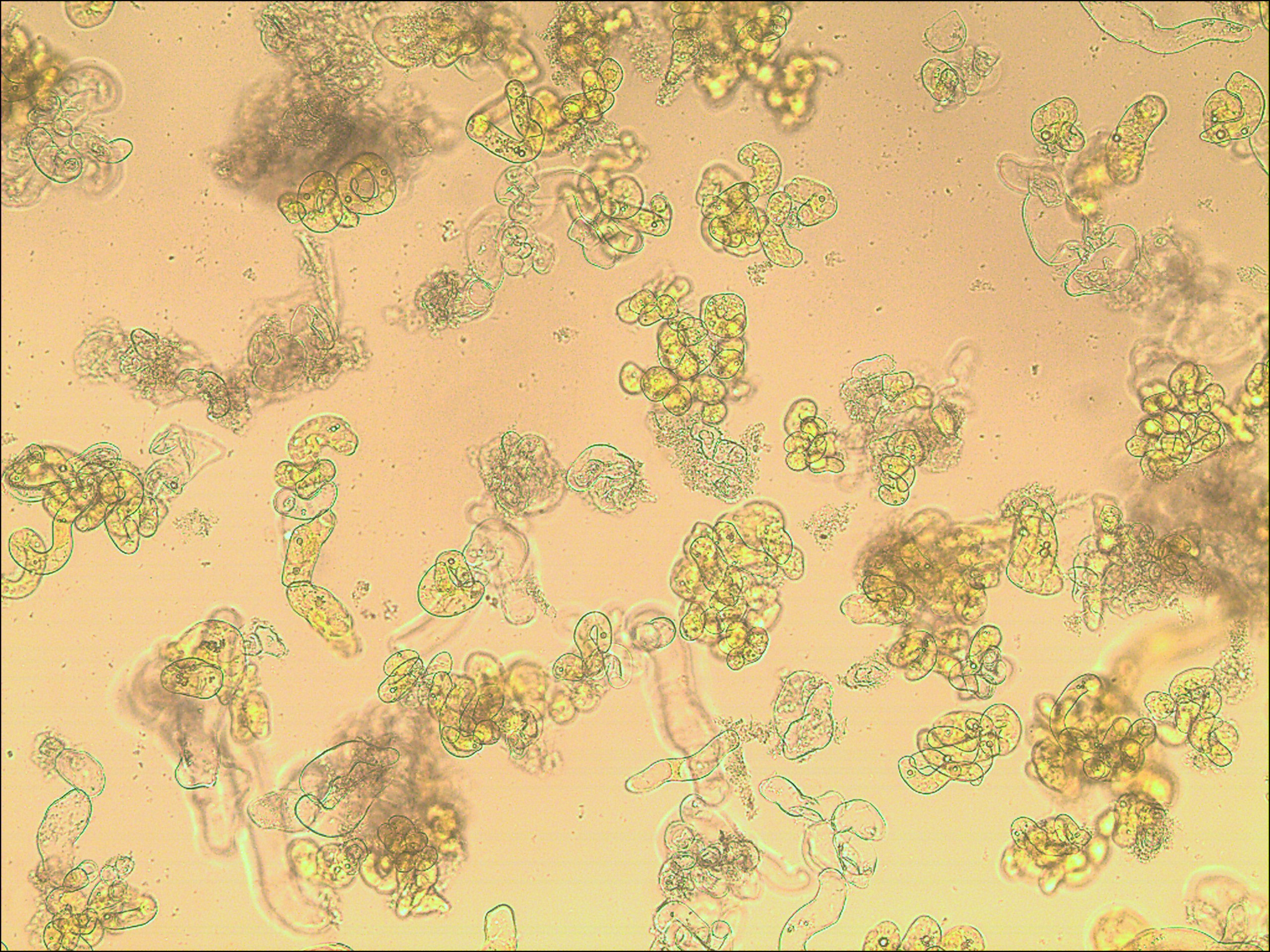

Supplement: Supplemental Information 10 [file peerj-14-21396-s010.zip › Supplementary File for Cell Vitality Fluorescence Images/5-48h Light.jpg]

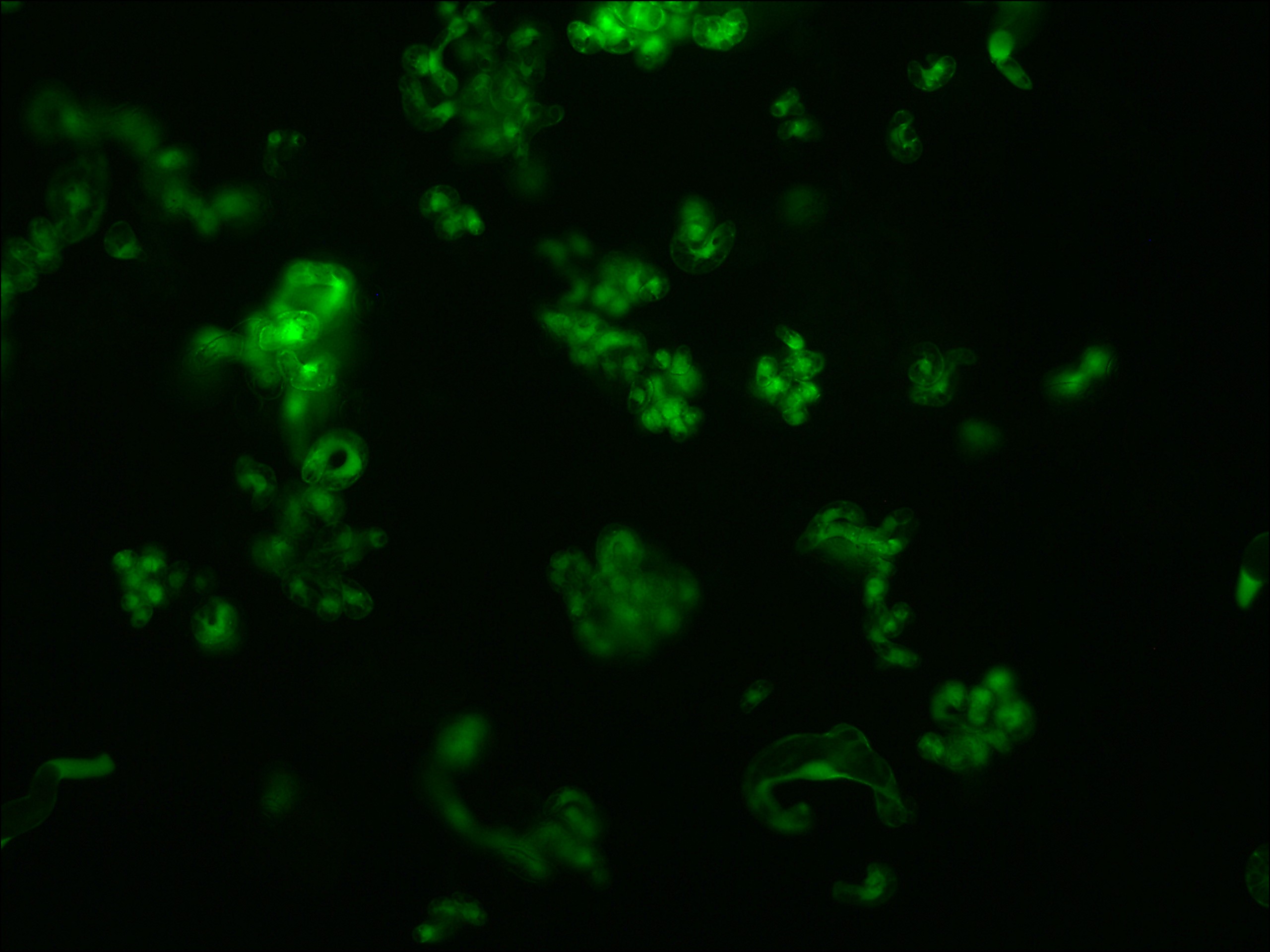

Supplement: Supplemental Information 10 [file peerj-14-21396-s010.zip › Supplementary File for Cell Vitality Fluorescence Images/5-72h Dark.jpg]

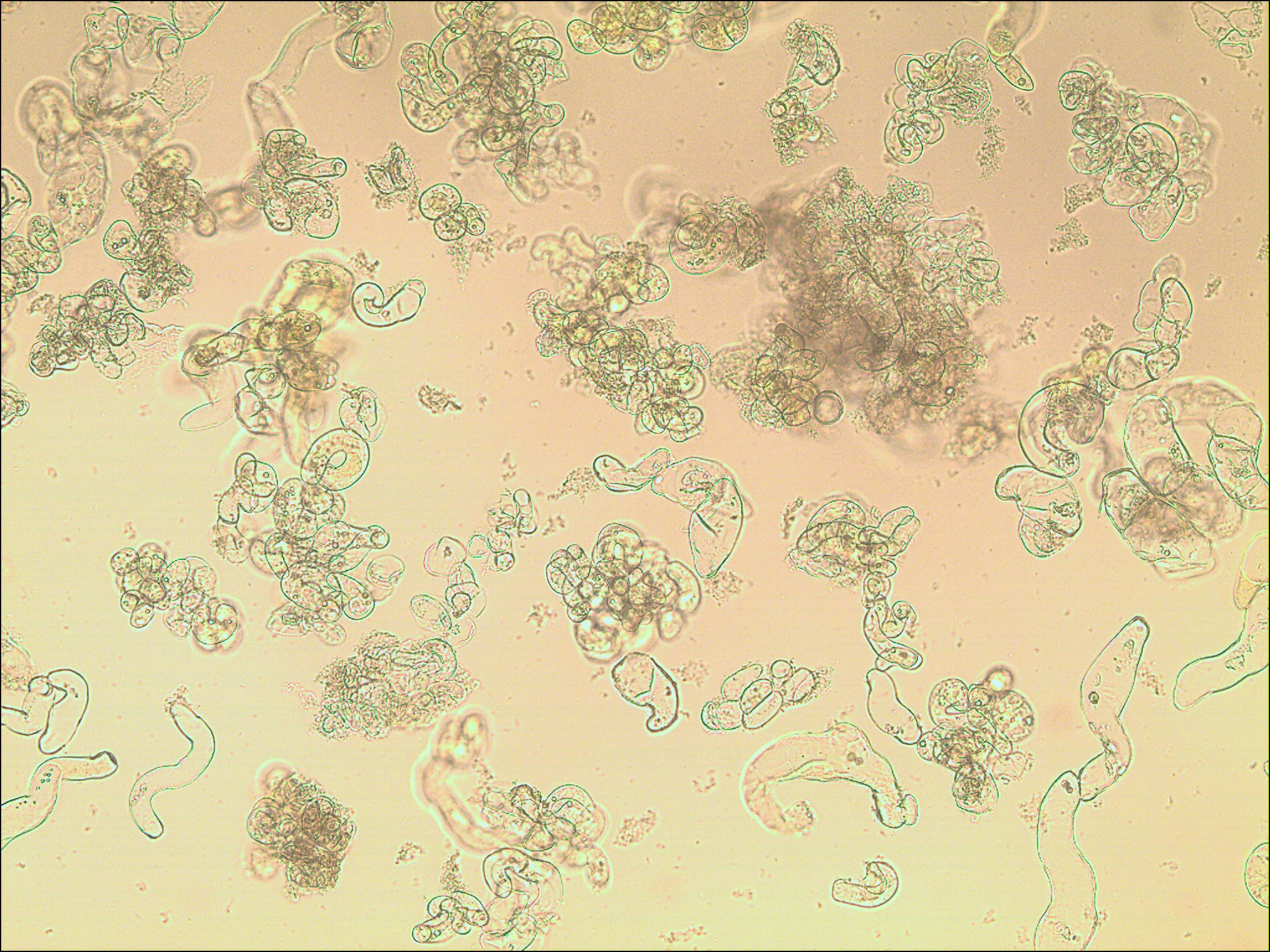

Supplement: Supplemental Information 10 [file peerj-14-21396-s010.zip › Supplementary File for Cell Vitality Fluorescence Images/5-72h Light.jpg]

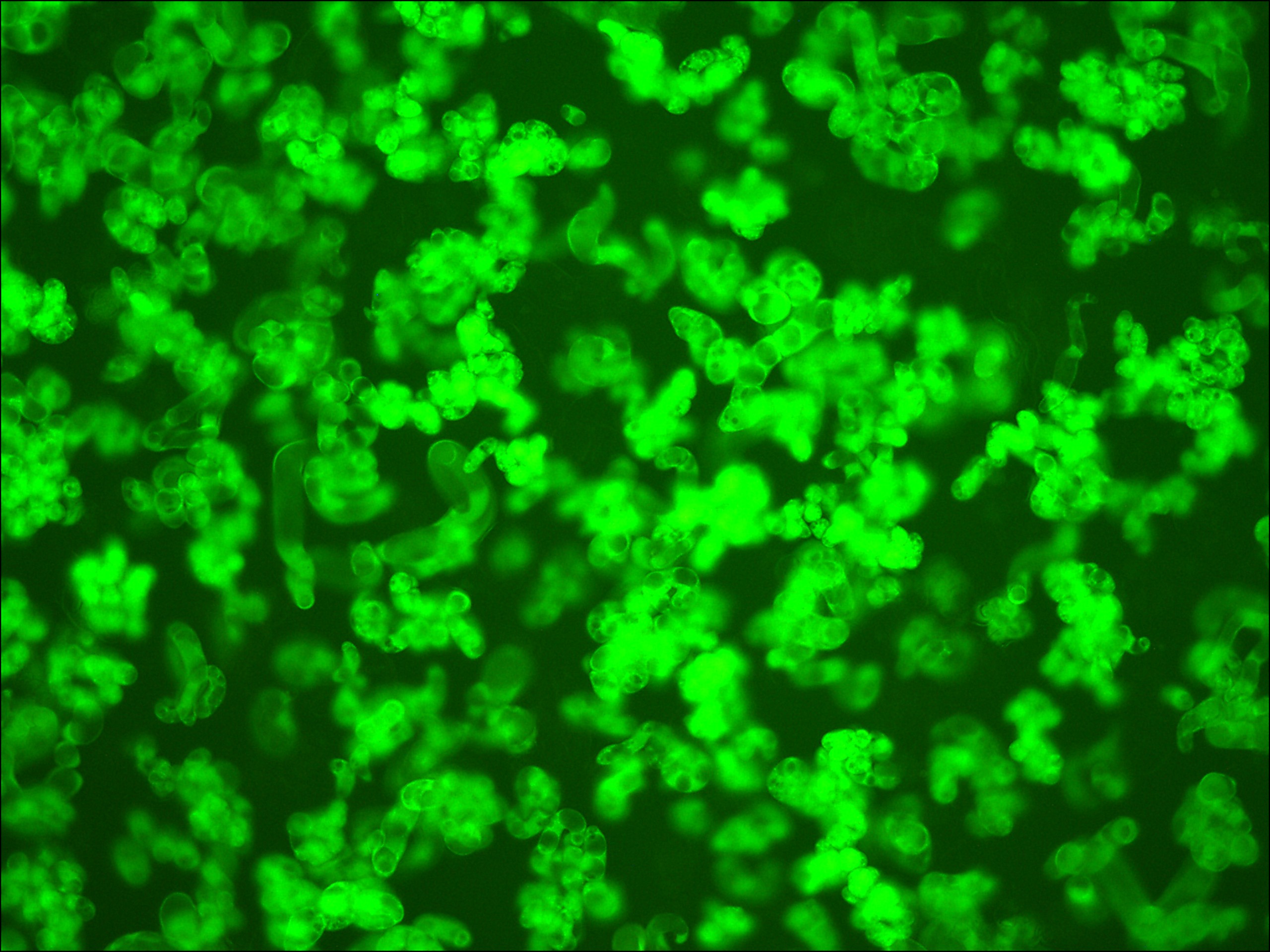

Supplement: Supplemental Information 10 [file peerj-14-21396-s010.zip › Supplementary File for Cell Vitality Fluorescence Images/Before the experiment Dark.jpg]

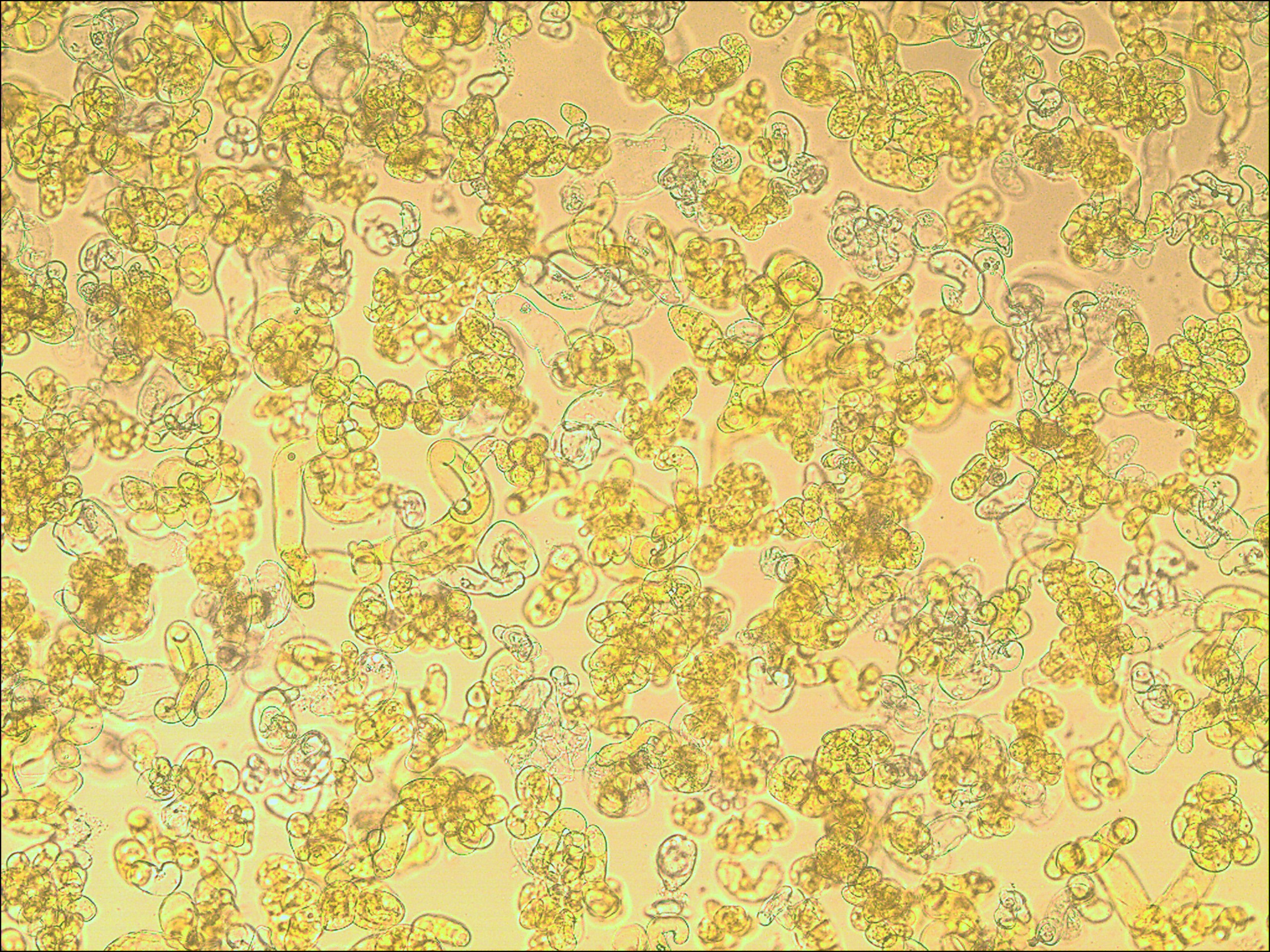

Supplement: Supplemental Information 10 [file peerj-14-21396-s010.zip › Supplementary File for Cell Vitality Fluorescence Images/Before the experiment Light.jpg]

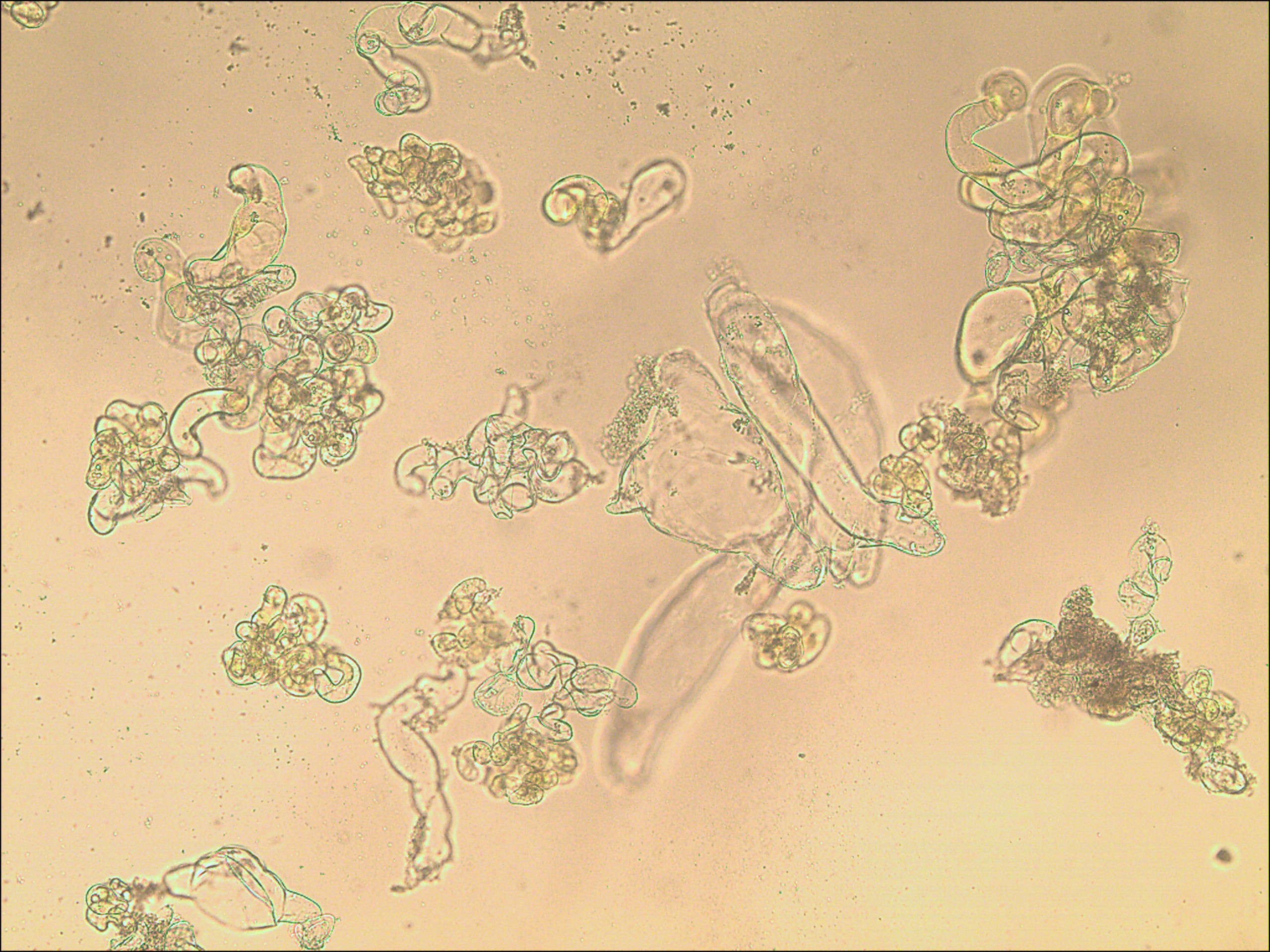

Supplement: Supplemental Information 11 [file peerj-14-21396-s011.zip › Supplementary File for Cell Morphology Images/10-10×.jpg]

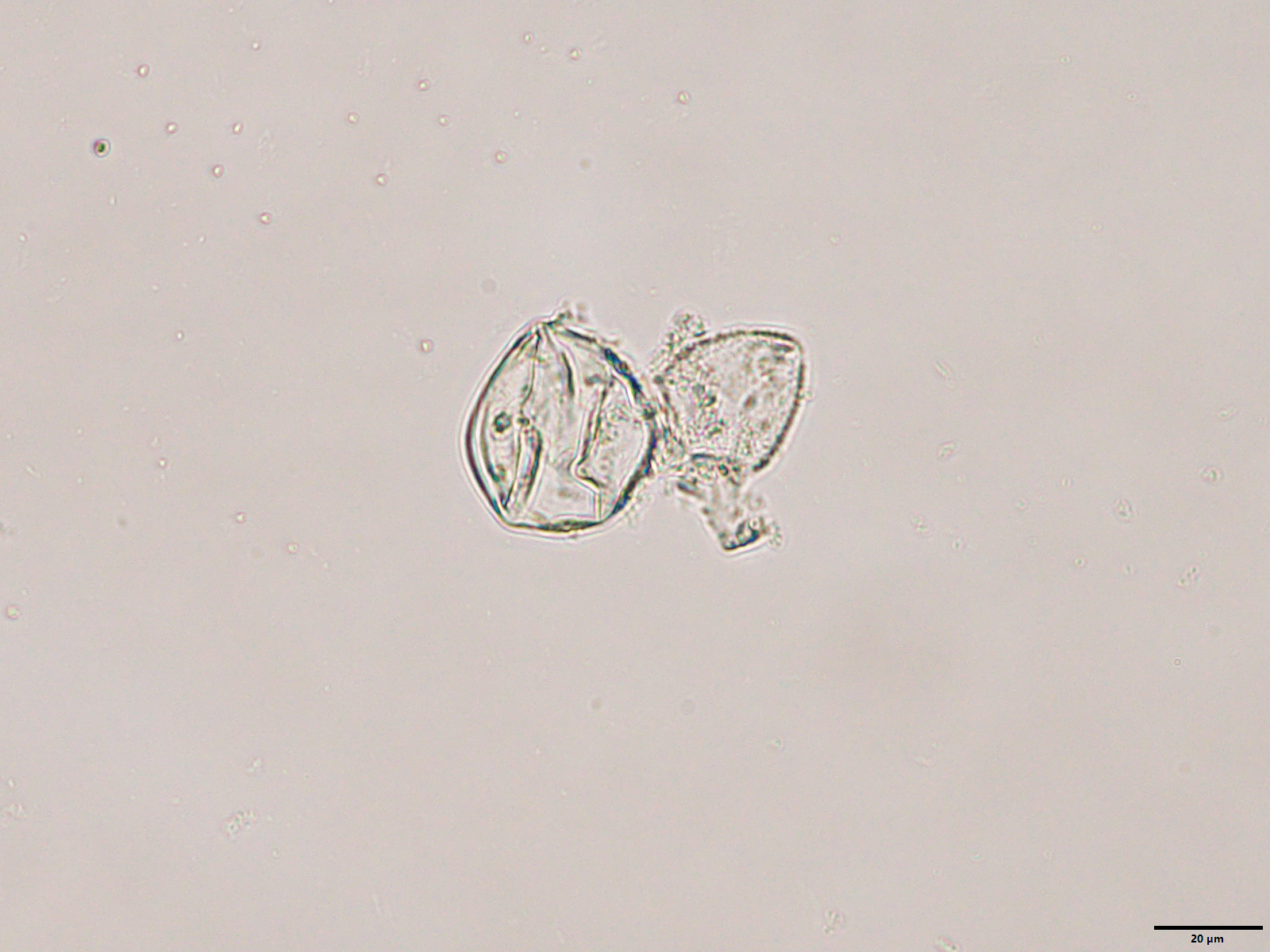

Supplement: Supplemental Information 11 [file peerj-14-21396-s011.zip › Supplementary File for Cell Morphology Images/10-40×.tif]

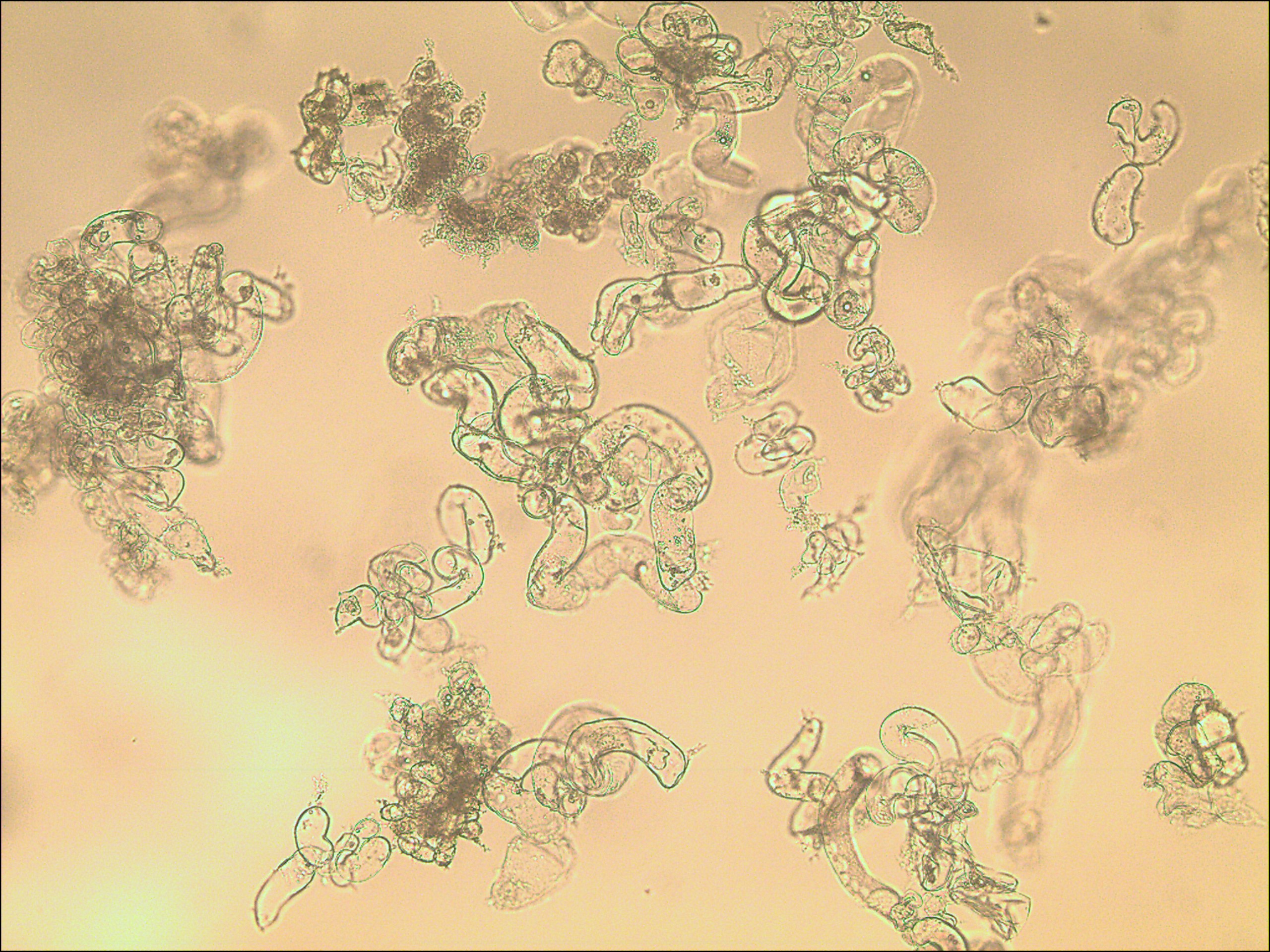

Supplement: Supplemental Information 11 [file peerj-14-21396-s011.zip › Supplementary File for Cell Morphology Images/20-10×.jpg]

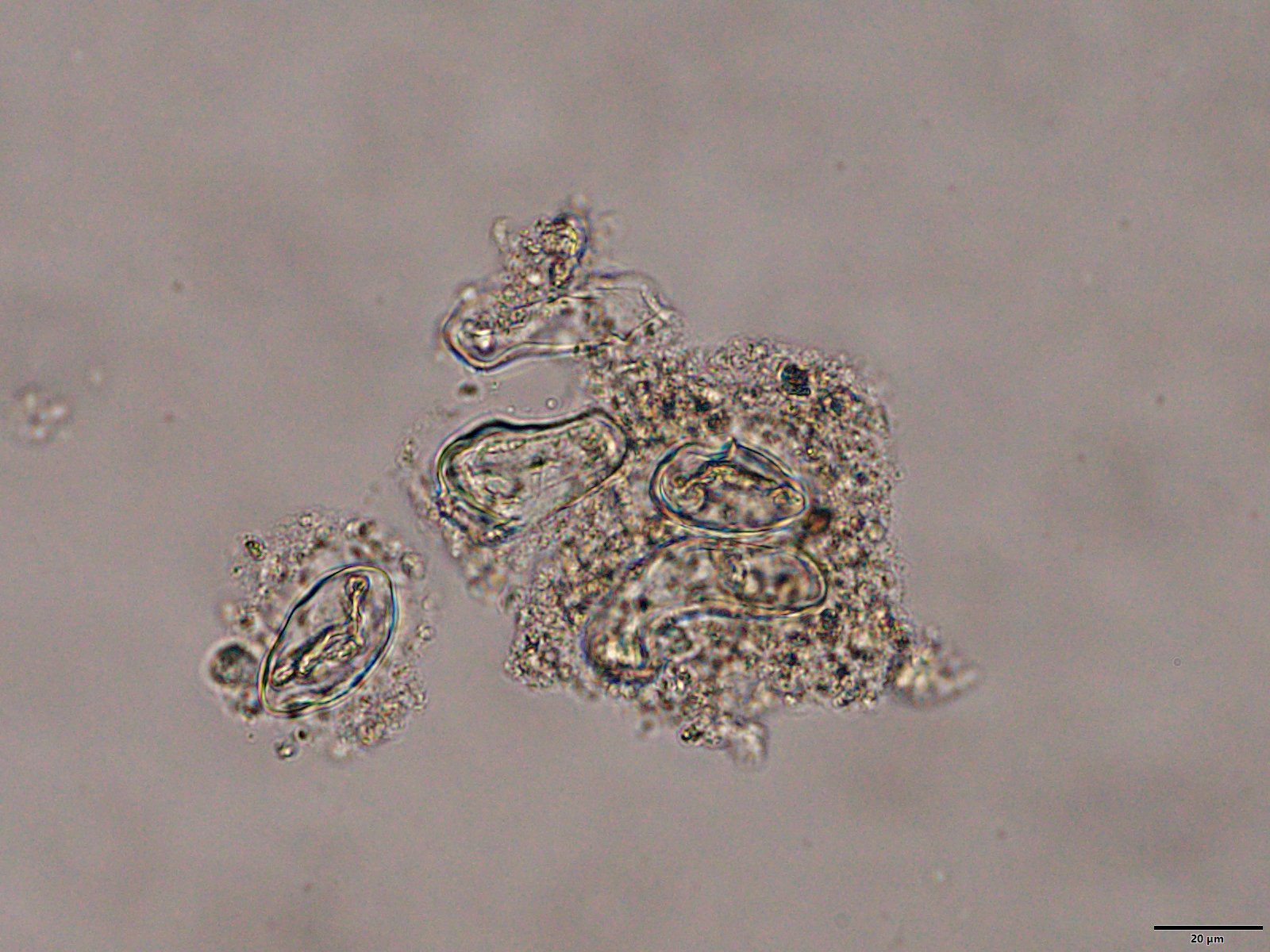

Supplement: Supplemental Information 11 [file peerj-14-21396-s011.zip › Supplementary File for Cell Morphology Images/20-40×.tif]

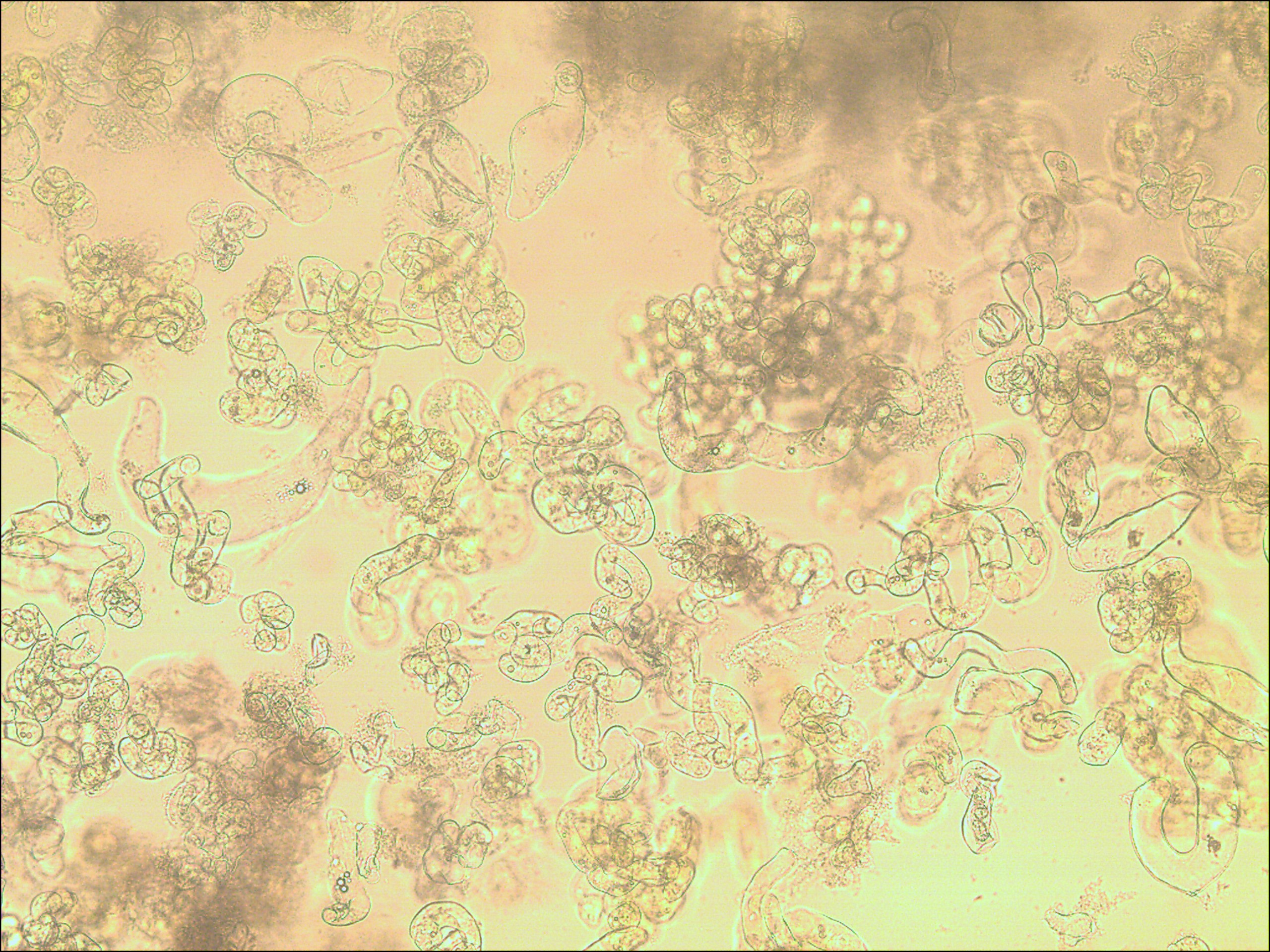

Supplement: Supplemental Information 11 [file peerj-14-21396-s011.zip › Supplementary File for Cell Morphology Images/5-10×.jpg]

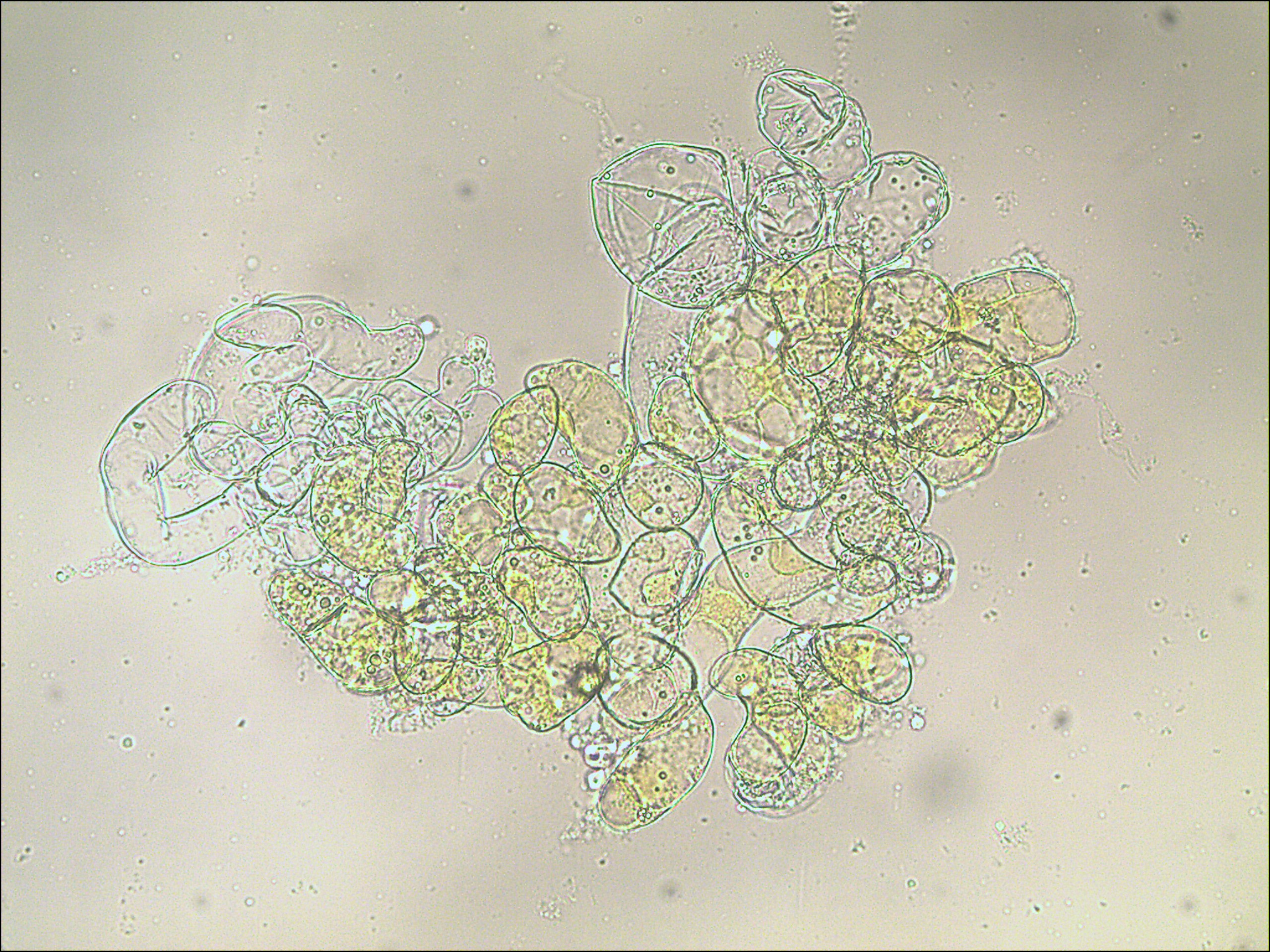

Supplement: Supplemental Information 11 [file peerj-14-21396-s011.zip › Supplementary File for Cell Morphology Images/5-40×.jpg]

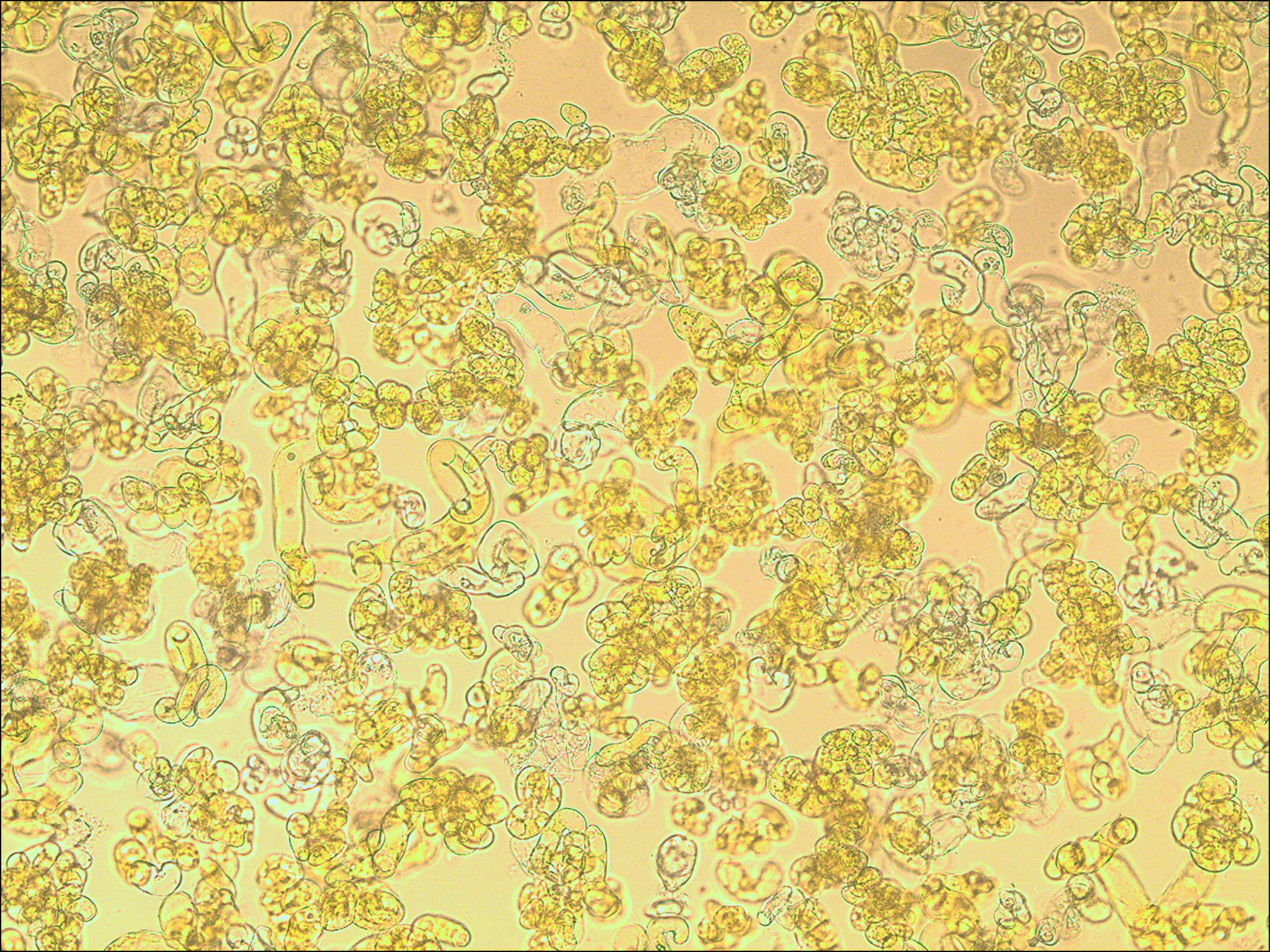

Supplement: Supplemental Information 11 [file peerj-14-21396-s011.zip › Supplementary File for Cell Morphology Images/ck-10×.jpg]

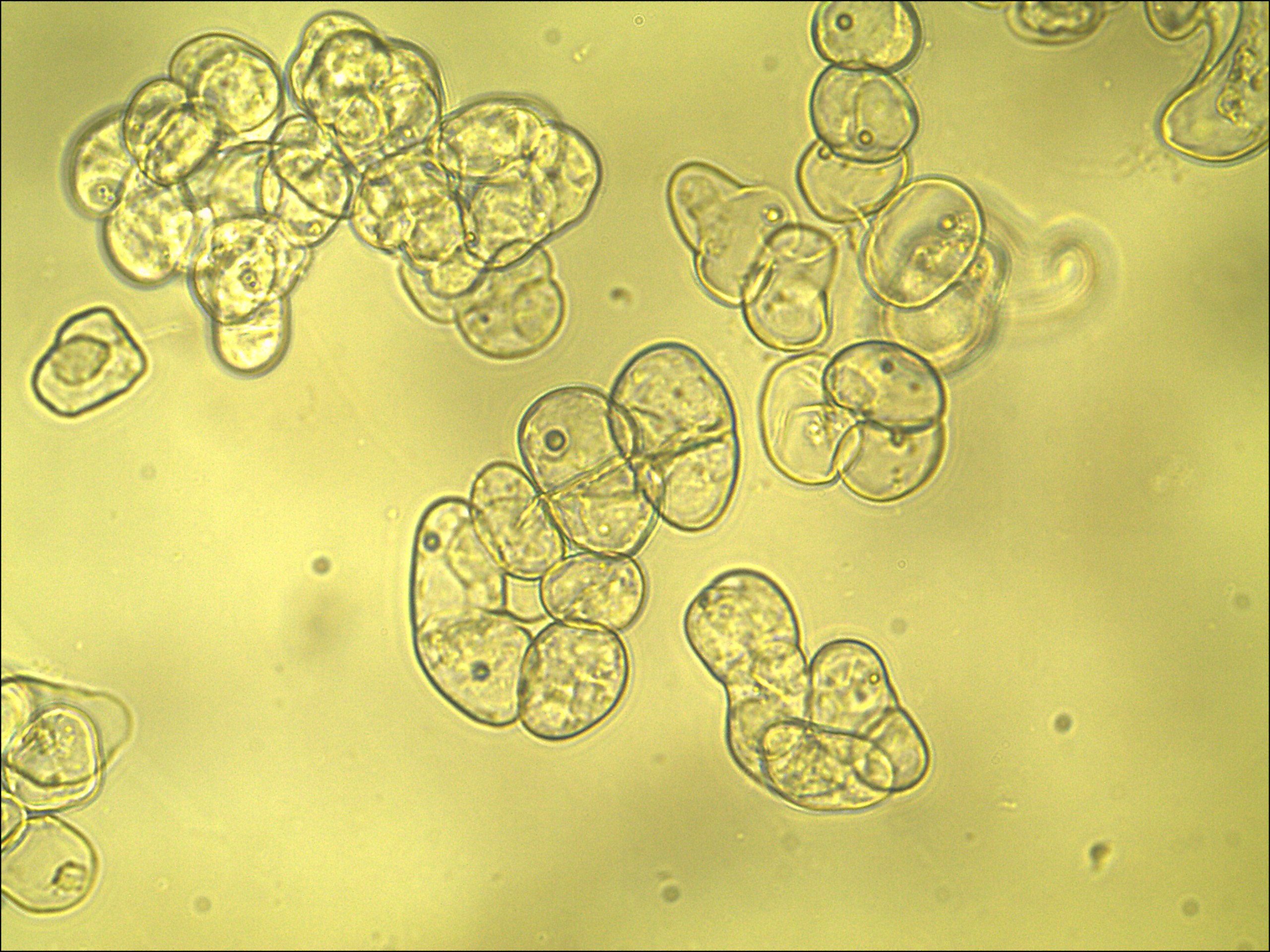

Supplement: Supplemental Information 11 [file peerj-14-21396-s011.zip › Supplementary File for Cell Morphology Images/ck-40x.jpg]
