# Supplementary material for: A cellular view of drought adaptation in sugarcane: multi-omics integration reveals a quadruple module network linking water regulation, oxidative defense, cell wall remodeling, and cell cycle regulation
Source: PeerJ. 2026 Jun 17;14:e21396. doi: 10.7717/peerj.21396 (PMC13282945; doi:10.7717/peerj.21396)
Supplement: Supplemental Information 4 [file peerj-14-21396-s004.doc]

TABLE 1 | Real-time quantitative fluorescence gene primer sequences

| Gene Name | Forward primer（5´→3´） | Reverse primer（5´→3´） | Size(bp) |
| --- | --- | --- | --- |
| GAPDH | GAGTCCACTGGTCGTACCA | GTCTTCTGGGTGGCAGTG | 120 |
| UBQ | GAAGTACCCATTCCCTGAGG | GCTTGCTGATAAAACTGAAGG | 140 |
| PIP1-5 | GCTGGTCTACTGCACGGCGG | GATGGTGTCCGTTCCATGCG | 165 |
| TIP3-1 | ACGAGCACGGCGGGCGGGCT | GCCGTAGTAGGCGTACATGA | 153 |
| XTH8 | ACGGACGGCACCGTCCGCGA | GGTAGTGGTGGCGTCGGCGG | 147 |
| XTH23 | GGGGAAGTTGTCGCGAGCGC | GCCGATGCGGGTGTACGCCG | 172 |
| CSLD5 | GACACCCGTCAATTCAACGA | CGGCGATCTTACGCTCGCGA | 169 |
| PER25 | GGATTCTACGACTCCTACTG | GATGCTCATCCTCACACCATG | 159 |
| PER56 | CCGATTGCTAATGTCGCTGG | GCATCCGCATGGCAGTGTAG | 177 |
| APX1 | GAACTACCCGACCGTGAGCG GAACTACCCGACCGTGAGCG | GTCGGAGCTTGAGGTGGGCCT | 161 |
| APX2 | GGCCTACCCCACGGTGAGCG | TTCAGCCACTCAGAAGTTCC | 156 |
