# Supplementary material for: A cellular view of drought adaptation in sugarcane: multi-omics integration reveals a quadruple module network linking water regulation, oxidative defense, cell wall remodeling, and cell cycle regulation
Source: PeerJ. 2026 Jun 17;14:e21396. doi: 10.7717/peerj.21396 (PMC13282945; doi:10.7717/peerj.21396)
Supplement: Supplemental Information 5 [file peerj-14-21396-s005.doc]

**TABLE 2 |** Statistical table of sample sequencing data evaluation

| Sample | Raw reads | Raw bases | Clean reads | Clean bases | Error rate | Q20 | Q30 | GC pct | Total map | Unique map |
| --- | --- | --- | --- | --- | --- | --- | --- | --- | --- | --- |
| DT_1_ | 43276890 | 6.49G | 41850852 | 6.28G | 0.02 | 96.68 | 91.08 | 54.33 | 77.28% | 39.13% |
| DT_2_ | 44881004 | 6.73G | 43260618 | 6.49G | 0.01 | 96.93 | 91.64 | 53.89 | 76.45% | 38.23% |
| DT_3_ | 41265102 | 6.19G | 40045224 | 6.01G | 0.02 | 96.35 | 90.16 | 54.42 | 77.94% | 40.19% |
| CK_1_ | 43084706 | 6.46G | 41880772 | 6.28G | 0.02 | 96.5 | 90.62 | 53.96 | 76.64% | 38.97% |
| CK_2_ | 42521250 | 6.38G | 41580856 | 6.24G | 0.02 | 96.49 | 90.61 | 54.33 | 77.77% | 39.76% |
| CK_3_ | 43401714 | 6.51G | 42296550 | 6.34G | 0.02 | 96.65 | 90.9 | 54.48 | 78.21% | 39.86% |
| Total |  | 38.76G |  | 37.64G |  |  |  |  |  |  |
