# Supplementary material for: A cellular view of drought adaptation in sugarcane: multi-omics integration reveals a quadruple module network linking water regulation, oxidative defense, cell wall remodeling, and cell cycle regulation
Source: PeerJ. 2026 Jun 17;14:e21396. doi: 10.7717/peerj.21396 (PMC13282945; doi:10.7717/peerj.21396)
Supplement: Supplemental Information 6 [file peerj-14-21396-s006.doc]

TABLE 3 | Drought-related differentially expressed 21 candidate genes in sugarcane suspension cells

| Gene ID | Gene Name | Gene Expression Level |  | Differential Expression Fold |
| --- | --- | --- | --- | --- |
|  |  | DT Gene Expression Level | CK Gene Expression Level |  |
| Aquaporin Protein Family (AQPs) |  |  |  |  |
| ROC_So_Chr03B0012710 | *NIP1-1* | 9.77 | 79.97 | -3.04 ↓ |
| Ctg_00299990 | *PIP1-5* | 0.69 | 27.54 | -5.32 ↓ |
| YZ_Ss_Chr05A0010210 | *PIP2-1* | 45.00 | 304.76 | -2.76 ↓ |
| YZ_Ss_Chr01A0007450 | *TIP3-1* | 391.24 | 38.57 | 3.34 ↑ |
| YZ_Ss_Chr02A0004430 | *NIP1-2* | 59.86 | 182.95 | -1.61 ↓ |
| Cell Wall Dynamic Regulation Genes |  |  |  |  |
| YZ_Rec_Chr02A0001630 | *CESA1* | 47.84 | 115.04 | -1.27 ↓ |
| YZ_Ss_Chr08A0015770 | *XTH8* | 10.26 | 96.62 | -3.23 ↓ |
| ROC_Rec_Chr04B0027150 | *XTH23* | 39.76 | 8.167 | 2.28 ↑ |
| ROC_Rec_Chr01A0010140 | *XTH30* | 187.28 | 86.52 | 1.11 ↑ |
| YZ_So_Chr04B0014250 | *CSLD5* | 29.50 | 9.185 | 1.68 ↑ |
| Peroxidase Family (PER) |  |  |  |  |
| YZ_Rec_Chr05A0001170 | *PER1* | 27.60 | 231.94 | -3.07 ↓ |
| YZ_Rec_Chr05A0049310 | *PER2* | 38.57 | 495.14 | -3.68 ↓ |
| ROC_Rec_Chr04B0025980 | *PER3* | 5.27 | 96.22 | -4.20 ↓ |
| YZ_Rec_Chr03A0010470 | *PER24* | 3.08 | 61.84 | -4.32 ↓ |
| YZ_Rec_Chr02A0002050 | *PER25* | 36.42 | 8.455 | 2.10 ↑ |
| ROC_Rec_Chr04B0000660 | *PER42* | 39.93 | 328.69 | -3.04 ↓ |
| Ctg_00130940 | *PER50* | 28.98 | 171.59 | -2.57 ↓ |
| YZ_Rec_Chr01A0004770 | *PER56* | 0.33 | 47.43 | -7.00 ↓ |
| YZ_So_Chr01C0034050 | *PER70* | 5.96 | 126.24 | -4.42 ↓ |
| Antioxidant and Stress Response Genes |  |  |  |  |
| YZ_Rec_Chr01B0046970 | *APX1* | 68.50 | 0.91 | 6.22 ↑ |
| ROC_So_Chr05A0012130 | *APX2* | 312.06 | 120.90 | 1.37 ↑ |
